# Phase III, randomized, double-blind, placebo-controlled clinical study: a study on the safety and clinical efficacy of AZVUDINE in moderate COVID-19 patients

**DOI:** 10.3389/fmed.2023.1215916

**Published:** 2023-10-19

**Authors:** Sávio Bastos de Souza, Paula Gebe Abreu Cabral, Renato Martins da Silva, Raul Ferraz Arruda, Sheila Passos de Figueiredo Cabral, Arícia Leone Evangelista Monteiro de Assis, Antônio Brazil Viana Junior, Wim Maurits Sylvain Degrave, Aline dos Santos Moreira, Cléber Glória Silva, Junbiao Chang, Pingsheng Lei

**Affiliations:** ^1^High Complexity Center, Galzu Institute, Campos dos Goytacazes, Brazil; ^2^UFC/Ebserh University Hospital Complex, Fortaleza, Brazil; ^3^Functional Genomics and Bioinformatics Laboratory, Oswaldo Cruz Institute – FIOCRUZ, Rio de Janeiro, Brazil; ^4^Santa Casa de Misericórdia de Campos Hospital, Campos dos Goytacazes, Brazil; ^5^NMPA Key Laboratory for Research and Evaluation of Innovative Drug, Henan Normal University, Xinxiang, China; ^6^Institute of Material Medical, Chinese Academy of Medical Sciences and Peking Union Medical College, Beijing, China

**Keywords:** COVID-19, SARS-CoV-2, AZVUDINE, FNC, viral load

## Abstract

**Background:**

In 2019, a highly pathogenic coronavirus named severe acute respiratory syndrome coronavirus 2 (SARS-CoV-2) surfaced and resulted in the outbreak of coronavirus disease 2019 (COVID-19). With the aim of finding effective drugs to fight against the disease, several trials have been conducted since COVID-19 can only be considered a treatable disease, from a clinical point of view, after the availability of specific and effective antivirals. AZVUDINE (FNC), initially developed for treating HIV, is a potential treatment for COVID-19 as it has the capability to lower the patient’s viral load and promote recovery.

**Methods:**

Volunteers infected with SARS-CoV-2 confirmed by reverse transcription polymerase chain reaction (RT-PCR), with good kidney and liver function, who were not using other antivirals or monoclonal antibodies were eligible. Samples from patients were assessed for viral load every 48 h during treatment using reverse transcription quantitative polymerase chain reaction (RT-qPCR) and droplet digital polymerase chain reaction (ddPCR).

**Results:**

The study’s primary outcome measure was the percentage of participants showing an improvement in clinical scores, while the secondary outcome measure was the percentage of participants with a clinical outcome of cure. These measures were used to assess the safety and efficacy of FNC for treating COVID-19. In the analysis of sociodemographic variables, no significant differences were detected between patients in the FNC and the placebo group for race, age group, or sex. The results showed a potential benefit to participants who received FNC during the study, as observed in the shorter hospital stay, shorter negative conversion time of SARS-CoV-2, and a significant reduction in viral load. Furthermore, the reduction in fever and chills were significant at D1, D2, and D3. In this study, a total of 112 adverse events cases were noted, with 105 cases being categorized as non-serious and only 7 cases as serious adverse events.

**Conclusion:**

The pandemic is not being effectively controlled and is causing multiple waves of infection that require extensive medical resources. However, FNC has demonstrated potential to reduce the treatment duration of moderate COVID-19 cases, thereby saving significant medical resources. This makes FNC a promising candidate for COVID-19 treatment.

**Clinical trial registration**: [clinicaltrials.gov], identifier [NCT04668235].

## 1. Introduction

Severe acute respiratory syndrome coronavirus 2 (SARS-CoV-2) is a novel coronavirus that causes coronavirus disease 2019 (COVID-19). The disease has rapidly spread around the world, with high transmission rates and substantial mortality rates (1–3). COVID-19 symptoms vary from mild respiratory illness to severe progressive pneumonia, multiple organ failure, and death (3, 4). As antivirals are key to treating COVID-19, trials have been conducted to identify effective drugs (4).

Several antiviral drugs have been investigated for the treatment of COVID-19, but some have shown adverse effects such as nephrotoxicity and hepatoxicity. For instance, remdesivir has been associated with these adverse events in patients with COVID-19 (5, 6). Additionally, drugs such as favipiravir and molnupiravir have been reported to significantly increase the number of mutations in the RNA structure (7).

Nucleoside antiviral drugs are known for their high efficacy in inhibiting the activity of virus DNA-dependent DNA polymerases (DdDps), RNA-dependent DNA polymerases (RdDps), and RNA-dependent RNA polymerases (RdRps), resulting in the inhibition of viral replication and a high drug resistance barrier (8). The use of FNC (AZVUDINE) in treating mild and common COVID-19 has shown promising results, as it has been found to potentially shorten the nucleic acid negative conversion (NANC) time compared with standard antiviral treatment, expedite viral elimination, and maintain the vital signs of the patients (9).

In the assessment of COVID-19, viral load progression is a crucial aspect. Liu et al. (10) observed that severe cases exhibited higher viral loads compared with mild cases, and a higher viral load corresponded to an increased risk of incubation and death (11). Furthermore, Fajnzylber et al. (12) demonstrated that viral load was associated with COVID-19 severity and mortality. A univariate survival analysis illustrated a significant difference in the probability of survival between individuals with high viral load and those with low viral load (13).

This study was one of the first studies to quantify viral load [absolute quantification by droplet digital polymerase chain reaction (ddPCR)], every 48 h, establishing information on viral load behavior and course of infection. The mean times of the NANC were measured in the FNC and the placebo groups, and the nephrotoxicity and hepatoxicity were monitored.

## 2. Results

### 2.1. Inclusion and exclusion criteria

The study enrolled patients who met specific criteria, including: (1) being at least 18 years old, regardless of gender; (2) testing positive for SARS-CoV-2 nucleic acid through RT-PCR of respiratory or blood samples or highly homologous with known SARS-CoV-2 through viral gene sequencing of respiratory or blood samples; and (3) confirmation of COVID-19 according to the diagnostic criteria outlined in the “latest clinical guidelines for novel coronavirus” issued by the World Health Organization (WHO) on 28 January 2020. All patients who met these criteria were required to sign informed consent forms (ICFs), and those with moderate COVID-19 were admitted to the hospital for treatment. After patients signed the informed consent forms, randomization was performed; thus, the treatment was initiated (D1 of the study), for both the FNC and placebo groups.

The exclusion criteria for this study encompassed several factors, including (1) any known or suspected allergy to the components of FNC tablets; (2) patients with malabsorption syndrome, gastrointestinal absorption issues, an inability to take oral medication, or who require intravenous nutrition; (3) patients currently undergoing anti-HIV treatment; (4) patients experiencing respiratory failure requiring mechanical ventilation, shock, or ICU monitoring/treatment for organ failures; (5) pregnant or lactating women, as well as those with plans for giving birth during the trial period or within 6 months after its completion; (6) individuals who participated in other clinical trials or used experimental drugs within 12 weeks prior to the study; and (7) patients deemed unsuitable for participation in the experiment based on the judgment of the researcher.

The definition of moderate COVID-19 was patients with fever, poor general condition, severe myalgia, persistent dry cough, diarrhea, moderate dyspnea without hypoxia (SpO_2_ 93–94%/TC <50%) or with hypoxia (SpO_2_ 92–93%/TC >50%), and with hospital admission recommended.

There was the presence of comorbidities among the participants, among them the most common were: arterial hypertension, obesity, type 2 diabetes, and alcohol consumption (Supplementary Table S3).

### 2.2. Demographic analysis

**Figure 1:**
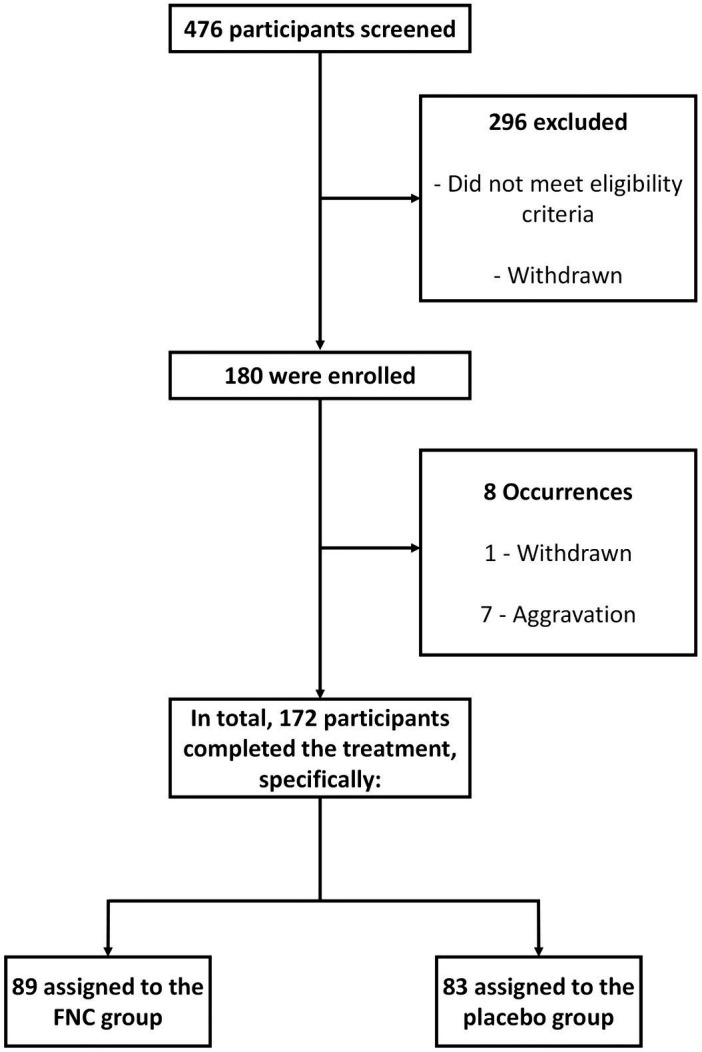
Trial profile.

Between April 2021 and May 2022, a total of 476 individuals were considered for inclusion in this study. Among them, 296 participants were excluded due to various reasons, including not meeting the eligibility criteria, experiencing worsening symptoms prior to transfer to the research center ward, or withdrawing from the clinical trial before participation. Ultimately, 180 participants were randomized, with 172 successfully completing the treatment, while 8 individuals experienced serious adverse events during the course of the study. Of these cases, seven were due to disease progression (referred to the ICU) and one due to previous disease (mitral regurgitation with surgical indication) unrelated to FNC (Figure 1 and Tables 1, 2).

**Table 1:** Demonstration of aggravated cases during the study days.

	Treatments		
Proportion of ICU aggravations	Total	FNC	Placebo
Hospital discharge	172 (95.6%)	88 (96.7%)	83 (94.3%)
ICU aggravation	7 (3.9%)	3 (3.3%)	4 (4.5%)
Dropout	1 (0.6%)	0 (0%)	1 (1.1%)

**Table 2:** General data of cases of aggravation referred to the ICU.

ID	Hospitalization date	Aggravation date (ICU)	Result date	Final result
R19	12/05/2021	15/05/2021	02/06/2021	Death
R26	19/05/2021	20/05/2021	02/06/2021	Death
R41	29/05/2021	08/06/2021	20/06/2021	Death
R79	22/06/2021	26/06/2021	27/06/2021	Death
R115	24/07/2021	26/07/2021	15/08/2021	Death
R149	28/08/2021	29/08/2021	15/10/2021	Hospital discharge
R161	09/09/2021	22/09/2021	28/09/2021	Death

Patient demographics and baseline characteristics were well-matched between the FNC group and the control group at enrollment (Table 3). The median age was 48 years (IQR 41–58), and there was no significant difference between the age of participants who used the FNC and the placebo (*p* = 0.135). The largest number of participants was male, totaling 104 individuals (58%), there were no significant differences concerning gender (*p* = 0.075), indicating that the results obtained were not influenced by the age of the individuals or by gender (Table 3).

**Table 3:** Demographic and baseline characteristics of participants.

		Treatments		
Overall	N	FNC, *N* = 91^1^	Placebo, *N* = 88^1^	*p*-value^2^
Age	179	51 ± 13 (48)	48 ± 13 (48)	0.135
Race	179			0.417
White		48 (53%)	51 (58%)	
Black		18 (20%)	11 (12%)	
Brown		25 (27%)	26 (30%)	
Gender	179			0.075
F		44 (48%)	31 (35%)	
M		47 (52%)	57 (65%)	

^1^Mean ± SD (Median); n (%).

^2^Wilcoxon rank sum test; Pearson’s Chi-squared test.

### 2.3. Clinical improvement

The data indicated that the initial clinical score of the participants who used the FNC was 4.42 ± 0.50 and for those in the control group it was 4.50 ± 0.50, with no significant difference in the clinical scores at which the participants entered the treatment (*p* = 0.298) (Supplementary Figures S1, S2).

Upon clinical discharge, the majority of participants achieved a clinical score of 0 or 1 on the WHO Ordinal Scale of Clinical (14) Improvement, with the exception of one patient who withdrew and seven patients who experienced worsening symptoms. Participants who used the FNC had a final score of 0.02 ± 0.15, while those who participated in the control group had a score of 0.11 ± 0.31, with a statistically significant difference between the groups (*p* = 0.024) (Table 4 and Supplementary Figures S1, S2).

**Table 4:** Overall results between FNC and placebo treatments on study outcomes.

	Treatments		
Objectives and outcomes	FNC	Placebo	*p*-value
Initial score	4.42 ± 0.50 (4.0)	4.50 ± 0.50 (4.5)	*p* = 0.300
Final Score	0.02 ± 0.15 (0.0)	0.11 ± 0.31 (0.0)	***p* = 0.024**
Temperature normalization—fever reduction (number of days)	0.13 ± 0.50 (0.00)	0.38 ± 0.68 (0.00)	***p* < 0.001**
Cure time/absence of viral RNA	7.7 ± 3.6 (6.5)	8.9 ± 3.5 (8.0)	***P* = 0.028**

### 2.4. Time to improvement of symptoms

During the study, the time required for participants to recover was determined by assessing the number of days they experienced symptoms. The characteristic symptoms of patients infected with the SARS-CoV-2 virus were evaluated (Table 4 and Supplementary Tables S1, S4). It was not possible to identify statistical differences in the time to the improvement of all symptoms between the two groups, FNC and placebo, of participants, except by the time of improvement of fever (*p* < 0.01) and chill (*p* = 0.08) symptoms.

In the analysis of curing time, it was observed that the FNC group had a shorter cure time/absence of viral RNA (6.5 days, *p* = 0.028) compared with the placebo group (8 days). There was a significant reduction in the length of hospital stay for the FNC group. Nine participants took more than 14 days for the first negative conversion.

### 2.5. Time of the nucleic acid negative conversion

**Figure 2:**
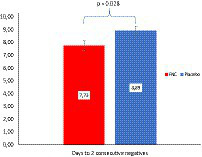
The mean (SD) number of days until the second nucleic acid testing showed negativity compared between the FNC group and the placebo group. The Mann–Whitney test was used to analyze the differences between the groups, with the FNC group represented by a red bar and the placebo group represented by a blue bar.

The duration of negative nucleic acid conversion (NANC) is often used as an indicator of drug efficacy and clinical improvement. In this study, clinical discharge was achieved after two consecutive negative NANC results. The findings revealed that the FNC treatment group had a significantly shorter time to achieve the second negative NANC result (7.73 days, *p* = 0.028), compared with the placebo group (8.89 days) (as shown in Figure 2).

### 2.6. Detection of SARS-CoV-2 viral load by RT-PCR and ddPCR technique

**Figure 3:**
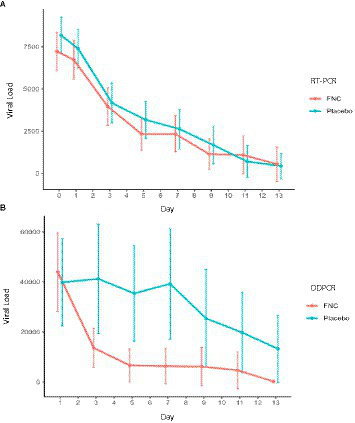
**(A)** Estimated (RT-PCR) and **(B)** absolute viral load analysis (ddPCR) of participants in the FNC group and the placebo group during the treatment days. Data are median (SD). (Red line, FNC; blue line, placebo).

In this study, the FNC group showed a more accentuated increase in cycles threshold (CTs)/day, although without showing significant differences compared with the control group (Supplementary Figure S3A). It was not possible to observe significant differences between the two groups on any of the treatment days. Since it was not possible to notice differences in the results of CTs, the same occurred for the viral load of the participants analyzed by the RT-PCR technique (Table 5 and Figure 3A).

**Table 5:** Estimated (RT-PCR) viral load values during the treatment days.

		Treatments		
Overall	N	FNC, *N* = 91^1^	Placebo, *N* = 88^1^	*p*-value^2^
Viral load (D1)	178	10,398 (1,000; 11,613) [7,227]	10,456 (1,019; 11,324) [8,178]	0.635
Viral load (D3)	172	10,080 (533; 11,752) [6,730]	10,199 (546; 11,434) [7,394]	0.346
Viral load (D5)	166	101 (0; 10,219) [3,961]	970 (2; 10,199) [4,175]	0.331
Viral load (D7)	129	0 (0; 1,004) [2,335]	10 (0; 9,828) [3,173]	0.672
Viral load (D9)	97	5 (0; 1,010) [2,337]	10 (0; 1,026) [2,626]	0.120
Viral load (D11)	72	0 (0; 78) [1,146]	10 (0; 102) [1,677]	0.069
Viral load (D13)	50	0 (0; 102) [1,095]	0 (0; 0) [702]	0.655
CTs (D1)	178	0 (0; 0) [545]	0 (0; 0) [439]	0.686
CTs (D3)	172	24.2 (21.3; 27.0) [23.9]	23.9 (21.6; 26.6) [23.9]	0.299
CTs (D5)	166	24.7 (20.7; 28.1) [24.5]	24.8 (21.5; 27.8) [24.3]	0.334
CTs (D7)	129	28.8 (24.6; 31.0) [27.3]	27.9 (24.5; 30.9) [26.7]	0.685
CTs (D9)	97	31.00 (29.30; 31.00) [29.72]	30.40 (28.60; 31.00) [28.99]	0.119
CTs (D11)	72	31.00 (28.45; 31.00) [29.48]	31.00 (31.00; 31.00) [29.90]	0.062
CTs (D13)	50	31.00 (31.00; 31.00) [30.28]	31.00 (31.00; 31.00) [30.43]	0.655

^1^Median (25%; 75%) [Mean].

^2^Wilcoxon rank sum test.

It was not possible to identify a significant difference in viral load quantified through the RT-PCR technique between FNC and the control group. However, the viral load quantified by ddPCR showed a great difference between the groups (Table 6 and Figure 3B). The high sensitivity of the ddPCR confronts the variability obtained by calculating the viral load by RT-PCR (standard curve calculation due to the logarithmic variability) after treatment with FNC, showing a significant reduction in viral load at D3 (*p* < 0.002), D5, D7, and D9 (*p* < 0.001), and D11 (*p* < 0.006).

**Table 6:** Absolute (ddPCR) viral load values during the treatment days.

		Treatments		
Overall	N	FNC, *N* = 91^1^	Placebo, *N* = 88^1^	*p*-value^2^
DDPCR (D1)	178	6,108 (362; 46,646) [43,988]	4,183 (141; 39,483) [39,861]	0.250
DDPCR (D3)	176	49 (0; 5,638) [13,629]	1,002 (34; 22,471) [41,221]	**0.002**
DDPCR (D5)	155	0 (0; 202) [6,682]	284 (14; 16,827) [35,440]	**<0.001**
DDPCR (D7)	116	0 (0; 0) [6,329]	1,120 (40; 25,230) [39,258]	**<0.001**
DDPCR (D9)	89	0 (0; 0) [6,176]	256 (0; 12,665) [25,426]	**<0.001**
DDPCR (D11)	65	0 (0; 0) [4,681]	0 (0; 1,673) [19,791]	**0.006**
DDPCR (D13)	44	0 (0; 0) [223]	0 (0; 0) [13,273]	0.111

^1^Median (25%; 75%).

^2^Wilcoxon rank sum test.

Notably, it was possible to observe significant differences in the time of improvement of fever at D1 (*p* < 0.015), D2 (*p* < 0.040), and D3 (*p* < 0.026), and chill (*p* = 0.08) symptoms (Table 4). More information can be found in the Supplementary material.

### 2.7. Sequencing of SARS-CoV-2 strains

**Figure 4:**
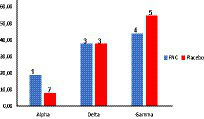
Percentage of volunteers infected by the different strains of SARS-COV-2 distributed among the treatments. (Red bar, FNC; blue bar, placebo).

Here, genetic sequencing was performed to demonstrate the distribution of strains between the FNC and placebo groups. The strain with the lowest prevalence was Alpha, which affected 7.8 and 18.8% of the volunteers who used FNC and the placebo, respectively (Figure 4). The Delta strain affected 37.7% of the volunteers who used FNC and the placebo (Figure 4). The strain with the highest incidence during the research was Gamma, which affected 54.5 and 43.8% of the volunteers who used FNC and the placebo, respectively (Figure 4).

### 2.8. Changes in kidney and liver functions baselines

**Figure 5:**
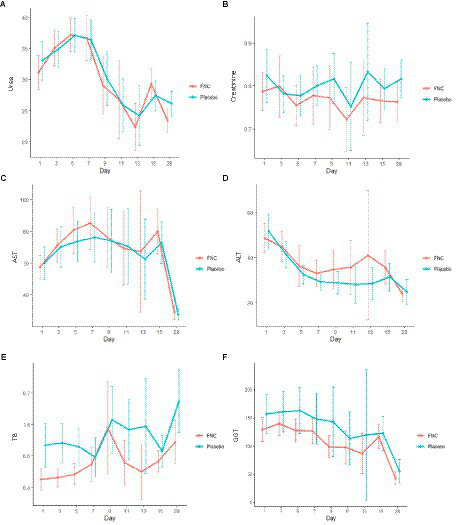
During the treatment, the dynamic changes in kidney and liver markers: **(A)** creatinine, **(B)** urea, **(C)** alanine aminotransferase (ALT), **(D)** aspartate aminotransferase (AST), and **(E)** total bilirubin (TB), and **(F)** gamma-glutamyl transpeptidase (GGT) of the patients in the FNC group and patients in the placebo group. Data are median (SD). (Red line: FNC; blue line: placebo).

The renal function test results of the participants assigned to either the FNC or the placebo group, which included evaluations of creatinine and blood urea nitrogen, exhibited similar value profiles. These values remained within the normal parameters throughout the treatment, and no significant differences were observed between the two groups during the treatment period (Figures 5A,B).

The liver function test results of the participants assigned to either the FNC or the placebo group, which included assessments of aspartate aminotransferase (AST), alanine aminotransferase (ALT), glutamyl transpeptidase, and total bilirubin, revealed that all values were within the normal range. Both groups exhibited similar results profiles, and no statistically significant changes were observed during the course of the treatment. Additionally, the results obtained from the exams related to renal function (Figures 5C–F) were consistent with these findings.

### 2.9. Time and proportion of lung imaging improvement

It was not possible to observe significant differences regarding the improvement of lung images during the treatment days (Supplementary Table S2). All participants started the study with a clinical picture of 25–50% of pulmonary involvement; however, despite the clinical improvement that was observed, the improvement of the lungs occurred slowly, so it was not possible to observe a difference in this parameter due to the short treatment period (14 days) and clinical follow-up at D28 and D60.

#### 2.9.1. Adverse events and clinical safety of FNC

In this study, a total of 113 adverse events were recorded, with 105 categorized as non-serious and only 8 considered serious. Of these cases, seven were due to disease progression and one due to previous disease (mitral regurgitation with surgical indication) unrelated to FNC (Table 7).

**Table 7:** Global quantification of adverse events.

		Treatments			
*N* = 180	Total	FNC	Placebo	Subject (%)	Intensity
Adverse events	105	50	55	58.33	Grade 1 and 2
Frequency and intensity of serious adverse events	8	3	5	4.44	Grade 4
All-cause mortality rate during the study	7	3	4	3.88	4—Death
Frequency and intensity of unexpected adverse events	0	0	0	0	0
Occurrence of drug interactions	0	0	0	0	0

The adverse events observed in this study were mainly related to the increase in ALT (45 cases), GGT (13 cases), AST (10 cases), all being grade 1 intensity, considering that they occurred within the normal range. It was also possible to observe an increase in ALT, GGT, and AST at the time of randomization, which is to be expected in infectious conditions. The adverse reactions observed in this study were the same as those related to antiviral drugs, with no unexpected adverse reactions occurring (Table 8).

**Table 8:** Consolidated report of adverse events.

Classification	Grade 1		Grade 4		Grade 1			Grade 4
	Case	Subject (%)	Case	Subject (%)	Case	Subject (%)	Case	Subject (%)
ALT elevation	**24**	26.37	0	0	**21**	23.59	0	0
GT range lifting	**6**	6.59	0	0	**7**	7.86	0	0
AST elevation	**4**	4.39	0	0	**6**	6.74	0	0
Headache	**2**	2.19	0	0	**6**	6.74	0	0
Phlebitis MS	**1**	1.09	0	0	**3**	3.37	0	0
GT gamma reduction	**1**	1.09	0	0	**2**	2.24	0	0
High fever	**2**	2.19	0	0	**1**	1.12	0	0
Dizziness	**0**	0	0	0	**2**	2.24	0	0
Sodium reduction	**0**		0	0	**2**	2.24	0	0
Potassium reduction	**1**	1.09	0	0	**2**	2.24	0	0
Hemoglobin reduction	**1**	1.09	0	0	**1**	1.12	0	0
Hyperglycemia	**1**	1.09	0	0	**1**	1.12	0	0
Hypoglycemia	**1**	1.09	0	0	**0**	0	0	0
Calcium reduction	**0**	0	0	0	**0**	0	0	0
Creatinine increase	**1**	1.09	0	0	**0**	0	0	0
CPK increase	**0**	0	0	0	**1**	1.12	0	0
Troponin I increase	**1**	1.09	0	0	**0**	0	0	0
Backache	**0**	0	0	0	**1**	1.12	0	0
Diarrhea	**1**	1.09	0	0	**0**	0	0	0
Tachycardia	**1**	1.09	0	0	**0**	0	0	0
Nauseas	**1**	1.09	0	0	**0**	0	0	0
Respiratory insufficiency	**0**	0	**3**	3.29	**0**	0	**4**	4.49
Severe mitral insufficiency	**0**	0	**1**	1.09	**0**	0	0	0
Leukopenia	**1**	1.09	0	0	**0**	0	0	0
Total	**50**	54.81	**4**	4.38	**56**	62.86	**4**	4.49

Phlebitis that occurred during the study was due to the administration of intravenous antibiotics, which was later changed to oral administration. There was also no significant change in urinary phosphorus. In preliminary studies, vertigo (incidence ≥5%) has been attributed to FNC; however, in this study, there were only two reported cases of dizziness related to labyrinthitis (history) and hypoglycemia (due to loss of taste). It should also be considered that the participants were bedridden, which could potentiate these events.

There were seven exclusions due to the disease worsening and progression to the ICU. There were six deaths and one recovery where participants received adequate care and support during hospitalization. In the case of deaths, three participants arrived at the hospital with a worsening condition, and after admission they were transferred to the ICU within 1–3 days (Table 7).

There was also no significant change in urinary phosphorus as reported in the adverse events of special interest (Supplementary Table S5). Serum cholinesterase is decreased in hepatic parenchymal diseases (e.g., viral hepatitis and cirrhosis), congestive heart failure, abscesses, neoplasms, malnutrition, acute infections, anemia, myocardial infarction, and dermatomyositis. It may be increased in obese patients, diabetics, and those with nephrotic syndrome. We observed that the values evaluated were not significant and that the participants did not have other debilitating conditions (Supplementary Table S6).

There was no significant difference in the inflammatory marker values during the study days (Supplementary Table S7). There was a reduction of leukocytes and neutrophils within the normal range but significant on D1 and compatible with an initial stage of infection (Supplementary Table S8). Procalcitonin has good sensitivity and specificity for the diagnosis of secondary bacterial infections in patients with viral diseases and was not significant at D1, which meets the protocol’s inclusion criteria, becoming significant at D7 for 102 participants, reducing up to D15 (medical discharge) and D28 (follow-up after medical discharge).

There was a significant increase in lymphocytes at D13 for 51 participants, but within the normal range (Supplementary Table S9). There was the significance of CD8 at D11 (elimination of infectious cells) for 63 participants. There was CD4 significance at D28 (specific for opportunistic infections) for 128 participants, which may explain post-covid symptoms.

Although there was an improvement in respiratory symptoms leading participants to hospital discharge, this improvement was not observed in the statistical analysis (Supplementary Table S10).

Although there was an improvement in O_2_ saturation, this was not observed in the statistical analysis. There was an improvement in O_2_ saturation in the two groups from D2 onwards (Supplementary Table S11). The improvement in respiratory rate was not significant and ventilatory support makes this parameter questionable (Supplementary Table S12).

Supplementary Table S13 shows the number of participants who entered the study without the need for supplemental oxygen supply (room air), or with the need for supplementary oxygen supply (nasal catheter and reservoir mask), with significance between groups, at D1. It also shows that the number of these participants who moved to room air practically doubled at D2 in the FNC group. The predominance of recovery to room air continued until D6, which coincided with the time when the viral load decreased with FNC on D3, D5, and D7. Also, the number of participants using a catheter or reservoir mask from D2 onwards was lower in the FNC group than in the placebo group. Also related to the frequency of supplemental oxygenation or non-invasive ventilation, we have the number of liters of O_2_/min that tended to decrease in the FNC group (Supplementary Table S14).

There was no significance between the groups in the use of mechanical ventilation, although there was a predominance of worsening conditions in the placebo group. Another factor is the speed of evolution of the clinical condition due to the disease, since a moderate patient could progress in a few hours to a severe clinical condition, requiring admission to the ICU, which justifies entry into the study (moderate clinical condition) and subsequent worsening (admission to the ICU) (Supplementary Table S15).

## 3. Discussion

Patient demographics data indicated that the results obtained were not influenced by the age of the individuals or by gender (Table 3). The present study demonstrated no significant difference in the time to improvement of all symptoms between participants who received FNC and those who received the placebo. These findings are consistent with a previous pilot study by Ren et al. (9), which also reported no differences in symptoms and laboratory test results during screening between the FNC and control groups. However, it was possible to observe significant differences in the time of improvement of fever (*p* < 0.01) and chill (*p* = 0.08) symptoms. The initial sensation of coldness during fever may be attributed to vasoconstriction leading to a decrease in skin temperature (3); thus, chill and fever are correlated. Since fever attenuation was observed in the FNC group, this may be a consequence of the decline in the infection (4), which in turn is related to a possible reduction of the patient viral load.

Another point to be highlighted is that there was a significant reduction in the length of hospital stay for the FNC group, reducing the time of exposure to the virus action and the possibility of greater sequelae. Nine participants took more than 14 days for the first negative conversion. Concomitant with these data, the NANC time was significantly shorter in participants treated with FNC (7.73 days, *p* = 0.028) compared with those treated with the placebo (8.89 days), as shown in Figure 2, which is consistent with the findings of Ren et al. (9) which demonstrated that FNC treatment may shorten the NANC time in mild and common COVID-19 cases when compared with standard antiviral treatment. Thus, FNC treatment may reduce the treatment duration for mild patients and, consequently, save valuable medical resources.

Several studies have reported a relationship between viral loads and disease severity (15–18). For instance, Liu et al. (10) found that severe COVID-19 cases had higher viral loads than mild cases, and it has also been shown that higher viral loads are associated with an increased risk of incubation and death (11). Additionally, Fajnzylber et al. (12) reported that viral load is implicated in the severity and mortality of COVID-19. A significant difference in survival probability was observed between patients with high viral load and those with low viral load based on a univariate survival analysis (13). A recent randomized clinical trial investigated the effectiveness of FNC added to standard treatment compared with a placebo group for patients with mild COVID-19 (19). The findings suggest that FNC treatment may shorten the time of the nucleic acid negative conversion and reduce viral load in these patients (19).

In the present study, it was not possible to identify a significant difference in viral load quantified through the RT-PCR technique between the FNC and the control group. RT-PCR is considered the gold standard for the diagnosis of COVID-19, but its reliability has been questioned due to negative results in some clinically suspected patients and positive results in recovered patients (2, 20). Moreover, RT-PCR results can be influenced by viral RNA sequence variations, and sampling procedures can contribute to a high false-negative rate due to differences in viral load across anatomical sites (21). In real COVID-19 cases, one-time testing can result in a false-negative rate as high as 30–50% (21).

According to Yu et al. (18), although RT-PCR is sensitive and reliable for detecting SARS-CoV-2, ddPCR performs better in detecting low-viral-load samples. In their study, the results of RT-PCR and ddPCR were consistent in the 95 positive samples, and the Ct value of RT-PCR was highly correlated with the copy number value of ddPCR. However, when Ct values were between 34 and 38, the viral load of samples with the same Ct value was significantly different, indicating that the Ct value of RT-PCR may not sensitively reflect the level of viral load when the viral load is low. In our study, ddPCR quantified a significantly higher viral load than RT-PCR between the treatment groups (Table 6 and Figure 3B), which is consistent with previous studies that showed ddPCR’s advantage of absolute quantification and higher sensitivity for virus detection than RT-PCR (18, 22, 23).

In addition, the sequencing of SARS-CoV-2 for the detection of potential lineages was performed. The distribution of strains between the FNC and the placebo groups showed that the strain with the highest incidence during the research was Gamma, which affected 54.5 and 43.8% of the volunteers who used the FNC and placebo, respectively (Figure 4). The variant omicron had not appeared during the period in which the study was carried out (24). This study had only six vaccinated participants, three in the placebo group and three in the FNC group. This study was carried out in a period when vaccines were not widely available for the population, and therefore vaccine interference may exist in only three vaccinated participants infected by the Delta strain variants AY.99.1, AY 0.99.1, and AY.99.2, respectively.

In this study, the treatment with FNC was well tolerated by patients. Vital signs, liver function, and kidney function in both groups were normal. These data reinforce what was observed in the pilot clinical trial previously performed with FNC, in which hepatic and renal functions did not change between the FNC and the control group, indicating the non-toxicity of the drug. This is not the case for many antivirals; in studies with remdesivir, for example, nephrotoxicity and hepatoxicity were reported as adverse drug events in patients with COVID-19 (5, 6). It was reported that similar types of antiviral drugs may cause mitochondrial injury in renal tubular epithelial cells (6, 21). Therefore, our results highlight the safety of FNC since no changes were observed in markers of kidney and liver damage when the two groups were compared.

The adverse reactions identified in this study were consistent with those commonly associated with antiviral medications, and no unexpected adverse reactions were reported (Table 8). The analysis of adverse events between the FNC and the placebo groups showed a similar incidence rate, indicating that adverse events observed were likely a result of the underlying disease and not due to the treatment.

The analysis of the viral load, every 48 h, served as a safety examination that could identify the intensity of infection of individuals, being a marker in the prevention of worsening (a condition that, when it occurs, excludes the participant from the study). Verifying viral load enabled patient management, preventing worsening and allowing safety parameters to be better assessed.

To summarize, administering FNC to moderate COVID-19 patients may lead to a faster conversion to nucleic acid negativity compared with the placebo group, which could potentially reduce hospitalization duration and improve clinical outcomes. FNC treatment accelerates viral clearance, leading to a significant decrease in viral load and symptom relief. These findings support the use of FNC in the treatment of moderate COVID-19 patients. Since FNC is an oral drug that is excreted within 24 h without integration into human genetic material, it offers a safe and effective treatment option that can help reduce the time and cost of COVID-19 treatment and control the pandemic’s spread.

## 4. Methodology

### 4.1. Study design

This clinical trial was conducted at Santa Casa de Misericordia de Campos Hospital as a strategic decision to ensure the standardization and quality of molecular biology analyses. Each RT-PCR equipment and reagent kit used in RT-PCR has different sensitivities and performance, hence the need to concentrate the analyses. The study was a double-blind, placebo-controlled clinical trial with randomization and was approved by the institutional review board of the National Health Surveillance Agency (CE 0937457/21–4) and the National Council for Research Ethics (CAAE 52176421.8.0000.5244). The trial was registered on clinicaltrials.gov (NCT04668235) under the title “Study on Safety and Clinical Efficacy of AZVUDINE in COVID-19 Patients (SARS-CoV-2 Infected).” All participants provided written informed consent, and the inclusion and exclusion criteria, design, goals, and outcomes are detailed in Supplementary methodology.

Patients assigned to the FNC group received standard treatment along with oral FNC tablets at a dosage of 5 mg (five tablets administered once a night). This concentration was based in the previous randomized controlled clinic study of FNC tablets in the Treatment of Mild and Common COVID-19 (9). The mean half-life of FNC at this dosage is 13.8 h, with both the intact drug and its metabolites excreted in the urine within 24 h. In the control group, patients were administered a placebo in addition to standard treatment. Details of the inclusion and exclusion criteria can be found in the results and discussion section. The placebo tablets were physically identical to the FNC tablets, containing microcrystalline cellulose, hydrated lactose, polyvinylpyrrolidone K30, croscarmellose sodium, and magnesium stearate.

Standard treatment: All participants received the treatment for COVID-19 prescribed by the Ministry of Health in Brazil. Medications include ceftriaxone disodic, 1,000 mg/mL fras; omeprazole, 40 mg-vial-amp 10 mL inject.; ondansetron, chloridate, 2 mg/mL; dipiron sodic, 500 mg/mL ampoule 2 mL ii; formoterol fumarate 12 mcg + budesonide 400 mcg; dexamethasone, 4 mg/mL ampoule 2.5 mL in.; enoxaparin; 40 mg/0.4 mL inject. Syringe; captopril, 50 mg-tablet orally; losartan potassium, 50 mg tablets; clarithromycin, 500 mg tablet orally; clonazepam, 2 mg tablet orally; ceftriaxone disodic, 1,000 mg/mL fras; omeprazole, 40 mg vial-amp 10 mL inject.; ondansetron, 2 mg/mL chloridate; dipiron sodica, 500 mg/mL ampoule 2 mL ii; formoterol fumarate 12 mg + budesoni; dexamethasone, 4 mg/mL ampoule 2.5 mL in; enoxaparin, 40 mg/0.4 mL inject. Syringe; captopril, 50 mg tablet orally.

Enrollment: Once patients provided their informed consent by signing the ICF, a throat swab was collected for RT-PCR nucleic acid testing to confirm the presence of COVID-19. The main investigator assessed whether the patient met the inclusion criteria, and eligible patients with laboratory-confirmed COVID-19 and moderate symptoms were transferred to the hospital for admission.

First patient enrollment: 04/23/2021.

Last patient enrollment: 03/04/2022.

Randomization: The main investigator conducted exams to assess whether the patients met the eligibility criteria after they signed the consent form. If the patients were found to be eligible, they were admitted to the hospital and randomly assigned to either the FNC group or the control group in a 1:1 ratio. Randomization was performed using Software Researcher IGZ v2.0, at the participant’s hospitalization, randomly into the FNC and control groups. In the pharmacy, the already fractionated drug received a bar code, where the system only allowed the drug to be dispensed if the bar code matched the randomization of the participant.

Apart from monitoring the vital signs and performing routine hematology and biochemistry exams, the participants’ SARS-CoV-2 nucleic acid levels were checked by RT-PCR after they commenced their medication. The nucleic acid detection tests were conducted every 48 h during the treatment period to obtain the optimal measurement of the participants’ viral load. Clinical discharge was confirmed when two consecutive negative test results were obtained. These tests were utilized to obtain the average time taken for the nucleic acid to turn negative (NANC).

This study was carried out at the height of the pandemic, in 2021, and the beginning of vaccinations, hence the low vaccination rate in the participants (six people). During this period, the need for ICU care was frequent, as were deaths. And since there was no effective treatment, monitoring the viral load during the course of the disease (every 48 h) could establish viral behavior, the relationship with the clinic, and the efficacy of the experimental therapy. The trial ended on 10 August 2022.

*Outcomes:* The primary outcome was the proportion of participants with improved clinical status. The criterion for a participant to have an improvement in clinical status was a decrease in the WHO Ordinal Scale of Clinical Improvement by at least one category compared with that when screening. Time Frame: Day 1 to Day 15.

*The study’s secondary outcomes included the following:* (1) the proportion of participants who achieved a clinical cure during the study, defined as the absence of viral RNA in collected samples and meeting the clinical criteria for hospital discharge; (2) the time to improvement of symptoms such as diarrhea, myalgia, fatigue, malaise, cough, dyspnea, and headache; (3) changes in liver and kidney function from baseline; (4) the comparison of SARS-CoV-2 viral load negative conversion time by RT-PCR between the FNC group and the control group; (5) length of hospital stay; (6) frequency and intensity of adverse events, unexpected adverse events, and serious adverse events; (7) the all-cause mortality rate during the study; and (8) the evaluation of the tolerability of AZVUDINE (FNC) at a dosage of 5 mg/day for up to 14 days.

The hospital discharge criterion was two consecutive negative results and an improvement in clinical status. However, the treated strains were aggressive (Alpha, Gamma and Delta), for this reason, participants needed to remain hospitalized until the second negative result, for safety reasons due the clinical conditions, in this period, in 2021, were not so simple, in addition to there being a lack of knowledge about the disease. Eleven participants failed to perform the second RT-PCR during hospitalization due to hospital discharge due to clinical improvement. Seven participants failed to perform the second RT-PCR due to being transferred to the ICU. In total, 18 participants skipped the second RT-PCR exam. All participants were included in the statistical analysis except one dropout.

The safety of the participants was continuously monitored throughout the study by tracking vital signs, changes in liver and renal function, and adverse events. The adverse events were evaluated based on their type, incidence, severity, time of occurrence, drug correlation, and severity assessment. Previous research has reported that the use of FNC did not result in any significant adverse events that were drug-related (9).

### 4.2. Statistical analysis

Initially, there were 342 participants in the study. However, due to the decrease in the number of COVID-19 cases in Brazil toward the end of 2021, the sample size was reevaluated and subsequently reduced to 180 participants. These participants were randomly assigned to two study groups, each consisting of 90 participants. All enrolled patients with moderate COVID-19 were hospitalized. The sample calculation was performed using the formula of “sample calculation for superiority studies using proportions,” described by World Health Organization (14). To analyze demographic information and baseline eigenvalues, descriptive statistics such as mean, standard deviation, quartiles, and minimum and maximum values were calculated for numerical variables. Frequency and percentage were determined for categorical data. The appropriate statistical methods were employed to compare the two groups based on the type of indicator. The Mann–Whitney test was utilized to compare quantitative data, while Fisher’s exact test was employed for categorical data. All statistical analyses were conducted using R-studio software.

### 4.3. Quantification of SARS-CoV-2 viral load by reverse transcription–polymerase chain reaction

The MagMAXTM Viral/Pathogen Nucleic Acid Isolation kit (Applied Biosystems) was employed to extract total RNA from nasal and throat swabs obtained from the participants of the clinical study. The extraction process was carried out in accordance with the manufacturer’s guidelines.

Following the extraction of total RNA, RT-PCRs were conducted using the TaqPath^TM^ COVID-19 CE-IVD RT-PCR kit (ANVISA Reg.: 10358940107) on the QuantStudio5 RT-PCR equipment from Applied Biosystems (ANVISA Reg: 10358940069), as per the instructions provided by the manufacturer. The primers and probes chosen were designed to target the ORF1ab and N genes.

To estimate the viral load of each sample, CTs obtained from RT-PCR were plotted on a standard curve created using serial dilutions of the positive control (TaqPath^TM^ COVID-19 Control), which consists of SARS-CoV-2 viral RNA at a known concentration of 1 × 104 copies/μL.

An RT-PCR result is deemed positive when CT values are equal to or lower than 30.5. During the reaction, the specific probe utilized to detect the presence of SARS-CoV-2 is cleaved by DNA polymerase, causing the emission of fluorescence when viral RNA is present. Higher levels of viral RNA generate greater fluorescence, leading to an earlier appearance of the CT value in the reaction. Conversely, lower levels of viral RNA result in lower fluorescence, leading to a delayed appearance of the CT value. CT values above 30.5 are interpreted as negative. By constructing a concentration curve for viral RNA, we can generate a curve of CT values, which ranges from lower values (indicating higher copies of viral RNA) to higher values (indicating lower copies of viral RNA).

### 4.4. Quantification of SARS-CoV-2 viral load by droplet digital polymerase chain reaction

Nasal and throat swabs collected from clinical study participants were subjected to RNA extraction using the MagMAXTM Viral/Pathogen Nucleic Acid Isolation kit (Applied Biosystems) in accordance with the manufacturer’s guidelines. Following the extraction of total RNA, ddPCR was conducted.

PCR amplification was carried out with primers and probes targeting the ORF1ab and N genes, along with a positive reference gene, following the manufacturer’s guidelines for the reaction system and amplification conditions (Shanghai BioGem Medical Technology Co., Ltd., China).

The Targeting One Digital PCR System, which includes the COVID-19 digital PCR detection kit, droplet generation kit, and droplet detection kit, was utilized to conduct digital droplet PCR analyses. The kit was designed to detect the ORF1ab gene, the N gene, and a positive reference gene, with a detection limit of 10 copies/test. Targeting One Technology is authorized by the China Food and Drug Administration. A fractional number represents viral fragments that do not constitute a viral unit.

## Data availability statement

The raw data supporting the conclusions of this article will be made available by the authors, without undue reservation.

## Ethics statement

The studies involving humans were approved by Comissão Nacional de Ética em Pesquisa (Conep). The studies were conducted in accordance with the local legislation and institutional requirements. The participants provided their written informed consent to participate in this study.

## Author contributions

PC coordinated the project and supervised the writing of the manuscript. SS performed the analysis of the data. AV assisted in the acquisition of statistical data. RS, RA, SC, and AS assisted in the acquisition of data. PC, SS and RS wrote the manuscript. WD and AM performed the sequencing analyses. CS assisted the medical team that conducted the clinical research. JC and PL assisted in reviewing the manuscript. All authors read and approved the final version of the manuscript.

## Abbreviations

ALT: Alanine aminotransferase
AST: Aspartate aminotransferase
BT: Bleeding time
COVID-19: Coronavirus disease 2019
FNC: AZVUDINE
GGT: Gamma-glutamyl Transferase
ICFs: Informed consent forms
ICU: Intensive care unit
NANC: Nucleic acid negative conversion
SARS-CoV-2: Severe acute respiratory syndrome coronavirus 2
WHO: World Health Organization

## References

[1] Zhu N Zhang D Wang W Li X Yang B Song J. A novel coronavirus from patients with pneumonia in China, 2019. N Engl J Med. (2020) 382:727–33. doi: 10.1056/NEJMoa2001017, PMID: 31978945 PMC7092803

[2] Wu J Liu J Zhao X Liu C Wang W Wang D. Clinical characteristics of imported cases of COVID- 19 in Jiangsu Province: a multicenter descriptive study. Clin Infect Dis. (2020) 71:706–12. doi: 10.1093/cid/ciaa199, PMID: 32109279 PMC7108195

[3] Wang Y Kang H Liu X Tong Z. Combination of RT-qPCR testing and clinical features for diagnosis of COVID-19 facilitates management of SARS-CoV-2 outbreak. J Med Virol. (2020) 92:538–9. doi: 10.1002/jmv.25721, PMID: 32096564 PMC7233289

[4] Zhang X Horby P Cao B. COVID-19 can be called a treatable disease only after we have antivirals. Sci. Bull. (2022) 67:999–1002. doi: 10.1016/j.scib.2022.02.011, PMID: 35223129 PMC8856962

[5] Beigel JH Tomashek KM Dodd LE Mehta AK Zingman BS Kalil AC. Remdesivir for the treatment of Covid-19—final report. N Engl J Med. (2020) 383:1813–26. doi: 10.1056/NEJMoa2007764, PMID: 32445440 PMC7262788

[6] Adamsick ML Gandhi RG Bidell MR Elshaboury RH Bhattacharyya RP Kim AY. Remdesivir in patients with acute or chronic kidney disease and COVID-19. J Am Soc Nephrol. (2020) 31:1384–6. doi: 10.1681/ASN.2020050589, PMID: 32513665 PMC7351006

[7] Abdelnabi R Foo CS Kaptein SJ Zhang X Langendries L Vangeel L. The combined treatment of Molnupiravir and Favipiravir results in a marked potentiation of efficacy in a SARS-CoV2 hamster infection model through an in- creased frequency of mutations in the viral genome. bioRxiv. (2021):2020–12. doi: 10.1101/2020.12.10.419242 PMC8461366 34571361

[8] Jordheim LP Durantel D Zoulim F Dumontet C. Advances in the development of nucleoside and nucleotide analogues for cancer and viral diseases. Nat Rev Drug Discov. (2013) 12:447–64. doi: 10.1038/nrd4010, PMID: 23722347

[9] Ren Z Luo H Yu Z Song J Liang L Wang L. A randomized, open-label, controlled clinical trial of AZVUDINE tablets in the treatment of mild and common COVID-19, a pilot study. Adv Sci. (2020) 7:e2001435. doi: 10.1002/advs.202001435, PMID: 35403380 PMC7404576

[10] Liu Y Yan L-M Wan L Xiang T-X Le A Liu J-M. Viral dynamics in mild and severe cases of COVID-19. Lancet Infect Dis. (2020) 20:656–7. doi: 10.1016/S1473-3099(20)30232-2, PMID: 32199493 PMC7158902

[11] Magleby R Westblade LF Trzebucki A Simon MS Rajan M Park J. Impact of SARS-CoV-2 viral load on risk of intubation and mortality among hospitalized patients with coronavirus disease 2019. Clin Infect Dis. (2020) 73:e4197–205. doi: 10.1093/cid/ciaa851 PMC7337625 32603425

[12] Fajnzylber J Regan J Coxen K Corry H Wong C Rosenthal A. SARS-CoV-2 viral load is associated with increased disease severity and mortality. Nat Commun. (2020) 11:5493. doi: 10.1038/s41467-020-19057-5, PMID: 33127906 PMC7603483

[13] Pujadas E Chaudhry F McBride R Richter F Zhao S Wajnberg A. SARS-CoV-2 viral load predicts COVID-19 mortality. Lancet Respir Med. (2020) 8:E70. doi: 10.1016/S2213-2600(20)30354-4, PMID: 32771081 PMC7836878

[14] World Health Organization. Laboratory testing for 2019 novel coronavirus (2019-nCoV) in suspected human cases. Available at: https://www.who.int/publications-detail/laboratory-testing-for-2019-novel-coronavirus-in-suspected-human-cases-20200117.

[15] Fodha I Vabret A Ghedira L Seboui H Chouchane S Dewar J. Respiratory syncytial virus infections in hospitalized infants: association between viral load, virus subgroup, and disease severity. J Med Virol. (2007) 79:1951–8. doi: 10.1002/jmv.21026, PMID: 17935185

[16] Zheng S Fan J Yu F Feng B Lou B Zou Q. Viral load dynamics and disease severity in patients infected with SARS-CoV-2 in Zhejiang province, China, January–march 2020: retrospective cohort study. BMJ. (2020) 2020:369m1443. doi: 10.1136/bmj.m1443 PMC7190077 32317267

[17] Pan Y Zhang D Yang P Poon LLM Wang Q. Viral load of SARS-CoV-2 in clinical samples. Lancet Infect Dis. (2020) 20:411–2. doi: 10.1016/S1473-3099(20)30113-4, PMID: 32105638 PMC7128099

[18] Yu F Yan L Wang N Yang S Wang L Tang Y. Quantitative detection and viral load analysis of SARS-CoV-2 in infected patients. Clin Infect Dis. (2020) 71:793–8. doi: 10.1093/cid/ciaa345, PMID: 32221523 PMC7184442

[19] da Silva RM Gebe Abreu Cabral P de Souza SB Arruda RF Cabral SPF de Assis ALEM. Serial viral load analysis by DDPCR to evaluate FNC efficacy and safety in the treatment of mild cases of COVID-19. Front Med. (2023) 10:1143485. doi: 10.3389/fmed.2023.1143485, PMID: 37007788 PMC10053779

[20] Winichakoon P Chaiwarith R Liwsrisakun C Salee P Goonna A Limsukon A. Negative nasopharyngeal and oropharyngeal swab does not rule out COVID-19. J Clin Microbiol. (2020) 58:e00297-20. doi: 10.1128/JCM.00297-20, PMID: 32102856 PMC7180262

[21] Ackley TW McManus D Topal JE Cicali B Shah S. A valid warning or clinical Lore: an evaluation of safety outcomes of Remdesivir in patients with impaired renal function from a multicenter matched cohort. Antimicrob Agents Chemother. (2020) 65:e02290–20. doi: 10.1128/aac.02290-20 PMC7849020 33229428

[22] Huang JT Liu YJ Wang J Xu ZG Yang Y Shen F. Next generation digital PCR measurement of hepatitis B virus copy number in formalin-fixed paraffin-embedded hepatocellular carcinoma tissue. Clin Chem. (2015) 61:290–6. doi: 10.1373/clinchem.2014.230227, PMID: 25361948

[23] Gupta RK Abdul-Jawad S McCoy LE Mok HP Peppa D Salgado M. HIV-1 remission following CCR5Δ32/Δ32 haematopoietic stem-cell transplantation. Nature. (2019) 568:244–8. doi: 10.1038/s41586-019-1027-4, PMID: 30836379 PMC7275870

[24] Ahmad SU Kiani BH Abrar M Jan Z Zafar I Ali Y. A comprehensive genomic study, mutation screening, phylogenetic and statistical analysis of SARS-CoV-2 and its variant omicron among different countries. J Infect Public Health. (2022) 15:878–91. doi: 10.1016/j.jiph.2022.07.002 35839568 PMC9262654

