# Supplementary material for: Phase III, randomized, double-blind, placebo-controlled clinical study: a study on the safety and clinical efficacy of AZVUDINE in moderate COVID-19 patients
Source: Front Med (Lausanne). 2023 Oct 19;10:1215916. doi: 10.3389/fmed.2023.1215916 (PMC10620601; doi:10.3389/fmed.2023.1215916)
Supplement: Supplementary file 1 [file Data_Sheet_1.PDF]

**Title of the research project**	**EVALUATION OF THE SAFETY AND CLINICAL EFFICACY OF AZVUDINE IN PATIENTS INFECTED WITH SARS-COV-2 VIRUS: A PHASE III, RANDOMIZED, DOUBLE-BLIND, PLACEBO-CONTROLLED STUDY.**

**Project location**	Santa Casa de Misericórdia de Campos, Campos dos Goytacazes-RJ.
**Internal protocol number**	IGZ-1
**Responsible Researcher**	Cleber Glória Silva
**Sponsor**	HRH HOLDINGS LIMITED represented by GALZU INSTITUTE for research, teaching, applied science and technology (ORPC - Representative Organization for Clinical Research).

CONFIDENTIALITY AND COMPLIANCE

This protocol was prepared and will be conducted in accordance with the standards established by the ICH Guidelines for Good Clinical Practice GCP/ICH - ICH *Topic* E6 (R2) (2016), Americas Document (2005), Resolution 466/12 of the National Health Council and related, RDC 09/15 of ANVISA and related and other applicable regulatory requirements. The data obtained will be used only for purposes determined in this research, keeping the confidentiality of the data and results obtained ensured by controlled access of people responsible for its evaluation and execution.

This document contains trade secret information that is privileged or confidential and should not be disclosed unless such disclosure is required by official or regulatory bodies. This protocol may not be modified or used without the consent of the **GALZU INSTITUTE of research, teaching, science and applied technology**.

**Sponsor**	**HRH HOLDINGS LIMITED represented by GALZU INSTITUTE for research, teaching, applied science and technology (ORPC - Representative Organization for Clinical Research).**

REVISION HISTORY

Version	Description	Date
0.1	Version approved by the Investigator and Sponsor	14/09/2020
0.2	Version approved by the Investigator and Sponsor	19/01/2021
0.3	Version approved by the Investigator and Sponsor	23/02/2021
0.4	Version approved by the Investigator and Sponsor	05/04/2021
0.5	Version approved by the Investigator and Sponsor	30/05/2022

SUBSCRIPTION PAGE

The signatures of the Principal Investigator and the Sponsor below constitute an agreement on this protocol and provide the necessary assurances that this study will be conducted by all specifications described in applicable laws and regulations and with the ethical principles described in the Declaration of Helsinki, Americas Document (2005), GCP/ICH Good Clinical Practice - ICH Topic E6 (R2) (2016), and compliance with Resolutions No. 466/12 of the National Health Council - Ministry of Health and related, RDC No. 09/15 of ANVISA and related, as well as other applicable regulatory requirements.

The Principal Investigator and Sponsor are aware that implementation of the trial protocol and documents can only be done after all necessary ethical and regulatory approvals have been obtained and the Sponsor has authorized the initiation of the trial.

Rio de Janeiro, May 30, 2022

Dr. Cleber Glória Silva
Diretor Geral
SCMC
CRM: 5276044-7

Dr. Cleber Glória Silva	Prof. Dr. Paula Gebe Abreu Cabral
Investigador Principal	Presidente / CPF: 859.888.127-91
CPF: 098.611.757-92	Instituto GALZU / ORPC de HRH HOLDINGS LIMITED

LIST OF ABBREVIATIONS

**Ab**	*Antibody*
**Ag**	Antigen
**Alb**	Albumin
**ALP**	Alkaline phosphatase
**ALT**	Alanine Aminotransferase (TGP)
**AST**	Aspartate Aminotransferase (TGO)
**BASO #**	Number of Basophils
**BI**	Researcher's brochure
**BNP**	B-type natriuretic peptide
**BPC**	Good Clinical Practice
**CHE**	Cholinesterases
**CIMS**	*Independent Data Monitoring* and Security Committee
**CK**	Creatine kinase
**Cl**	Chlorine
**COVID-19**	Corona Virus Disease 2019
**CRP**	C-Reactive Protein
**DBIL**	Direct Bilirubin
**DM**	*Data Manager*
**DMP**	*Data Management Plan*
**DNA**	Deoxyribonucleic Acid
**EA**	Adverse Event

**EAG**	Serious Adverse Event
**ECA2**	Angiotensin-converting enzyme 2
**ECG**	Electrocardiogram
**eCRF**	*Electronic Case Report Form*
**EFV**	Efavirenz
**EOS #**	Number of Eosinophils
**ESR**	Red Cell Sedimentation
**FAS**	*Full* Analysis *Set*
**FDA**	*Food and Drug Administration*
**FIB**	Fribrinogen
**FR**	Respiratory Rate
**GGT**	Gamma-Glutamyl Transferase
**Hb**	Hemoglobin
**HCT**	Hematocrit
**HIV**	Acquired Immunodeficiency Syndrome
**ICH**	*International Council for Harmonization of Technical Requirements for Pharmaceuticals for Human Use*
**IgA**	Immunoglobulin A
**IgG**	Immunoglobulin G
**IgM**	Immunoglobulin M
**IL-6**	Interleukin-6
**IWRS**	*Interactive Web Response* System

**K**	Potassium
**LYMPH#**	Number of Lymphocytes
**MCH**	*Mean* Corpuscular *Hemoglobin*
**MCHC**	*Mean Corpuscular Hemoglobin Concentration*
**MCV**	*Mean* Corpuscular Volume
**MONO #**	Number of Monocytes
**At**	Sodium
**NEUT#**	Number of Neutrophils
**NMPA**	*National Drug Administration*
**NOAEL**	*No Observed Adverse Effect* Level
**WHO**	World Health Organization
**WFP**	Mean blood pressure
**PaO2**	Arterial oxygen pressure
**PAS**	Systolic blood pressure
**PCT**	Procalcitonin
**PI**	*Principal* Investigator
**PLT**	Platelet count
**POP**	Standard Operating Procedure
**PPS**	*Per* Protocol Set
**RBC**	*Red blood cell* count
**RNA**	Ribonucleic acid
**SatO2**	Oxygen Saturation

**SCR**	Serum Creatinine
**SS**	Safety *Data Set*
**SUSAR**	*Suspected Unexpected Serious* Adverse *Reaction*
**TBIL**	Total bilirubin
**TC**	Computed Tomography
**TCLE**	Informed Consent Form
**TDF**	Tenofovir
**TMPRSS2**	Transmembrane serine protease 2
**TP**	Total Proteins
**TP**	Prothrombin time
**TT**	Thrombin Time
**TTPA**	Activated Partial Thromboplastin Time
**UA**	Uric Acid
**UREA**	Urea
**VM**	Mechanical Ventilation
**WBC**	*White Blood Cell* Count

LIST OF TABLES

Table 1: Chemical properties of AZVUDINE 33

Table 2: CL-236 (active metabolite of AZVUDINE) inhibits coronavirus activity 35

Table 3: Results of the Phase I multidose assay 37

Table 4: WHO Clinical Progression Scale (Jun/2020) 52

Table 5: Study groups 53

Table 6: Relationship between dose and administration time 59

Table 7: Relationship between dose and administration time 77

Table 8: Drug washout period 79

Table 9: Parameters for safety and efficacy evaluation 82

Table 10: Schedule of study visits and procedures. 93

Table 11: Causality relationship between AEs and study drugs. 101

LIST OF FIGURES

Figure 1: General flowchart of the study 25

Figure 2: Graph showing the EC50 of CL-236 35

Figure 3: Kaplan-Meier curves of two consecutive negative tests for SARS-CoV-2 (a) first and b) second) in FNC and control group. Data are percent (%). Differences between groups were using the Log-rank test (Mantel-Cox) 40

Figure 5: Mechanism of action of AZVUDINE a) Azvudine nucleoside-based anti-HIV clinical candidate (FNC) for the treatment of COVID-19. Structure of SARS-COV-2 RNA showing a cytosine subunit; b) Diagram showing the RNA replication process of SARS-COV-2 blocked by FNC; c) Proposed mechanisms of FNC to inhibit SARS-COV-2 activity 50

Figure 6: Macroregion of Norte Fluminense 60

Figure 7: Clinical research drug label 73

Summary

CONFIDENTIALITY AND COMPLIANCE 2
REVISION HISTORY 3
LIST OF ABBREVIATIONS 5
LIST OF TABLES 9
LIST OF FIGURES 10
1. SUMMARY 14
1.1. SYNOPSIS 14
1.2. FLOWCHART 25
1.3. STUDY SCHEDULE 26
2. INTRODUCTION 27
2.1. COVID-19 27
2.2. AZVUDINE 32
2.3. PRE-CLINICAL STUDIES 34
2.4. PHASE I AND II CLINICAL STUDIES 36
2.5. CLINICAL STUDIES WITH COVID-19 39
2.6. RISKS AND BENEFITS 41
2.6.1. Risks 41
2.6.2. Benefits 42
2.6.3. Analysis of risks and benefits 43
2.7. JUSTIFICATION 45
3. HYPOTHESIS 46
4. OBJECTIVES AND OUTCOMES OF THE STUDY 46
4.1. JUSTIFICATION OF PRIMARY OUTCOMES 50
5.1. STUDY DESIGN 52
5.1.1. Design 52
5.1.2. Justification for the choice of design 55
5.1.3. Justification for dose choice 57
5.1.4. Research location 59
5.2. STUDY POPULATION 61
5.2.1. Recruitment and Selection 61
5.2.2. Screening 64
5.2.3. Inclusion Criteria 64
5.2.4. Exclusion Criteria 65
5.3. VIROLOGICAL DIAGNOSIS 66
5.3.1. Diagnostic criteria for SARS- CoV-2 infection 67
5.3.2. Process standardization at proof of concept 67

5.4. MEASURES TO MINIMIZE STUDY BIAS 70
5.4.2. Blinding 70
5.4.3. Breaking the emergency blinding 70
6. TRATAMENT OF PARTICIPANTS 71
6.1. STANDART TREATMENT GIVEN IN THE CARE OF PATIENTS WHIT COVID-19 71
6.2. EXPERIMENTAL TRATAMENT 72
6.2.2. Packaging and labeling 73
6.2.3. Storage and Stability 74
6.2.4. Dose and method of administration 74
6.3.1. Overdosage 76
6.4. DRUG MANAGEMENT AND RECOVERY 77
6.5. DRUG INTERACTIONS 78
6.6. CONCOMITTANT THERAPIES AND PROHIBITED MEDICATIONS 78
7. EVALUATIONS AND VISITATION SCHEDULE 80
7.1. ASSESSMENT OF EFFECTIVENESS 82
7.1.2. Secondary Efficacy Evaluation 82
7.2. SAFETY ASSESSMENT 85
7.3. VISIT SCHEDULE 86
VISIT 1: INTIAL (D-1) 86
VISIT 2: RANDOMIZATION (D0) 87
VISIT 3-16: PROCEDURE (D1-D14) 87
VISIT 18: FOLLOW-UP (D21) 89
VISIT 19: FOLLOW-UP (D28) 90
VISIT 20: FOLLOW-UP (D60) 90
7.4. EXTRA VISITS 94
7.5. VIEWING WINDOW 94
8. ADVERSE EVENT REPORTING 94
8.1. SERIOUS ADVERSE EVENT (EAG) 95
8.2. SUSPECTED UNEXPECTED SERIOUS ADVERSE EVENT (SUSAR) 97
8.3. ADVERSE EVENT OF SPECIAL INTEREST 97
8.4. EAS MONITORING 98
8.5. EA INTENSITY 99
8.6. CASUALITY BETWEEN EAS AND DRUGS IN THE STUDY 99
8.7. AE COLLECTION AND REGISTRATION 101
8.8. EVALUATION OF ABNORMALITIES OF CLINICAL LABORATORY TESTS AND OTHER ABNORMALITIES CONSIDERED AS EA 103
8.9. PREGNANCY 103
8.10. NOTIFICATION OF AE 104

9. CONCLUSION AND DISCONTINUATION OF THE STUDY 104
9.1. CONCLUSION OF THE PARTICIPANT 104
9.2. STUDY CONCLUSION 105
9.3. INTERRUPTION OR DISCONTINUATION OF THE STUDY PARTICIPANT 105
9.4. DISCONTINUATION OR INTERRUPTION OF STUDY MEDICATION 106
9.5. LOSS OF FOLLOW-UP 107
9.6. EARLY STOPPING OR DISCONTINUING THE STUDY 108
10. DATA MANAGEMENT 109
10.3. MEDICAL CODING 111
10.4. DATA COLLECTION 111
10.5. PARTICIPANT ID 112
10.6. SOURCE DOCUMENTS 112
10.7. DATA COLLECTION FORM 112
10.9. AMENDMENTS TO THE PROTOCOL 114
10.10. CONFIDENTIALITY AND DATA PROTECTION 114
10.11. MONITORING 115
10.12. AUDIT 115
10.13. QUALITY CONTROL AND ASSURANCE 116
10.14. DATA ARCHIVING AND STORAGE 117
11. STATISTICAL ANALYSIS 117
11.2. STATISTICAL ANALYSIS POPULATION 121
11.3. METHOD OF STATISTICAL ANALYSIS 122
11.4. MODEL AND OPERATING CHARACTERISTICS 129
12.1. REGULATORY CONSIDERATIONS 130
12.2. INDEPENDENT DATA AND SECURITY MONITORING COMMITTEE (CIMS) 130
12.3. ETHICAL CONSIDERATIONS AND RESEARCH ETHICS COMMITTEE (CEP) 131
12.4. PROCESS FOR OBTAINING THE INFORMED CONSENT FORM (TCLE) 131
12.5. REIMBURSEMENT 132
12.6. PARTICIPANT ASSISTANCE 133
12.7. CONFIDENTIALITY AND DISSEMINATION OF RESEARCH RESULTS 133
12.8. RESPONSIBILITY OF THE PRINCIPAL INVESTIGATOR 134
12.9. RESPONSIBLE FOR THE AZVUDINE STUDY 135
12.10. DEADLINE FOR COMPLETION OF THE STUDY 136
13. REFERENCES 136
13.1. ANNEX 1 –Treatment Protocol for COVID-19 - 2nd Version (July 02, 2020) 140
13.3. ANNEX 3 – CIMS Internal Regulation - V2.0_19.01.2021 140
13.5. ANNEX 5 - Hope-4 Partial Data Report on the Efficacy and Safety of AZVUDINE 140

1. SUMMARY

1.1. SYNOPSIS

**TITLE**	Evaluation of the Safety and Clinical Efficacy of AZVUDINE in patients infected with SARS-CoV-2 virus: a Phase III, randomized, double-blind, PLACEBO-controlled study.
**STUDY ID**	IGZ-1
**DOCUMENT TYPE**	Phase III Clinical Trial Design
**VERSION/ISSUANCE DATE**	Version 0.5 of May 30, 2022
**SPONSOR**	HRH HOLDINGS LIMITED, HONG KONG / 2339920, 2502, NORTH TOWER, ZHONGZHOU CENTER, PAZHOU, HAIZHU DISTRICT, GUANGZHOU CITY, GUANGDONG, CHINA.
**REPRESENTATIVE ORGANIZATION OF CLINICAL RESEARCH SPONSOR IN BRAZIL (ORPC)**	INSTITUTO GALZU DE PESQUISA, ENSINO, CIÊNCIA E TECNOLOGIA APLICADA, CNPJ: 28.831.817/0001-20.<br>Office: Av. Lourival Martins Beda, 1082 - Donana, Zip Code: 28110-000, Campos dos Goytacazes-RJ.<br>Interface Hospitalar: Av. Pelinca, 115 - Parque Tamandaré, 3º andar, CEP: 28035-053, Hospital Santa Casa de Misericórdia de Campos, Phone: (22) 2726 6550 Ext. 180/194<br>Contact Paula Cabral (22) 98126 6602<br>Site: http://www.institutogalzu.org.br<br>E-mail: institutogalzu@galzu.org.br
**STUDY SITE**	Santa Casa de Misericórdia de Campos, Campos dos Goytacazes - RJ

**OBJECTIVE**	**Primary Goal**<br>• To evaluate the **efficacy and safety of** AZVUDINE (FNC) versus placebo in patients infected with moderate to severe stage SARS-COV-2;<br>**Secondary objective**<br>• To evaluate the **clinical outcome** between the AZVUDINE (FNC) group versus the PLACEBO group in patients infected with moderate to severe stage SARS-COV-2;
**INTERVENTION**	• Experimental group: AZVUDINE 5mg/day, orally for up to 14 days + Standard treatment.<br>• Control group: PLACEBO orally for up to 14 days + Standard treatment.
**NUMBER OF PARTICIPANTS**	Following initial study data, the sample size calculation was revised updating parameters of frequency of clinical improvement and dropout rate. The revised sample size is 180 participants randomly and equally divided into the two arms of the study (90 practitioners in each group).

**STUDY DESIGN**	The study consists of 3 periods: **screening, treatment, and follow-up.** In the screening period, patients who meet the eligibility criteria will be enrolled in the study and randomized into one of the two study arms, experimental or control. The first day of treatment will be day 1, and will last up to 14 days.<br><br>**Treatment will be terminated early when two consecutive RT-PCR tests are negative (one of the criteria for hospital discharge).** Treatment will also be stopped early in the event of participant dropout or in the event of an EAG.

	Safety follow-up will be performed at 28 and 60 days after discharge.<br>For the **Early Termination (ET)** of treatment, participants should follow the routine from visit 17: End of Treatment (D15), in advance.<br>The study will be considered completed when the last participant completes his or her last visit.
**INCLUSION CRITERIA**	Individuals with the following characteristics will be included in this study:<br>1. Age ≥18 years, regardless of gender;<br>2. Patients hospitalized in moderate to severe stages in line with the Ministry of Health classification[1] ;<br>3. Positive diagnostic test for SARS-CoV-2 by molecular amplification of the virus in RT-PCR diagnosed from respiratory specimen (nasopharyngeal, oropharyngeal, lower respiratory tract [e.g., sputum]) collected <96 hours before randomization;<br>4. Time between symptom onset and inclusion ≤14 days;<br>5. Hospitalization within 48 hours of study inclusion;<br>6. Availability for follow-up during the study period;

[1] The Brazilian Ministry of Health classifies the symptoms of COVID-19 into:
Mild: Flu-like syndrome (cough, sore throat or runny nose), followed or not by: Anosmia (olfactory dysfunction) Ageusia (gustatory dysfunction), Runny nose, Diarrhea, Abdominal pain, Fever, Chills, Myalgia, Fatigue, Headache;
Moderate: Persistent cough + daily persistent fever or Persistent cough + progressive worsening of another symptom related to COVID-19 (adynamia, prostration, hyporexia, diarrhea) or at least one of the above symptoms + presence of a risk factor.
Severe: Severe acute respiratory syndrome - influenza syndrome presenting: Dyspnea/respiratory distress or persistent chest pressure or O_2 saturation less than 95% on room air or bluish coloration of lips or face.

	7. Voluntary adherence to participate in the study and signing the Informed Consent Form.
**EXCLUSION CRITERIA**	Individuals with one or more of the following characteristics will not be eligible to participate in this study:<br>1. Patients known or suspected to be sensitive to AZVUDINE or excipients (inactive ingredients: microcrystalline cellulose, hydrated lactose, polyvinylpyrrolidone K30, croscarmellose sodium, magnesium stearate);<br>2. Patients diagnosed with pneumonia caused by other pathogens;<br>3. Patients with liver disease (total bilirubin ≥2mg/dL, ALT/TGP and AST/TGO ≥5 times above normal limit); [2]<br>4. Total neutrophil count <750 cells/L;<br>5. Patients with renal insufficiency (glomerular filtration rate ≤60mL/min/1.73m2) or are receiving continuous renal replacement therapy, hemodialysis or peritoneal dialysis;<br>6. Patients with congestive heart failure (NYHA grade 3/4), untreated symptomatic arrhythmias, or myocardial infarction within 6 months;<br>7. Patients with a history of known liver disease (cirrhosis with *ChildPugh* classification B and C);<br>8. Individuals with malabsorption syndrome, or other conditions that affect gastrointestinal absorption, and

[2] Normal values: BT up to 1.2 mg/dL; TGO Adult 12 to 46 U/L; TGP Adult 03 to 50 U/L; GGT Man 10 to 50 U/L, Woman 07 to 32 U/L (Source: Hermes Pardini Institute).

	circumstances in which patients need intravenous nutrition, or cannot take medications orally or nasogastrically;<br>9. Women who are pregnant or lactating, or of childbearing potential during the study period and within 6 months after termination of administration;<br>10. Patients already included in other clinical trials;<br>11. Patient under treatment for HIV, Hepatitis C, active Hepatitis B;<br>12. Patients being treated with other antivirals (e.g. lopinavir/ritronavir, remdesivir, umifenovir/arbidol, favipiravir, interferon-α)<br>13. Patients being treated with monoclonal antibodies (e.g. tocilizumab and sarilumab/kevzara);<br>14. Patients who are on a clinical treatment plan that includes concomitant administration of any other **experimental treatment** or ***off-label*** use of already marketed drugs (e.g. hydroxychloroquine sulfate);<br>15. Patients requiring invasive mechanical ventilation or extracorporeal membrane oxygenation (ECMO) at the time of randomization;<br>16. Any clinically significant medical condition or medical history that, in the investigator's opinion, may discourage participation in the study, such as corrected QT interval >480 on the electrocardiogram, among other conditions.

**STUDY INTERRUPTION CRITERIA**	The following criteria define **study termination for the participant**:<br>1) Voluntary withdrawal from the participant at any time and without any prejudice to his/her health;<br>2) Any situation, at the discretion of the Principal Investigator, that puts the safety of the participant at risk;<br>3) Refusal or inability to perform and/or complete imaging and laboratory tests, when applicable;<br>4) Loss of contact with the participant for follow-up;<br>5) Death;<br>6) Hospital discharge (2 negative RT-PCR tests).<br>The following criteria define the **interruption of thestudy** as a whole:<br>1) Termination of the trial by the sponsor, if justified;<br>2) Termination of the study by the Principal Investigator, if justified;<br>3) Termination of the study at the request of local ethical and health authorities.
**PROCEDURES**	Demographic data collection, physical examination, vital signs assessment, blood sampling, respiratory tract sampling, imaging, ECG, and pregnancy testing (female participants) will be performed
**OUTCOME/ENDPOINTS**	**PRIMARY ENDPOINT**

	• Proportion of participants with clinical status improvement [Period: until D15, approximately 14 days].<br>○ The criterion for a participant to have an improvement in clinical status is a decrease on the WHO Clinical Improvement Ordinal Scale in at least one category compared to screening (WHO, Jun/2020).<br><br>**SECONDARY ENDPOINT**<br><br>• Proportion of participants with a clinical outcome of cure during the study; [Period: until D15, D21, and D28, approximately 14-30 days].<br>○ The clinical endpoint of cure is defined in this protocol as the absence of viral RNA in collected samples and clinical conditions for hospital discharge[3] .<br>• Time to normalization of body temperature (below 37.6°C axillary); [Period: until D15, D21 and D28, approximately 14-30 days]<br>• Time to improvement of diarrhea, myalgia, fatigue, malaise, cough, dyspnea, and headache; [Period: until D15, D21, and D28, approximately 14-30 days].

[3] According to the WHO Working Group on Characterization and Clinical Management of COVID-19 Infection (July, 2020), clinical and virological absence of infection (non-detectable RT-PCR) is suggestive of CURE for initially infected participants.

	• Changes in liver and kidney function *baselines*, and inflammatory and immunological markers: [Period: until D15, D21 and D28, approximately 14-30 days]<br>○ Lab tests performed on days D1, D3, D5, D7, D9, D13, D15, D21, D28, and D60.<br>• Evaluation of the time to negative conversion of the SARS-CoV-2 viral load by RT-PCR between AZVUDINE (FNC) group and control group; [Period: until D15, D21 and D28, approximately 14-30 days].<br>• Evaluation of the number of cycles for the detection of SARS-CoV-2 viral load by RT-PCR and application of the standard curve for viral load calculation; [Period: until D15, D21 and D28, approximately 14-30 days].<br>• Analysis of the relationship between the calculated viral load and the clinical progress of participants in the AZVUDINE (FNC) group compared to the control group; [Period: until D15, D21 and D28, approximately 14-30 days].<br>• Time and proportion of lung imaging improvement during treatment (Pattern of ground-glass opacities, mosaic paving, alveolar consolidation, reticular pattern/septal thickening, opacity with inverted halo, pleural/pericardial effusion, fibrosis and/or lymphadenomegaly); [Period: until D15, D21 and D28, approximately 14-30 days].<br>• Time of improvement and proportion of respiratory symptoms and signs during treatment (Lung rales,

	cough, sputum, sore throat); [Period: until D15, D21, and D28, approximately 14-30 days].<br>• Time to normalization of O_2 saturation (above 95%); [Period: until D15, D21 and D28, approximately 14-30 days].<br>• Time to normalization of respiratory rate to levels ≤ 24rpm, in room air; [Period: until D15, D21 and D28, approximately 14-30 days]<br>• Frequency of supplemental oxygenation or non-invasive ventilation; [Period: until D15, D21 and D28, approximately 14-30 days].<br>• Frequency of invasive mechanical ventilation or extracorporeal membrane oxygenation (ECMO); [Period: until D15, D21 and D28, approximately 14-30 days].<br>• Proportion of moderate cases that progressed to severe cases requiring intensive care unit (ICU) care; [Period: until D15, D21, and D28, approximately 14-30 days].<br>• Length of hospital stay; [Period: until D15, D21, and D28, approximately 14-30 days].<br>• Occurrence of drug interactions; [Period: until D15, D21 and D28, approximately 14-30 days].<br>• Frequency and intensity of adverse events; [Period: until D15, D21 and D28, approximately 14-30 days].

	• Frequency and intensity of unexpected adverse events; [Period: until D15, D21 and D28, approximately 14-30 days].<br>• Frequency and intensity of serious adverse events; [Period: until D15, D21 and D28, approximately 14-30 days].<br>• All-cause mortality rate during the study; [Period: until D15, D21 and D28, approximately 14-30 days].<br>• Evaluate the tolerability of AZVUDINE (FNC) use at 5mg/day regimen for up to 14 days: [Period: until D15, approximately 14 days].<br>○ Calculating treatment adherence;<br>○ Total time of use of AZVUDINE (FNC);<br>○ Proportion of participants who withdrew consent;<br>○ Proportion of participants who dropped out of treatment.
**ANALYSES**	All statistical tests are performed by two-sided test. A significance level of 5% will be adopted. Detailed statistical methods will be provided in chapter 11.
**LENGTH OF STUDY**	From the approval of the study by the Research Ethics Committee and ANVISA, it is estimated to take approximately 7 months to complete.

1.2. FLOWCHART

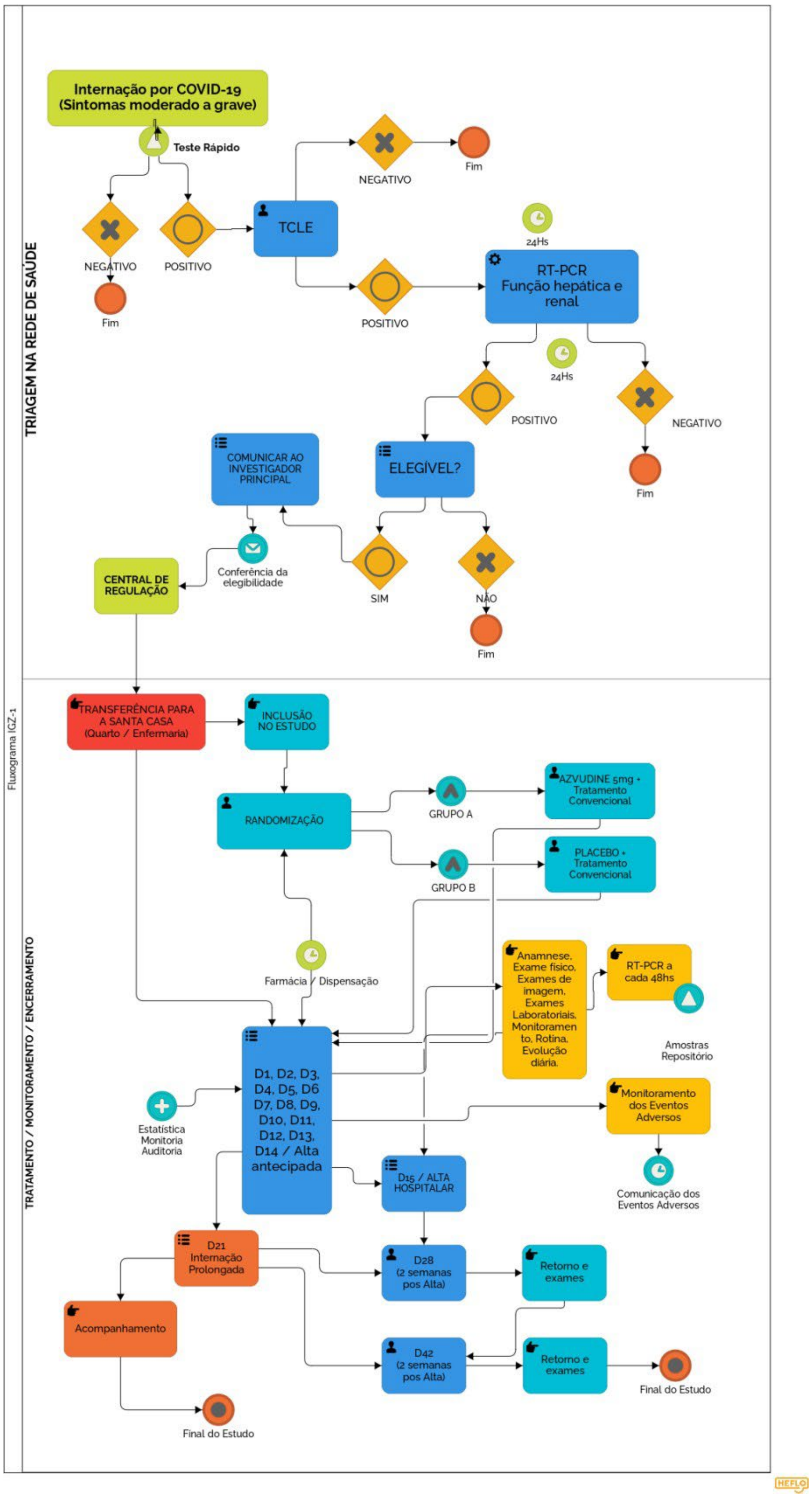

Figure 1: General flowchart of the study

1.3. STUDY SCHEDULE

The research project will follow the execution schedule, which comprises a total period of 7 months, counting from the proof of registration of the research in Plataforma Brasil and release for execution by the CEP/CONEP system. Being aware of the deadlines established for ethical analysis by the CEP/CONEP system, as set forth in the Operational Rule 001/2013 of the National Health Council and ANVISA Resolutions, the following schedule is proposed:

<table>
<tr><th colspan="2">Steps</th><th>Description</th><th>Expected period</th></tr>
<tr><td rowspan="4">ELABORATION</td><td>Preparation of the project and other necessary documents</td><td>Preparation of the research project, brochure, TCLE, ethical and regulatory dossier</td><td>July to September 2020</td></tr>
<tr><td rowspan="2">Regulatory Approvals</td><td>Submission of the dossier for ethical evaluation by the CEP/CONEP System</td><td>September and October 2020</td></tr>
<tr><td>Submission of the DDCM for regulatory evaluation by ANVISA</td><td>October and November 2020</td></tr>
<tr><td colspan="3">The beginning of the activities of the Research Project will only occur after the approval of the CEP, reason why it was not possible to estimate a period, but the time necessary for the conduction of each step</td></tr>
<tr><td rowspan="3">CONDUCTION</td><td>Recruiting</td><td>Outreach and recruitment of research participants</td><td>April 2021</td></tr>
<tr><td>Screening</td><td>Invitation to Participant
Application and signing of the TCLE
Eligibility Criteria</td><td>April to July 2021</td></tr>
<tr><td>Data treatment and collection</td><td>Treatment of research participants with an investigational drug or PLACEBO, concomitant with standard treatment for COVID-19</td><td>April 2021 to August 2021</td></tr>
<tr><td rowspan="3">CONCLUSION</td><td>Results Evaluation and Statistical Analysis</td><td>Correlations between measured parameters and clinical data</td><td>September 2021</td></tr>
<tr><td>Final study report</td><td>Background and discussion of research findings</td><td>October 2021</td></tr>
<tr><td>Publication of results</td><td>Dissemination of results to the scientific community</td><td>November 2021</td></tr>
</table>

Please note that the dates may be altered depending on the deadlines for ethical and regulatory review. In case of approval before the schedule, the inclusion of participants and data collection will be anticipated.

2. INTRODUCTION

2.1. COVID-19

In December 2019 the world observed the emergence of unexplained cases of pneumonia in Wuhan, China. These cases were associated with a new virus from the *coronaviridae* family, identified in January as SARS-CoV-2, which causes the disease COVID-19 (*Corona Virus Disease* 2019) (VELAVAN; MEYER, 2020). According to the World Health Organization (WHO), transmission can occur through contact, breaths or droplets generated during coughing, sneezing or enunciation, which can remain in the air for long distances and time, particularly in a poorly ventilated environment, and which somehow come into contact with mucous membranes (WHO, 2020). The average incubation time of SARS-CoV-2 is around 5 days, ranging from 2 to 14 days (WHO, 2020d). The most common symptoms are fever, cough, runny nose, sore throat, shortness of breath, loss of smell, altered sense of taste, fatigue, and decreased appetite (WHO, 2020). The clinical picture of infected people can range from an asymptomatic state or common cold to a severe state of pneumonia, which can lead to respiratory failure and death (SINGHAL, 2020). According to the World Health Organization, the mild state is characterized by symptoms such as fever, cough, loss of smell, among others, but without evidence of pneumonia or hypoxia. The moderate stage is characterized by non-severe pneumonia, and the severe stage is characterized by severe pneumonia and severe respiratory distress. Patients may progress to a critical stage of the disease, which is characterized by worsening respiratory symptoms, sepsis, or septic shock, possibly leading to death (WHO, 2020a).

As of February 21, 2022, the Ministry of Health has registered 22,845,551 confirmed cases and 644,604 deaths, resulting in an incidence of 13,440.8 cases and mortality of 306.7 deaths (both per 100,000 population). Worldwide, the WHO has registered 423,437,674 cases and 5,878.328 deaths.

In Campos dos Goytacazes - RJ, until February 18, 2022, 56,485 confirmed and

suspected cases of COVID-19 were reported. Among them, 2,246 cases were of severe acute respiratory syndrome with hospitalization (ICU occupation - 61.73% in the SUS and private network and medical clinic occupation - 49.52% in the SUS and private network). The incidence of SARS-CoV-2 infection in Campos dos Goytacazes is 56,485 cases and a mortality rate of 1,809 deaths (both per 100,000 inhabitants - Prefeitura de Campos, 2022).

Coronaviruses have three surface proteins: envelope protein, membrane protein and the S-glycoprotein (Spike), the latter of which contributes to host receptor binding, cell tropism and pathogenesis. The S proteins form a trimer generating a crown-shaped structure, which explains the name of this virus family (*corona* = crown) and are functionally divided into the S1 and S2 domains. Upon entry into human cells, SARS-CoV-2 binds to the angiotensin-converting enzyme type 2 (ACE2) via the S1 domain with the aid of Transmembrane Serine Protease-2 (TMPRSS2), and membrane fusion is carried out by the S2 domain. Subsequently, the viral genomic RNA is released into the cytoplasm of infected cells. One of the first processing steps of the coronavirus is the cleavage of the pp1a and pp1ab polypeptides by a protease, forming a complex of proteins responsible for viral RNA replication and transcription. The pp1a protein complex is formed by viral RNA polymerase (RdRp), helicase and other functional enzymes related to viral RNA modification (Romano et al., 2020). After replication and sub genomic RNA synthesis, viral structural proteins are translated and inserted into the endoplasmic reticulum. The viral genomes are encapsulated by the viral proteins formingmature virions. After assembly, the virions are transported to the cell surface in vesiclesand released by exocytosis (Fehr & Perlman, 2015).

Possible targets of SARS-CoV-2 (where ACE2 is abundantly present) include the lining epithelium of the lungs, gastrointestinal system, heart, kidneys, and brain. A number of studies are exploring the immune response to SARS-CoV-2, with most suggesting that the immune response occurs by monocyte/macrophage activation, elevation of pro-inflammatory cytokines, depletion of lymphocytes, and deployment of

large numbers of neutrophils, but the mechanisms involved are not yet well understood (Abdullah & Sharquie, 2020; Domingo.*et al.*, 2020; Ezzikouri *et al.*, 2020; Zhand *et al.*, 2020)

Understanding the mechanism of coronavirus replication is essential for developing therapies. As the development of new drugs is a long and expensive process, the knowledge gained from the SARS-CoV and MERS-CoV outbreaks of the early 21st century has helped in the search for treatments against COVID-19, as several interventions cited in the past, with different modes of action, have been studied for the current pandemic. The *R&D Blueprint* group *at WHO has* published a document with an overview of experimental treatments for COVID-19, separating the medications according to their classifications (WHO, 2020c).

Some **antiparasitic drugs** have been considered as potential treatments for SARS-CoV-2, such as ivermectin, chloroquine, and hydroxychloroquine.

Ivermectin is one of the most important anti-parasitic drugs used in human and veterinary medicine. Some *in vitro* and *in vivo* studies (using animal models) indicate the efficacy of ivermectin in inhibiting viral replication from the inhibition of nuclear transport mediated by the α/β1 importin heterodimer (Wagstaff *et al.*, 2012). This inhibition appears to be critical for blocking the replication of different RNA viruses, such as HIV viruses (Wagstaff *et al.*, 2012), Dengue (Tay , *et al.*, 2013)Influenza (Götz *et al.*, 2016)and SARS-CoV-2 itself (Caly *et al.*, 2020). To date, this last-mentioned study is the only one evaluating the efficacy of ivermectin on SARS-CoV-2 replication *in vitro*. The results obtained indicated a significant decrease in the levels of isolated viral RNA (originating from the virus) and processed viral RNA (originating from viral replication in host cells) in SARS-CoV-2 infected cells after treatment with 5μM of ivermectin. Furthermore, treatment with different concentrations of ivermectin, in addition to generating a dose-dependent response (IC_{50} ~ 2μM), did not show significant toxicity levels. Such a result, although unique, encouraged several research groups to

immediately initiate clinical trials to evaluate the efficacy and safety of using ivermectin to treat patients with COVID-19 (NCT04422561 and NCT04343092). However, no results are available to date, which is why ANVISA and international bodies such as the FDA conclude that there is insufficient clinical evidence for the use of this treatment and do not recommend the use of ivermectin for the treatment of COVID-19.

Chloroquine is a drug used to treat malaria, lupus erythematosus, rheumatoid arthritis, liver amebiasis, and photosensitive skin rashes. Despite widespread speculation, recent data from large clinical trials have shown no treatment benefit against COVID-19, leading the FDA to withdraw the emergency use authorization issued in March for chloroquine and hydroxychloroquine as treatments for COVID-19.

Some **antivirals** have been raised as alternatives against SARS-CoV-2, such as lopinavir/ritonavir which is primarily used against HIV. Despite limited data to support the use in the treatment of COVID-19, there are an important number of clinical trials registered to test this co-formulation in the treatment against the new coronavirus. Also, remdesivir is a broad-spectrum antiviral that acts as an RNA-dependent RNA polymerase inhibitor, inhibiting virus replication, and is therefore an attractive target as a viral inhibitor and considered promising for a future COVID-19 solution. Umifenovir (arbidol) and favipiravir are also antivirals with ongoing studies for COVID-19. Camostat is a serine protease inhibitor approved in Japan for treatment of chronic pancreatitis due to its ability to inactivate trypsin. This medication has demonstrated inhibitory activity against TMPRSS2, and is therefore a potential treatment drug against COVID-19. Nafamostat is a chemical analog of camostat but has a very short half-life and may significantly impair clinical utility in SARS-CoV-2 infection.

Biological agents may be great allies in the fight against the new coronavirus. Tocilizumab and sarilumab (kevzara) are recombinant monoclonal antibodies against the IL-6 receptor used for the treatment of cytokine release syndrome, rheumatoid arthritis, and systemic juvenile idiopathic arthritis. Since patients with COVID-19 also have higher

plasma levels of cytokines (closely related to disease severity and prognosis), interfering with this may also be a possible treatment for severe and critical COVID-19 infections.

Convalescent plasma therapy is another possible alternative, but one that relies on the administration of plasma from recovered COVID-19 patients, and is more effective as prophylaxis than as treatment, with a better result when administration is done soon after the onset of symptoms.

As **adjuvant/supportive therapy**, there are studies with azithromycin, a macrolide antibiotic, which also has anti-inflammatory and immunomodulatory functions and may minimize the elevated cytokine production associated with pulmonary viral infections. Also, to aid cytokine production, corticosteroids may be useful in the treatment of severe COVID-19, but they must be used for a limited time and with a controlled dose (Chibber et al., 2020; Nitulescu et al., 2020; Zhand et al., 2020).

Despite several indications and ongoing studies, no therapy has been officially approved, to date, for the treatment of COVID-19. To establish a standard of management of patients with COVID-19, the Ministry of Health has published a document with guidelines. For **mild cases**, it is important to perform an anamnesis, physical examination, and complementary exams (as available) for a better approach and management. The doctor can proceed with the prescription at his discretion, according to the clinical diagnosis made, observing the local recommendations and the guidelines of the Ministry of Health. For **moderate cases** (who need hospitalization for observation and clinical follow-up, but do not meet the criteria for ICU admission, i.e., they have no organ dysfunction or hemodynamic instability and do not require mechanical ventilation or other intensive care procedures) hospitalization is recommended until clinical stabilization (absence of fever and dyspnea for at least 48 hours) and improvement in laboratory parameters. For **severe cases**, early therapy and monitoring should be adopted, administering supplemental oxygen immediately to patients with respiratory distress, hypoxemia, or shock with a target SpO2 of 92-96%.

Systemic corticosteroids should not be routinely administered to treat viral pneumonia or respiratory failure unless otherwise indicated, such as chronic use. Practitioners should be alert to recognize severe hypoxemic respiratory failure when a patient with respiratory distress is failing standard oxygen therapy and provide advanced oxygen/ventilation support. First, noninvasive ventilation should be attempted. According to the Brazilian Intensive Care Medicine Association, CNAF (high-flow nasal catheter) may be tried in dyspneic and hypoxemic patients. If there is no improvement with the use of CNAF, orotracheal intubation is indicated and invasive mechanical ventilation is started. If the patient remains hypoxemic, transfer to a reference hospital for extracorporeal membrane oxygenation should be considered (Ministry of Health, 2020c).

2.2. AZVUDINE

The **nucleoside analog** plays an important role in the treatment of antiviral infections. **RNA synthesis is an indispensable step in the coronavirus replication life cycle**, which is usually successfully accomplished **using natural nucleosides from the host cells as substrates. Nucleoside and nucleotide analog inhibitors are synthetic molecules analogous to purines and pyrimidines, which can be mistakenly incorporated during viral replication, terminating viral RNA chain synthesis or causing mutations that lead to loss of viral viability, preventing viral replication and viral spread** (Pruijssers & Denison**,** 2019).

Nucleoside analogues are successfully used to treat DNA and RNA viral infections, such as HIV, hepatitis B virus, hepatitis C virus, herpes virus, and various respiratory viruses, such as Influenza and the coronaviruses (Carroll & Olsen, 2006; Jordan et al., 2018).

The new coronavirus is a new type of RNA virus.

Several nucleoside analogues have been reported to show the effect on inhibiting coronavirus replication *in vitro*, such as ribarivine, faviparavir and remdesivir, which shows a strong effect on inhibiting coronavirus replication *in vitro*. The drugs mentioned above are in early stage of research and development, so their safety and clinical efficacy still needs to be explored (Choy et al., 2020; Drożdżal et al., 2020; M. Wang et al., 2020). Thus, it is necessary to find new nucleoside analogues for the treatment of COVID-19.

AZVUDINE (FNC) is a new type of nucleoside analog reverse transcriptase inhibitor, developed for the treatment of HIV.

Their chemical properties are shown in Table 1 below:

Chemical Name	**4-amino-1-((2R,3S,4R,5R)-5-azido-3-fluoro-4-hydroxy-5-(hydroxymethyl)tetrahydrofuran-2-yl)pyrimidin-2(1H)-one**
Chemical Structure	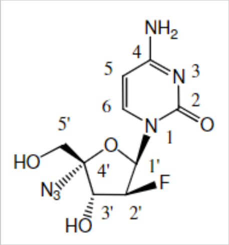
Molecular Formula	$C_9H_{11}FN_6O_4$
Molecular Weight	286.220g/mol
Properties	Clear yellow, odorless and tasteless crystal

Table 1: Chemical properties of AZVUDINE

AZVUDINE is a 4'-C(substituted)-2'-deoxynucleoside (4'sdN) with a 3'-OH group, which mimics the natural deoxynucleotide (dN) and has good stability in acid medium due to its 2′-fluoro substituent. The 4′sdNs retain all the functional groups of a dN, so

HIV replication complexes cannot discriminate between a 4'sdN and dN easily. Once the 4'sdN has been incorporated into the growing DNA chain, it is highly likely that immediate termination of the viral DNA biosynthesis chain occurs, blocking the addition of new dNs (Sun *et al.*, 2020). *In vitro* studies have shown good antiviral activity of AZVUDINE for both DNA viruses, such as hepatitis B, and RNA viruses, such as hepatitis C and SARS-CoV-2 (R.-R. Wang *et al.*, 2014).

Currently, phase I and II studies with AZVUDINE in HIV-positive volunteers have been completed in China, with good efficacy and safety, and the phase III study is ongoing, also in China. *In vitro* studies indicate that AZVUDINE has antiviral activity against coronaviruses, and clinical studies with volunteers with COVID-19, conducted in China, have shown that AZVUDINE has a significant therapeutic effect in the treatment of COVID-19. These data show that AZVUDINE has significant and broad-spectrum inhibition of RNA and DNA viruses, and its clinical safety is good. The clinical trial to evaluate the efficacy and safety of AZVUDINE has been approved by the *National Drug Administration* (NMPA) for the Phase III clinical study of anti-COVID-19, with approval number 2020L00015.

2.3. PRE-CLINICAL STUDIES

In vitro studies, conducted in China, demonstrated good activity and selectivity of AZVUDINE on cells infected with SARS-CoV-2. An *in vitro* activity study demonstrated that the active metabolite CL-236 of AZVUDINE has an antiviral activity against coronavirus. The EC50 is 1.51 µM and the selectivity index is 60 (Table 2: CL-236 (active metabolite of AZVUDINE) inhibits coronavirus activity). The test results support speculation of the mechanism of action of AZVUDINE against COVID-19.

Compound	EC_{50} (µM)	CC_{50} (µM)	SI
CL-236	1,51	89.94	60
Compound	EC_{50} (µM)	CC_{50} (µM)	SI
Ribavirin	17,9	>409	>23

Table 2: CL-236 (active metabolite of AZVUDINE) inhibits coronavirus activity

Another AZVUDINE *in vitro* test showed that the active metabolite of AZVUDINE, CL-236, has good antiviral activity against COVID-19 and its EC50 against SARS-CoV-2 is about 3.2 µM, which is equivalent to Remdesivir, the positive control drug.

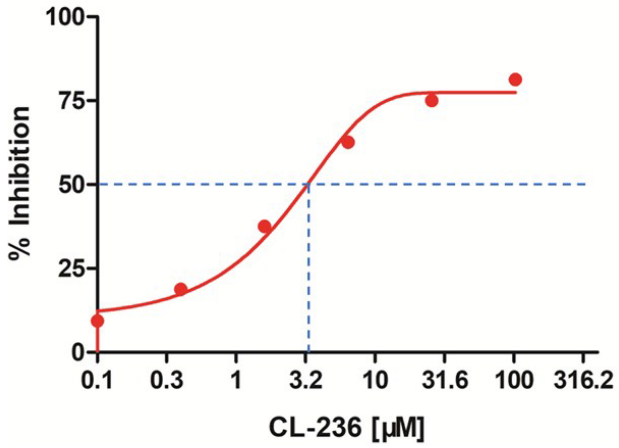

Figure 2: Graph showing the EC50 of CL-236

In vivo studies have demonstrated the safety of AZVUDINE. Administration of AZVUDINE in single doses of 0.24 mg/Kg, 0.48 mg/Kg or 0.96 mg/Kg had no significant effect on the circulation and respiratory system of Beagle dogs. The results of the *in vivo* toxicity test in Beagle dogs receiving AZVUDINE for 39 weeks showed that at high doses of 0.3 mg/kg, the main target organs are the gastrointestinal tract and the hematopoietic immune system. After 4 weeks of drug withdrawal, the toxic reaction can be basically restored. No toxicity reaction was observed in the 0.03 and 0.1 mg/kg dose groups. Systemic exposure to the drug has shown that the level of observable adverse events

(NOAEL) is equivalent to 20-30 times the human exposure at the maximum clinical dose used in phase II (4 mg).

Dosages of 1.2 and 2.4 mg/Kg were safe for the nervous system of mice and the dose of 4.8 mg/Kg reduced the sleep time of mice induced by pentobarbital sodium threshold. Sodium pentobarbital is a central depressant with sedative and hypnotic effects. When sodium pentobarbital is administered in threshold doses, drugs that could stimulate or inhibit the central nervous system can increase or decrease the sleep time of mice.

Like many other nucleoside analogues, some dose of AZVUDINE was positive in the genotoxicity test. However, doses more than 1000 times above the clinical dose caused neither gene mutations by the Ames Test nor chromosomal aberrations in CHL cells *in vitro*.

No effect of AZVUDINE was observed on mating or fertility, sperm and offspring of male rats. When administered 1.5 mg/kg AZVUDINE in pregnant rats during the perinatal period, there was slight toxicity demonstrated, but the offspring rats showed no obvious abnormalities. A small amount of AZVUDINE was detected in milk. The above non-toxic reaction dose is equivalent to 1-3 times the maximum clinical dose in Phase II.

The NOAEL of AZVUDINE for developing progenitor rabbits, embryos and fetuses is 1.0 mg/kg, which is equivalent to 5 times the maximum clinical dose in Phase II. Systemic exposure to the drug in rats and rabbits in this study was 50-100 times higher than in humans (the maximum clinical dose in Phase II).

2.4. PHASE I AND II CLINICAL STUDIES

Phase I clinical studies showed that there were no serious adverse reactions in the single-dose and multidose trials. Adverse events (AEs) in the single-dose trials with 4 mg of AZVUDINE were mainly fever, headache, dizziness, nausea, vomiting, and diarrhea, and the intensity was grade 1.

There were no serious adverse reactions in the multidose trial (GQ-FNC-2014-2), and after continuous administration of AZVUDINE 2 mg, 3 mg or 4 mg for 7 days, HIV-1 RNA viral load values were significantly reduced (Table 3: Results of the Phase I multidoseassay).

The adverse reactions were mainly dizziness and nausea, with grade 1 to 2 severity, and no additional treatment was required. No serious adverse reactions occurred in the drug interaction trials (GQ-FNC-103 and GC-FNC-104). The severity of adverse reactions in the AZVUDINE, TDF (tenofovir), AZVUDINE + TDF, EFV (efavirenz), and AZVUDINE + EFV groups were all grade 1 and no additional treatment was needed.

Group	Decrease value of viral load
2mg (QD)	1,17 – 2,43 (1,67 ±0,41)
3mg (QD)	0,67 – 1,42 (1,20 ±0,24)
4mg (QD)	0,45 – 2,05 (1,41 ±0,51)

Table 3: Results of the Phase I multidose assay

A randomized, multicenter, active-drug-controlled, dose-exploratory, phase II clinical study (GQ-FNC-201) was conducted to evaluate the safety and efficacy of AZVUDINE combined with the antivirals TDF and EFV in HIV-positive volunteers. A total of 172 subjects were included in the study, divided equally into 4 study arms, 3 experimental with doses of 2 mg, 3 mg, or 4 mg of AZVUDINE combined with TDF + EFV and a control with the active drug 3TC combined with TDF + EFV.A total of 172 subjects were included in the study, of which 155 subjects completed the 48 weeks, 15 subjects withdrew from the study due to drug resistance, poor adherence, voluntary substitution with self-pay drugs, AEs, etc. Only 2 individuals withdrew their informed consent forms.

Overall, the subjects had good tolerability and were highly adherent to the treatment regimen, and most of the adverse events that occurred were mild or moderate.

After treatment in each group, HIV-1 RNA decreased rapidly. The main indicators of efficacy were the percentage of subjects with HIV-1 RNA <50 copies/mL. The majority of participants' HIV-1 RNA dropped below the 50 copies/mL level in 12-24 weeks through 48 weeks. By FAS analysis, in approximately 90% of individuals, from each group, a rate of HIV-1 RNA <50 copies/mL was observed. By the PPS analysis, this result was observed in approximately 98% of participants in each group. For both analyses, the difference between the groups was not statistically significant (P >0.05). A chronological analysis showed that the percentage of subjects with HIV-1 RNA <50 copies/mL in the AZVUDINE 3mg group was slightly higher than at 12 and 24 weeks in the AZVUDINE 2mg, AZVUDINE 4mg, and 3TC groups, and the difference between the AZVUDINE 2mg and AZVUDINE 3mg groups was statistically significant (P <0.05). Among the three AZVUDINE dose groups, the rate of HIV-1 RNA decline in the AZVUDINE 3mg group was relatively faster than in other groups.

The three secondary efficacy evaluation indicators were the change in the logarithm of HIV-1 RNA before and after treatment, the number of subjects whose HIV-1 RNA level decreased by ≥1 log10 from baseline before and after treatment, and the change in CD4+ T-cell count before and after treatment. After 48 weeks of treatment, changes of approximately -3.2 were observed for each of the groups and there was no significant difference in the decrease in values across groups (P >0.05). The percentage of subjects whose virus RNA level decreased by ≥1 log10 was approximately 99%, for each of the groups, and there was no statistically significant difference between the groups (P >0.05). For CD4+ T-cell counts, a more pronounced trend toward an increased number of cells could be observed for the AZVUDINE 2mg group, but the analysis of covariance showed no significant difference between the groups (P >0.05).

AZVUDINE 2mg, 3mg and 4mg groups showed good safety. The total incidence of drug-related adverse events (85.7%, 92.9%, 93.0%, respectively) and the degrees of intensity were similar to those in the 3TC group (88.4%). The difference was not statistically significant (P >0.05). Among all adverse reactions, only 3 adverse reactions caused withdrawal in the AZVUDINE 3mg (2.4%), AZVUDINE 4mg (2.3%) and 3TC (4.7%) groups, dizziness, followed by increased ALT, GGT, AST and blood uric acid. The adverse reaction of dizziness was slightly higher in grades 1 and 2. Grade 3 adverse reaction occurred only in the AZVUDINE 3mg group and the 3TC group. The incidence was 2.4% and 4.7%, respectively, and no grade 4 adverse reaction of dizziness occurred. However, the adverse reactions of dizziness that occurred during the study may be related to the EFV (Efavirenz) drug.

In summary, AZVUDINE combination treatment with TDF and EFV significantly inhibited HIV-1 RNA replication, and the treatment effect is currently comparable to the first-line treatment plan 3TC + TDF + EFV. The treatment showed good safety, so that the three AZVUDINE dose groups and the 3TC group were similar in the total incidence and severity of adverse reactions.

2.5. CLINICAL STUDIES WITH COVID-19

FNC-Hope1 was a randomized, open-label, controlled clinical trial to evaluate the safety and efficacy of AZVUDINE tablets in the treatment of COVID-19, conducted at *People's Hospital of Guangshan County*, China.

Twenty individuals with COVID-19 with mild to moderate symptoms were randomly and equally assigned to 2 groups, experimental and control. Subjects who received conventional treatment with FNC + (no antiviral drugs) after diagnosis **reduced the time to become nucleic acid negative for the first time by -7.3 days**, compared to those who received conventional treatment alone (9.8). Subjects who received conventional treatment + FNC (no antiviral drugs) **reduced the time to nucleic acid**

negative for the first time by -6.8 days compared to those who received conventional treatment alone (11,30).

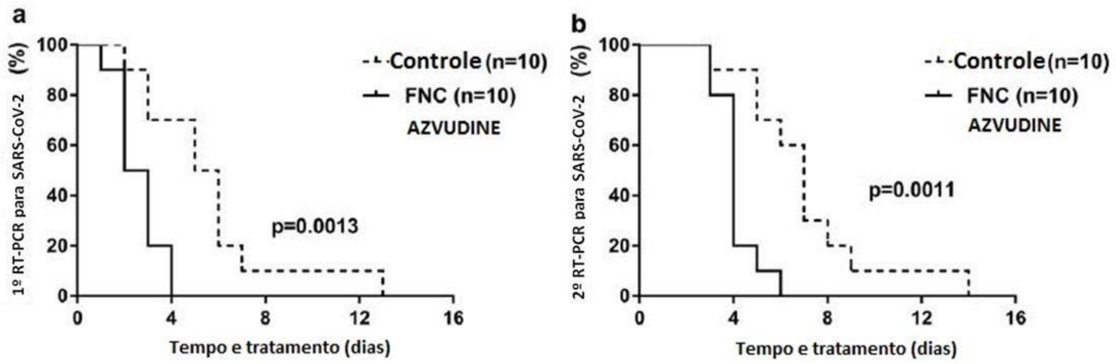

Figure 3: Kaplan-Meier curves of two consecutive negative tests for SARS-CoV-2 (a) first and b) second) in FNC and control group. Data are percent (%). Differences between groups were using the Log-rank test (Mantel-Cox)

On the fourth day after treatment, the rate of the first negative nucleic acid conversion reached 100% in patients in the FNC group and 30% in patients in the control group. Kaplan-Meier curves indicate the significant difference (p = 0.0013). On day 6, the negative conversion rate of the second nucleic acid reached 100% in patients of the FNC group and 40% in patients of the control group. Kaplan-Meier curves indicate the significant difference (p = 0.0011).

Regarding the safety analysis, **there was no adverse event in the experimental group** and three adverse events in the control group with an incidence of 30% (3/10) during the study period. All adverse events were grade 2 and were followed up until they disappeared. **There were no serious adverse events, serious adverse reactions, major adverse events, or death events during the study period.** The demographics and baseline characteristics of the subjects were similar and the proportions between the 2 groups were relatively balanced and comparable.

In 3 other hospitals in China, during the periods February 14 to March 10, March 1 to March 22, and March 7 to March 30, **32 volunteers with mild to severe symptoms were treated with different antiviral therapeutic approaches,** including Arbidol,

Lopinavir/Ritonavir, Interferon, Hydroxychloroquine, Osetalmivir, Ribavirin, traditional Chinese medicine drugs, and others, **prior to the start of AZVUDINE 5mg administration**. Some volunteers received an attack dose of 10mg on the first day. After starting AZVUDINE treatment, concomitant with some different combinations of these antiviral drugs, the **coronavirus viral load detection test became negative within 2-10days for 31 volunteers**. One patient reported **nausea and vomiting** on the first day of AZVUDINE treatment and another patient had **hypokalemia** two days after AZVUDINE administration. Both completed the treatment. One patient dropped out of treatment, but no further AEs were reported from these and other patients. **There were no serious adverse events (SAEs) or death events during the study period**.

As of January 20, 2022, 262 patients (expected enrollment is 342 patients, for the phase III study - Hope 4 and except for the last 3 patients who entered the group last, all other patients have been cured and discharged. Considering a 27% dropout rate, 250 patients are sufficient to prove superior efficacy (125 patients per group), so statistical analysis will be completed once participants are discharged and completing the study at the end of follow-up.

The adverse reactions in this study were mainly ALT and AST increase, mainly grade 1 adverse reaction, only 1 case of ALT elevation was grade 3 adverse reaction, and the outcome was normalization, and the adverse reactions were common adverse reactions of antiviral drugs. No unexpected adverse reactions occurred.

2.6. RISKS AND BENEFITS

2.6.1. Risks

The participant's health condition may remain the same, improve, or worsen, and there is the possibility of adverse effects occurring during the study. The procedures that will be performed throughout the research period may present some minimal risks related to the clinic visits, including, but not limited to, possible minor bruising due to

blood sampling for laboratory analysis, anxiety due to questioning about health status, and side effects due to the use of the AZVUDINE drug.

Like all medicines, **AZVUDINE can also cause some adverse reactions**. The main reactions reported in preliminary studies with the **4mg** dose **(fever, headache, dizziness, nausea, vomiting, and diarrhea**) were mild to moderate in intensity. Studies with a loading **dose of 10 mg and maintenance with 5 mg showed a good safety profile**. As the doses may overload the renal and hepatic systems, special attention will be paid to the exclusion criteria, and for the participants included in the study, there will be monitoring of liver and kidney function for intervention if necessary. Should even minimal adverse reactions occur, the research team will be available to provide all necessary assistance.

2.6.2. **Benefits**

There may be no benefit in participating in the study, but in any case, we believe that the search for an effective and safe therapeutic option is fundamental and a priority, and so it is necessary to conduct a randomized study along the lines of this proposal. However, **since this drug has already been shown to be effective in patients with COVID-19 (with the conversion of the viral nucleic acid to negative in a shorter time), a potential direct benefit to participants receiving AZVUDINE during the study can be expected**. Patients in the PLACEBO group will have the assistance recommended for treatment of COVID-19, and all participants will have disease follow-up through monitoring, consultations, and testing.

2.6.3. **Analysis of risks and benefits**

Phase I and II clinical studies have provided a safety profile for the use of AZVUDINE in HIV-positive volunteers at doses up to 4 mg. ***In vivo* studies have shown safety at doses at least 1000 times higher than those used in Phase I and II studies**. **Adverse reactions observed with 4mg of AZVUDINE were mainly fever, headache, dizziness, nausea, vomiting, and diarrhea, and the intensity of these AEs was mild to moderate.**

Preclinical testing for carcinogenesis and reproductive toxicity has shown that the doses used in the clinic are significantly lower than the doses that showed toxic effects in these trials. **No EAGs have been reported in the 48-week clinical trials conducted with AZVUDINE**. **However, because it is a nucleoside analog, there is a risk for fetal malformation, underscoring the importance of the exclusion criteria and follow-up of couples of childbearing age included in the study.**

Preliminary studies conducted with volunteers with COVID-19 used doses of 5 mg of AZVUDINE and some volunteers received a loading dose of 10 mg of AZVUDINE. The data from these studies showed a good safety and efficacy profile relative to patients previously treated with other therapeutic approaches, where there was no conversion of viral nucleic acid to negative and no improvement in the clinical picture. In volunteers, after AZVUDINE administration, the two consecutive viral nucleic acid tests were negative and all patients were discharged. The AEs reported were mild disappearing without medical intervention and no EAGs occurred.

It is important to note that the loading doses may overload the renal and hepatic system due to increased metabolism and excretion of the drug. Given of this **risk, special attention should be paid to the eligibility criteria, and for research participants included in the study, it is important that monitoring of liver and kidney**

function be performed in the first few days, so that intervention can take place if necessary.

A potential direct benefit to participants who receive this drug during the study can be expected to be the conversion of the viral nucleic acid to negative in a shorter time. It is important to note some risks and benefits attached to the randomized trial model, such as the possibility of the research participant being allocated to the PLACEBO group, i.e., without intervention of the experimental drug, which may be a promising treatment, but is not yet available in clinical practice. However, **participants in the PLACEBO group will have the assistance recommended for treatment of COVID-19, and all participants will have constant monitoring of the progress of the disease through consultations and examinations.** In addition, participation in a clinical trial requires greater availability of the study participant, as there is a need for frequent follow-up.

There will be an intensive follow-up of the patient in the hospital unit. The evaluations will be made by trained professionals and any alterations will be identified and appropriate actions will be taken to minimize and/or recover them. In addition, the participant will have access to and guidance regarding all the tests performed. **At any time, the participant may have access to all information obtained about him or her from this study, or about the overall results of the study after its termination. Withdrawal and/or termination of the trial will not affect the medical care offered to the participant, as control visits are scheduled.**

All medical information will be confidential and only the study team will have access to avoid a breach of confidentiality. At no time will the participant's name or any information about their health be provided to anyone other than the study team. The information will be confidential and used only for the purpose of this research. All reasonable measures to avoid breaching the participant's identity and confidentiality will be taken. These include access to study documents only for those on the research team,

storage of printed documents in a locked file in a location with restricted access, and storage of electronic data in a database with secure access via individual username and password only for the researchers and other members of the research team.

By proving the clinical efficacy and safety of AZVUDINE, society will be able to benefit from an effective treatment against the new coronavirus.

2.7. JUSTIFICATION

The coronavirus emerged in late 2019, and since then it has been spreading around the world with high speed. In March, the World Health Organization declared a pandemic of the new coronavirus, a result of the high spread of this virus worldwide. Brazil is currently the second country with the highest number of cases, exceeding 200,000 deaths from COVID-19 (WHO, 2020e). As a consequence, the coronavirus has not only led to a large number of deaths to date but has also overburdened the health care system and impacted the economy worldwide and in Brazil.

A key strategy in the treatment of COVID-19 would be to find an effective, antiviral agent that leads to a reduction in viral load and, consequently, a reduction in the physiological damage caused by the coronavirus. Clinically approved drugs or drugs in advanced stages of development for the treatment of other viral diseases, such as HIV, should be considered for the treatment of SARS-CoV-2.

AZVUDINE is an antiviral drug that was developed for the treatment of HIV, and its safety and clinical efficacy have been demonstrated in HIV-positive patients. Clinical development of this drug is currently in phase III in China. Also, in vitro studies have shown that AZVUDINE has effective antiviral activity against SARS-CoV-2, and preliminary clinical trials conducted in China have also shown potential efficacy and safety of AZVUDINE in patients with COVID-19.

The number of cases and deaths from COVID-19 has been increasing worldwide and in Brazil in recent months. It is necessary to quickly find alternative therapies to control the complications of SARS-CoV-2 infection, since the development, immune response, and recovery from the disease caused by this virus are still uncertain.

This study aims to generate rapid, robust, documented evidence on the potential clinical efficacy and safety of AZVUDINE, for patients infected with SARS-CoV-2.

3. HYPOTHESIS

AZVUDINE has a therapeutic potential and safety profile for the treatment of patients with pneumonia caused by SARS-CoV-2.

4. OBJECTIVES AND OUTCOMES OF THE STUDY

**Primary Objectives**	**Primary Outcome**
To evaluate the **efficacy and safety of** AZVUDINE (FNC) versus placebo in patients infected with moderate to severe stage SARS-COV-2;	• Proportion of participants with clinical status improvement [Period: until D15, approximately 14 days].<br>o The criterion for a participant to have an improvement in clinical status is a decrease on the WHO Clinical Improvement Ordinal Scale in at least one category compared to screening (WHO, Jun/2020).

Secondary Objective	Secondary Outcome
To evaluate the **clinical outcome** between the AZVUDINE (FNC) group versus placebo in patients infected with moderate to severe stage SARS-COV-2;	• Proportion of participants with a clinical outcome of cure during the study; [Period: until D15, D21, and D28, approximately 14-30 days].<br>○ The clinical endpoint of cure is defined in this protocol as the absence of viral RNA in collected samples and clinical conditions for hospital discharge[4] .<br>• Time to normalization of body temperature (below 37.6°C axillary); [Period: until D15, D21 and D28, approximately 14-30 days]<br>• Time to improvement of diarrhea, myalgia, fatigue, malaise, cough, dyspnea, and headache; [Period: until D15, D21, and D28, approximately 14-30 days].<br>• Changes in liver and kidney function *baselines*, and inflammatory and immunological markers: [Period: until D15, D21 and D28, approximately 14-30 days]<br>○ Lab tests performed on days D1, D3, D5, D7, D9, D13, D15, D21, D28, and D60.<br>• Evaluation of the time to negative conversion of the SARS- CoV-2 viral load by RT-PCR between AZVUDINE (FNC) group and control group; [Period: until D15, D21 and D28,approximately 14-30 days].<br>• Evaluation of the number of cycles for the detection of SARS-CoV-2 viral load by RT-PCR and application of the

[4] According to the WHO Working Group on Characterization and Clinical Management of COVID-19 Infection (July, 2020), clinical and virological absence of infection (non-detectable RT-PCR) is suggestive of CURE for initially infected participants.

	standard curve for viral load calculation; [Period: until D15, D21 and D28, approximately 14-30 days].<br>• Analysis of the relationship between the calculated viral load and the clinical progress of participants in the AZVUDINE (FNC) group compared to the control group; [Period: until D15, D21 and D28, approximately 14-30 days].<br>• Time and proportion of lung imaging improvement during treatment (Pattern of ground-glass opacities, mosaic paving, alveolar consolidation, reticular pattern/septal thickening, opacity with inverted halo, pleural/pericardial effusion, fibrosis and/or lymphadenomegaly); [Period: until D15, D21 and D28, approximately 14-30 days].<br>• Time of improvement and proportion of respiratory symptoms and signs during treatment (Lung rales, cough, sputum, sore throat); [Period: until D15, D21, and D28, approximately 14-30 days].<br>• Time to normalization of O_2 saturation (above 95%); [Period: until D15, D21 and D28, approximately 14-30 days].<br>• Time to normalization of respiratory rate to levels ≤ 24rpm, in room air; [Period: until D15, D21 and D28, approximately 14-30 days]<br>• Frequency of supplemental oxygenation or non-invasive ventilation; [Period: until D15, D21 and D28, approximately 14-30 days].<br>• Frequency of invasive mechanical ventilation or extracorporeal membrane oxygenation (ECMO); [Period: until D15, D21, and D28, approximately 14-30 days].

	• Proportion of moderate cases that progressed to severe cases requiring intensive care unit (ICU) care; [Period: until D15, D21, and D28, approximately 14-30 days].<br>• Length of hospital stay; [Period: until D15, D21 and D28, approximately 14-30 days].<br>• Occurrence of drug interactions; [Period: until D15, D21 and D28, approximately 14-30 days].<br>• Frequency and intensity of adverse events; [Period: until D15, D21 and D28, approximately 14-30 days].<br>• Frequency and intensity of unexpected adverse events; [Period: until D15, D21 and D28, approximately 14-30 days].<br>• Frequency and intensity of serious adverse events; [Period: until D15, D21 and D28, approximately 14-30 days].<br>• All-cause mortality rate during the study; [Period: until D15, D21 and D28, approximately 14-30 days].<br>• Evaluate the tolerability of AZVUDINE (FNC) use at 5mg/day regimen for up to 14 days: [Period: until D15, approximately 14 days].<br>○ Calculating treatment adherence;<br>○ Total time of use of AZVUDINE (FNC);<br>○ Proportion of participants who withdrew consent;<br>○ Proportion of participants who dropped out of treatment.

4.1. JUSTIFICATION OF PRIMARY OUTCOMES

According to the document "COVID-19: Developing Drugs and Biological Products for Treatment or Prevention" (FDA, 2020)[5] the evaluation of an investigational product for the treatment of participants with COVID-19 should adopt as an efficacy endpoint, measures that demonstrate clinical significance compared to the control group. As a suggestion, WHO advises the use of ordinal clinical outcome scales to assess the clinical progression of participants over a specific period. The categories described in the scale should be clearly defined and the corresponding levels should be discrete and non-overlapping (mutually exclusive) to avoid ambiguities in data analysis[6].

The WHO Clinical Improvement Ordinal Scale (Jun 2020) is a tool commonly used in clinical trials as a measure of clinical outcomes. On this scale, the WHO Working Group on Characterization and Clinical Management of COVID-19 Infection (July, 2020) presented an 11-point ordinal scale built on literature review and input from international experts in clinical trials, epidemiology, virology, infectious diseases, intensive care medicine, public health, and public policy. This scale was intentionally proposed as a minimum set of ratings, which focus on the most relevant variables for most participants included in cohort studies or clinical trials. The group also states that the expanded categories of this ordinal scale (compared to previously published scales, which were 8-point) allow for better distinction of participants' clinical status, especially at the lower levels of severity[7] .

[5] FDA. *COVID-19: Developing Drugs and Biological Products for Treatment or Prevention - Guidance for Industry*. USA. Maio/2020. Disponível em: https://www.fda.gov/emergency-preparedness-and-response/mcm-issues/covid-19-related-guidance-documents-industry-fda-staff-and-other-stakeholders

[6] MacKenzie CR, Charlson ME. *Standards for the use of ordinal scales in clinical trials*. Br Med J (Clin Res Ed). 1986;292(6512):40-43. doi:10.1136/bmj.292.6512.40

CLINICAL PICTURE	DESCRIPTION	SCORE
Uninfected	Non-infected; no viral RNA detection.	0
Outpatient: mild illness	Asymptomatic; viral RNA detected.	1
	Symptomatic; does not require assistance.	2
	Symptomatic; needs assistance.	3
Hospitalized: moderate illness	Hospitalized; no oxygen therapy*	4
	Hospitalized; oxygen support by mask or nasal catheter.	5
Hospitalized: serious illness	Hospitalized; oxygen support by NIV or high flow.	6
	Intubation and mechanical ventilation, pO2/FiO2 150 or SpO2/FiO2 ≥≥200.	7
	Mechanical ventilation pO2/FiO2 <150 (SpO2/FiO2 <200) or vasopressors.	8
	Mechanical ventilation pO2/FiO2 <150 and vasopressors, dialysis or ECMO.	9
Death	Death.	10

[7] John C Marshall *et al*. A minimal common outcome measure set for COVID-19 clinical research. The Lancet Infectious Diseases. Volume 20, Issue 8. (2020): e192-e197. ISSN 1473-3099. https://doi.org/10.1016/S1473-3099(20)30483-7.

CLINICAL PICTURE	DESCRIPTION	SCORE
ECMO: extracorporeal membrane oxygenation; FiO2: fraction of inspired oxygen; NIV: non-invasive ventilation; pO2: partial pressure of oxygen; SpO2: oxygen saturation; *If hospitalized for isolation only, register as an outpatient.		

Table 4: WHO Clinical Progression Scale (Jun 2020)

According to the working group, clinical and virological absence of infection is suggestive of CURE for participants who were initially infected (RT-PCR not detectable) or suggestive of misdiagnosis in inclusion of participants (WHO, 2020).

The maximum treatment time of 14 days respects the average incubation time of SARS- CoV-2 during infection, although previous trials have shown that negative viral RNA detection seroconversion in participants using AZVUDINE (FNC) can occur within 5 days. In the HOPE-1 trial, it was observed that two participants in the control group did not have negative viral RNA detection seroconversion in this period.

5. MATERIAL AND METHOD

5.1. STUDY DESIGN

5.1.1. Design

This is a randomized, parallel, double-blind, PLACEBO-controlled clinical trial, which after initial study data, the sample size calculation was revised by updating parameters of frequency of clinical improvement and dropout rate. The revised sample size is 180 participants randomly and equally divided into the two arms of the study (90 practitioners in each group).

In the experimental group, the treatment of participants using AZVUDINE associated with the standard treatment for health facility COVID-19 will be observed, and in the control group, the standard treatment for health facility COVID-19 associated with PLACEBO will be observed.

Below is Table 5, demonstrating the treatments and dosages:

Group	Number of Participants	Treatment	Dosage
Experimental Group	90	AZVUDINE +	5 mg/day, orally, up to 14 days (1mg/tablet)
		Standard treatment	-
Control Group	90	PLACEBO + 1	5 tablets/day, orally, up to 14 days
		Standard treatment	-

Table 5: Study groups

The study consists of 3 periods: **screening, treatment, and follow-up.**

During the screening period, patients with moderate to severe symptoms suspected of having COVID-19 who wish to participate in the research must authorize, by signing the informed consent form, the collection of nasopharyngeal/oropharyngeal *swab* samples for RT-PCR analysis. After analyzing the results of laboratory tests, the investigating physicians evaluate and validate the data that determine the eligibility of the volunteers, and they will be able to start treatment, after randomization.

During the treatment period, participants randomized to the experimental group will receive the experimental medication **AZVUDINE + standard treatment** and those randomized to the control group will receive **PLACEBO + standard treatment**. Research participants will receive **daily oral administration of the investigational products for up to 14 days, or until two consecutive negative RT-PCR viral load results are obtained**.

The principal investigator and his/her team should provide the participants with corresponding treatment measures according to the subjects' clinical symptoms, ensuring their safety during the trial. After completion of treatment and evaluation of clinical symptoms by the investigating physicians, the participant can be discharged from the hospital.

For **Early Termination (ETA)** of treatment, participants should follow the routine for visit 17: End of Treatment (D15), in advance. Discharge from the hospital may be

granted before the end of the 14-day treatment period if clinically indicated and the treatment has been completed.

The period between randomization and the end of treatment will be up to 14 days and there may be early termination (AT) or worsening. Participants who, even after the end of treatment, still remain in the hospital, will undergo the procedures and tests for safety and efficacy monitoring on D21 and follow the follow-up schedule from D28 to D60.

During the follow-up period, the participant will be required to return within 28 and 60 days of hospital discharge for follow-up visits.

The first day of treatment will be day 1 and will last up to 14 days.

Treatment will be terminated early when two consecutive RT-PCR tests are negative (criteria for hospital discharge) and may be stopped early in the event of participant dropout, or in the event of EAG.

All participants will undergo efficacy and safety evaluations. Blood samples and respiratory samples (oropharyngeal and nasopharyngeal swabs) will be obtained on days D1, D3, D5, D7, D9, D11, D13, and D14 (while hospitalized); and day D21 (participants still hospitalized after completion of treatment) and days D28 and D60 (upon return to the center or if still hospitalized). Detailed information about the visit is in section 7.3.

After discharge from the hospital, research participants should follow the instructions below in accordance with the care and attention specified by the research center:

- Stay in recovery for 3 to 6 weeks;
- Use face masks when necessary;
- Sanitize your hands and do not share personal objects;
- Avoid contact with people in the risk group, especially;

- Inform the Principal Investigator, or his/her team, immediately in case of fever and/or respiratory symptoms.

5.1.2. Justification for the choice of design

The maintenance of the phase III design was an evaluation by ANVISA, after approval by the CEP and CONEP, according to the meeting on 02/22/2021: *"in the Agency's assessment, the report presented with data from the Hope 4 study is sufficient to support the phase III study without the need for interim analysis. Also, the monitoring committee will evaluate adverse events during the study.* The Phase III study of AZVUDINE for COVID19 in the 5mg/day dose regimen for up to 14 days is still being completed in China. Of 342 participants, 262 have already been discharged from the hospital and no EAGs have been reported. The sponsor has provided the partial results confidentially to ANVISA since the data have not yet been published.

The therapeutic regimen proposed in this study is based on preliminary studies conducted in China in patients with pneumonia caused by SARS-CoV-2. **The dose used was 5mg, and the AEs observed were mild to moderate, with no need for medical intervention.** Some patients received an attack dose of 10mg on the first day, and no AEs more severe or different from those seen with the 5mg regimen were observed.

For this study, **the proposed treatment period is 14 days**, given the average coronavirus incubation time and the AZVUDINE usage time that **in the preliminary studies, ranged from 2 to 12 days**.

There is not yet an official treatment for the treatment of COVID-19, but the evidence from Hope I, discussed earlier, shows that individuals receiving conventional treatment + FNC (without antiviral drugs) after diagnosis **reduced the time to nucleic acid negative for the first time by -7.3 days** compared to those receiving conventional treatment alone (9,8). Subjects who received conventional treatment + FNC (no antiviral drugs) reduced the time to nucleic acid negative for the first time by **-6.8 days** compared to those who received conventional treatment alone (11,30).

In the phase III study - Hope 4, with 342 participants, 262 participants, except

for the last 3 patients who entered the group last, all other patients were cured and discharged. In terms of safety events, with the exception of individual patients who had skin rash or elevated ALT, other adverse events were probably unrelated and no serious adverse events were reported. The adverse reactions in this study were mainly ALT and AST increase, mainly grade 1 adverse reaction, only 1 case of ALT elevation was grade 3 adverse reaction, and the outcome was normalization, and the adverse reactions were common adverse reactions of antiviral drugs. No unexpected adverse reactions occurred. There were no serious adverse events, serious adverse reactions, major adverse events, or death events in the study period.

Recently (2020), the journal NATURE published the article *AZVUDINE (FNC): a promising clinical candidate for COVID-19 treatment*[8], which considers AZVUDINE a promising candidate for the treatment of COVID19, demonstrating its potential in inhibiting viral replication.

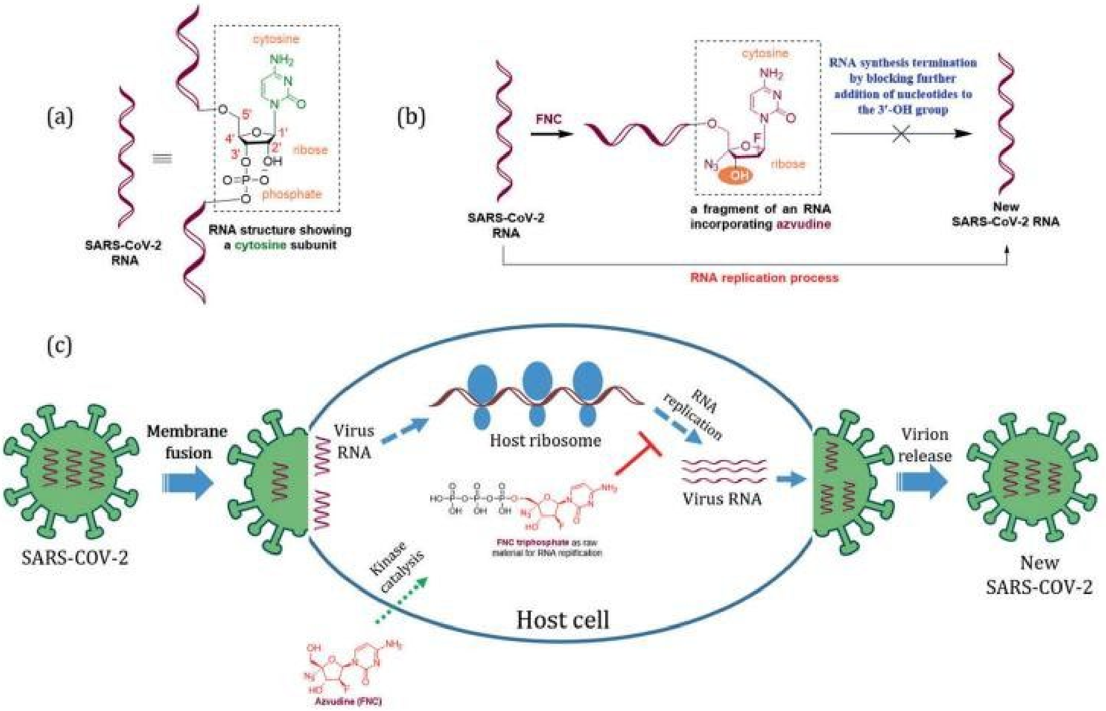

Figure 4: Mechanism of action of AZVUDINE a) Azvudine nucleoside-based anti-HIV clinical candidate (FNC) for the treatment of COVID-19. Structure of SARS-COV-2 RNA showing a cytosine subunit; b) Diagram showing the RNA replication process of SARS-

COV-2 blocked by FNC; c) Proposed mechanisms of FNC to inhibit SARS-COV-2 activity[1].

To minimize study bias, research participants will be randomized and treatment will be administered blindly, both by research center staff and to participants. The research center's standard treatment will be used to avoid any complications/aggravations of the disease and PLACEBO will be used for masking purposes only.

5.1.3. Justification for dose choice

The dose of AZVUDINE that will be used in this study is the same as that used previously in exploratory studies in China to treat patients as COVID-19. The investigational drug **in** question **is a new type of nucleoside analog reverse transcriptase inhibitor that is showing good clinical efficacy in treating patients with SARS-CoV-2 infection in China.**

Currently the phase III study (FNC-Hope4), with 342 participants, has 262 participants, where the dose used was 5mg/day for up to 14 days, with no EAG reported. A partial, confidential report has been issued to ANVISA (ANNEX 5).

The therapeutic regimen proposed in this study is based on preliminary studies conducted in China in 41 patients with pneumonia caused by SARS-CoV-2. **The dose used was 5mg/day, and the AEs observed were mild to moderate, with no need for medical intervention.** All patients were discharged completely from the hospital after two consecutive RT-PCR tests. Of these 41 patients, 8 volunteers used an attack dose of 10mg on day 1 and 5mg on subsequent days. **The final report (FNC-Hope1) showed benefit to participants treated with AZVUDINE, while on other therapeutic approaches they had no improvement in clinical picture and nucleic acid detection did not turn negative.**

Regarding the dose-related safety profile used in humans, the phase I dose explorer clinical study (GQ-FNC-2014), evaluated the safety of AZVUDINE in HIV-positive

[8] Yu, B., Chang, J. AZVUDINE (FNC): a promising clinical candidate for COVID-19 treatment. Sig TransductTarget Ther 5, 236 (2020). https://doi.org/10.1038/s41392-020-00351-z (https://www.nature.com/articles/s41392-020-00351-z)

patients for 7 days and demonstrated that AZVUDINE has clinical safety. The AZVUDINE dose regimen groups for 2mg, 3mg, 4mg, and 5mg showed good safety. No AEs were reported in this study and no participants in the AZVUDINE 3mg and 5mg groups reported any AEs. No serious adverse events were attributed to AZVUDINE.

In this trial**, the plasma concentration of AZVUDINE in patients receiving a 5 mg dose was approximately 2.42ng/mL,** which is much lower than the plasma exposure level of the drug at the NOAEL dose (5.6 mg) in animals (C_{max} in mice: 164-210.27 ng/mL,

C_{max} in dogs: 85.84-102.55 ng/mL) and 3.099 times lower concentration the dose used in the CHL cell chromosome structure aberration test with the concentration of 7.5 µg/mL after the 4-hour exposure with or without S9 metabolic activation system.

In this same trial, volunteers receiving 3mg and 4mg of AZVUDINE in a single dose had a plasma exposure of approximately 2.85 and 3.4 ng/mL, respectively. The AUC_{0-t} gradually increased in the 3mg, 4mg and 5mg groups when compared to the 2mg group, with a statistically significant difference between the 4mg and 3mg groups.

Pharmacokinetic data suggest that FNC is rapidly absorbed and slowly eliminated. Total urinary excretion of AZVUDINE increases with increasing dose. Excretion between 0-12h accounted for more than 70% of total excretion in 0-24h.

For the 5 mg dose of AZVUDINE, the average half-life is 13.8h, and it is excreted in the urine in up to 24h.

In the exploratory clinical trials in China, a higher than safety-verified dose (5mg) was used with a dosing regimen of "On Day 1 10mg" in 8 cases at Henan Provincial People's Hospital and yet there were no serious adverse events, serious adverse reactions, major adverse events or death events in the study period. The current dosing design does not use the first dose of 10mg and the current ongoing Phase III clinical trial in China, the dosage is "5mg daily".

In vivo toxicity results from multiple doses of FNC, at high dose in rats (human

equivalent 11.2-56 mg) and dogs (human equivalent 11.16-37.20 mg).

Preclinical trials evaluated much longer administration times than suggested in this clinical trial, leading to a total of the equivalent human dose used in these trials from 116.6 mg (1 month) to 1,088.10 mg (39 weeks).

Animal / human equivalent dose	Animal / Administration time	Total dose used in pre-clinical tests over time
3.72mg	**1 month**	116.6mg
5.60mg	**3 months**	504.0mg
3.36mg	**6.5 months (26 weeks)**	655.2mg
3.72mg	**9.75 months (39 weeks)**	1,088.1mg

Table 6: Relationship between dose and administration time

The **maximum dose of AZVUDINE proposed for this clinical trial is UP TO 70 mg in UP TO 14 DAYS, remembering that the participant can be discharged earlier, reducing the total dose.** Long-term exposure to the drug has shown that **observable adverse events appear when the dose is exceeded 20-30 times** in animals.

Considering that the maximum safety dose study for AZVUDINE was performed for 5mg, a daily dose of 5mg can meet clinical treatment. The proposed dose of 5mg in the clinical trials for COVID-19 is slightly lower than the lowest NOAEL dose in the fertility and early embryo development toxicity trials (5.6 mg).

Based on these studies, there is increasing evidence that the 5mg dose may have clinical efficacy against SARS-CoV-2 infection and its safety profile is compatible with use in the proposed target population.

5.1.4. **Research location**

The present research project will be conducted at Santa Casa de Misericórdia de Campos, located in Parque Tamandaré, Campos dos Goytacazes - RJ. The research center

was pre-selected by the study sponsor due to the profile of the Northern region of the State of Rio de Janeiro, composed of 8 municipalities, with the city of Campos dos Goytacazes - RJ being the most populous and the destination of patients from the macro region.

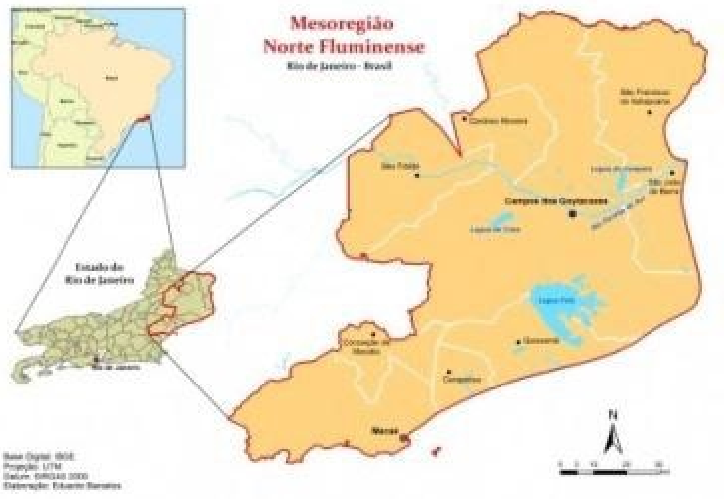

Figure 5: Macroregion of Norte Fluminense

The research center was qualified by the Study Sponsor in compliance with the standards established by the ICH Guidelines for Good Clinical Practice GCP/ICH - ICH *Topic* E6 (R2) (2016), ANVISA Normative Instruction nº. 20, 2017, which provides for inspection procedures in Good Clinical Practice for Clinical Trials with Investigational Medicines and other applicable regulations.

The choice occurred due to the infrastructure necessary for the flow of the participants' hospitalization and optimization of the logistics of molecular biology analyses. Centralizing the analyses in the SCMC Molecular Biology Laboratory (equipped by the Galzu Institute and certified by ViSa-RJ) and also installing the study's biorepository at the State University of Rio de Janeiro (UENF) were strategic decisions to maintain the quality of the process in the proof of concept (we will explain later).

Renowned national research institutes, such as FIOCRUZ, use the strategy of multicenter models in their studies. However, in studies with the same design profile,

the participating centers are all in metropolitan areas of Rio de Janeiro, most concentrated in the municipality itself. Such a strategy certainly favors the catchment strategy, but it does not bring great contributions to evaluations of the influence of demographic or epidemiologic profiles on the data of the studies. Also, most of these studies involve outpatient follow-up; different from our proposed study, which involves inpatient care and, for this reason, demands a more complex infrastructure to set up a research center.

5.2. STUDY POPULATION

5.2.1. Recruitment and Selection

The research center is responsible for defining the strategies for recruitment and initial screening of research participants, so the **recruitment strategy will be the healthcare network**, since patients with moderate to severe stage COVID-19 symptoms eventually seek hospital care.

The fundraising strategy foresees the attraction of volunteers, through agents of prospection, in the entire region of Norte Fluminense, in addition to patients who seek care locally. The recruitment also involves partnerships with the Health Secretariats of the North and Northwest Fluminense region and their municipalities, which do not have a robust infrastructure for hospital care.

Municipalities: Campos dos Goytacazes, Cardoso Moreira, São Fidélis, São Francisco de Itabapoana, São João da Barra, Carapebus, Conceição de Macabu, Macaé and Quissamã, from the northern fluminense; and Itaperuna, Bom Jesus do Itabapoana, Italva, Laje do Muriaé, Natividade, Porciúncula, Varre-Sai, Santo Antônio de Pádua, Aperibé, Cambuci, Itaocara, Miracema, and São José de Ubá, from the Fluminense northwest.

After screening with the nursing staff of the health units, axillary temperature,

blood pressure, pulse oximetry, heart rate, respiratory rate, and the presence of risk factors (such as age over 65 years, hypertension, diabetes, cardiovascular disease, cerebrovascular disease, lung disease, immunosuppression, cancer, or use of immunosuppressive medications) **are checked.** An assessment by the qSOFA *score* should also be performed to find out if the patient has had contact with someone with suspected or confirmed COVID-19 and if the patient works in a healthcare facility or provides patient care. **The nursing team then classifies the management according to color: green (mild cases), yellow (moderate cases), or red (severe cases).**

Yellow rating means that it is a moderate stage II disease (inflammatory stage). In this stage, the patient may present with persistent cough plus daily persistent fever or persistent cough plus progressive worsening of another symptom related to COVID-19 (adynamia, prostration, hyporexia, diarrhea, decreased general condition, significant myalgia, moderate dyspnea, with or without hypoxia) or at least one of the above symptoms plus the presence of a risk factor. At this stage, the management is **hospital admission in a ward/apartment.**

If hospitalization is indicated, a rapid test for COVID-19 **will be performed,** and if the result is positive, the clinical study will be presented by the prospecting team and an invitation to participate in the study will be extended. The consent process will be conducted by the prospecting agents in the field, duly delegated and trained to conduct the process, as well as to apply the Informed Consent Form (ICF). Multimedia resources will be used to clarify and instruct the volunteer about the objectives and details of the study. At the end of all the necessary clarifications, the volunteer will be asked to sign the ICF, where one copy will remain with the volunteer and another will be destined to the research center.

If the patient accepts to be a study volunteer, he/she and his/her companion, if applicable, will be informed of the study procedures, including the laboratory tests for the screening period, the criteria for inclusion and exclusion from the study, his/her rights and duties, the period of treatment and follow-up. **The eligibility criteria may be**

explored after the anamnesis, laboratory tests, and imaging tests have been performed, but **may** also **be suspended at any time prior to complete evaluation if exclusions are identified by the study team.** After this phase, the participant will be **invited to sign the ICF** and, only after signing, the volunteer will be included in the study. Clinical and laboratory evaluations and collection of nasopharyngeal and oropharyngeal *swab* specimens for RT-PCR testing **confirm the eligibility of the study participant and will be assessed once all screening values are available.**

Research participants who meet the inclusion criteria and do not meet the exclusion criteria, and who have eligibility assessed by the Principal Investigator, **will be eligible**.

The **volunteer will then be transferred to Santa Casa de Misericórdia de Campos, via the regulatory center for hospital admission in a ward/apartment**, and will be **immediately randomized to** follow the Treatment Protocol for COVID-19 of Santa Casa de Misericórdia de Campos (**ANNEX1**), as listed below:

Initial assessment by the nurse, checking axillary temperature, blood pressure, pulse oximetry, heart rate, respiratory rate, and the presence of risk factors (such as age over 65 years, hypertension, diabetes, cardiovascular disease, cerebrovascular disease, lung disease, immunosuppression, cancer, or use of immunosuppressive medications). You should also perform a qSOFA score assessment, find out if the patient has had contact with someone with suspected or confirmed COVID-19, and if the patient works in a healthcare facility or provides patient care. After these steps, the nurse will classify the management according to the color: green (mild cases), yellow (moderate cases) or red (severe cases).

In the green classification (stage I - viral replication) the conduct is home treatment. The patient should be instructed to wear a mask, use home isolation, and return home if there is worsening.

The yellow rating signifies moderate stage II disease (inflammatory stage). At this stage, the patient may present with qSOFA=1, fever, decreased general condition, significant myalgia, persistent dry cough, diarrhea, moderate dyspnea. This stage is

divided into IIA, i.e., without hypoxia (SpO2 93-94% / CT <50%) or IIB, with hypoxia (SpO2 $_{92\text{-}93\%}$ / CT >50%). In this stage, the management is hospital admission in a ward. In Stage IIA, patients with SpO2 93-94%, but with pulmonary impairment <25%, in pandemic conditions with over-occupancy of beds and/or conditions to return to the hospital in case of clinical worsening, there is the option of not admitting the patient and prescribe according to the outpatient treatment protocol. Patients with underlying lung disease, pulmonary impairment between 25-50%, even with normal SpO2, should be hospitalized. Patients with chronic kidney disease on dialysis, pulmonary impairment and altered SpO2 should be hospitalized.

The red rating signifies severe stage III disease (inflammatory storm). At this stage, the patient may present with qSOFA ≥2, SpO2 <92% and pO2 <63mmHg even with dry O2 supply at 6L/min, acute respiratory failure/VM, RR >30 and/or use of accessory muscles or paradoxical breathing, septic shock (hypotension, SBP <90mmHg/ MAP <65 or signs of hypoperfusion, with lactate >36mg/dL), lowered level of consciousness and respiratory acidosis (pH <7.3, pCO2 >50mmHg). In this case, the management is hospital admission in ICU.

5.2.2. **Screening**

The research participants will be screened following the inclusion and exclusion criteria.

5.2.3. **Inclusion Criteria**

- Age ≥18 years, regardless of gender;
- Patients hospitalized in moderate to severe stages in line with the Ministry of Health classification[9];
- Positive diagnostic test for SARS-CoV-2 by molecular amplification of the virus in RT-PCR diagnosed from respiratory specimen (nasopharyngeal, oropharyngeal, lower respiratory tract [e.g., sputum) collected <96 hours before randomization;

[9]The Brazilian Ministry of Health classifies the symptoms of COVID-19 into:
Mild: Flu-like symptoms (cough, sore throat, or runny nose), followed or not by: anosmia (olfactory dysfunction), ageusia (taste dysfunction), runny nose, diarrhea, abdominal pain, fever, chills, myalgia, fatigue, headache;
Moderate: Persistent cough + daily persistent fever or persistent cough + progressive worsening of another symptom related to COVID-19 (adynamia, prostration, hyporexia, diarrhea) or at least one of the above symptoms + presence of risk factor.
Severe: Severe acute respiratory syndrome: influenza syndrome presenting with dyspnea/respiratory distress or persistent chest pressure or O_2 saturation (SpO2) less than 95% on room air or Bluish coloration of lips or face.

- Time between symptom onset and inclusion ≤14 days;
- Hospitalization within 48 hours of study inclusion;
- Availability for follow-up during the study period;
- Voluntary adherence to participate in the study and signing of the Informed Consent Form.

5.2.4. Exclusion Criteria

Individuals with one or more of the following characteristics will not be eligible to participate in this study:

- Patients known or suspected to be sensitive to AZVUDINE or excipients (inactive ingredients: microcrystalline cellulose, lactose hydrate, polyvinylpyrrolidone K30, croscarmellose sodium, magnesium stearate);
- Patients diagnosed with pneumonia caused by other pathogens;
- Patients with liver disease (total bilirubin ≥2mg/dL, ALT/TGP and AST/TGO ≥5 times above normal limit); [10]
- Total neutrophil count <750 cells/L;
- Patients with renal insufficiency (glomerular filtration rate ≤60mL/min/1.73m2) or are receiving continuous renal replacement therapy, hemodialysis, or peritoneal dialysis;
- Patients with congestive heart failure (NYHA grade 3/4), untreated symptomatic arrhythmias, or myocardial infarction within 6 months;

[10] Normal values: BT up to 1.2mg/dL; TGO Adult 12 to 46U/L; TGP Adult 03 to 50U/L; GGT Man 10 to 50U/L, Woman 07 to 32U/L (Instituto Hermes Pardini).

- Patients with a history of known liver disease (cirrhosis with *ChildPugh* classification B and C);
- Individuals with malabsorption syndrome, or other conditions that affect gastrointestinal absorption, and circumstances in which patients need intravenous nutrition, or cannot take medications orally or nasogastrically;
- Women who are pregnant or lactating, or of childbearing potential during the study period and within 6 months after termination of administration;
- Patients already included in other clinical trials;
- Patient under treatment for HIV, Hepatitis C, active Hepatitis B;
- Patients being treated with other antivirals (e.g. lopinavir/ritronavir, remdesivir, umifenovir/arbidol, favipiravir, interferon-α);
- Patients being treated with monoclonal antibodies (e.g. tocilizumab and sarilumab/kevzara);
- Patients who are on a clinical treatment plan that includes concomitant administration of any other **experimental treatment** or ***off-label*** use of already marketed drugs (e.g. hydroxychloroquine sulfate);
- Patients requiring invasive mechanical ventilation or extracorporeal membrane oxygenation (ECMO) at the time of randomization;
- Any clinically significant medical condition or medical history that, in the investigator's opinion, may discourage participation in the study, such as corrected QT interval >480 on the electrocardiogram, among other conditions.

5.3. VIROLOGICAL DIAGNOSIS

5.3.1. Diagnostic criteria for SARS- CoV-2 infection

The criteria for diagnosis will be based on the World Health Organization Guide, where confirmed cases of SARS-CoV-2 infection will be those performed by molecular testing with viral sequencing by QUALITATIVE real-time PCR /RT-PCR (WHO, 2020a) of nasopharyngeal and oropharyngeal *swab* specimens, bronchoalveolar lavage, nasopharyngeal tracheal aspirate, blood, serum and tissue from lung biopsies.

5.3.2. Process standardization at proof of concept

According to the protocol of the IGZ-1 study, RT-PCR tests will be done every 48h, and since we will probably have patient intake on odd and even days, processing should be done every day, with results every 24h.

Therefore, the ready availability of RT-PCR test results impacts the assessment of eligibility, total treatment time, and early termination of the participant's treatment.

We know that RT-PCR can demonstrate the negative conversion of the presence of virus copies (QUALITATIVE analysis), while a ddPCR can determine the number of virus copies (QUANTITATIVE analysis). However, due to the cost and unavailability of this equipment with ANVISA registration, the analysis must pass through validation, being infrequent in Brazil.

We also know that it is possible to calculate the amount of viral RNA copies by means of a standard curve to quantitatively determine the viral load of an unknown sample from samples with known amounts.

> *Wang et al, J Clin Invest. 2020 demonstrated that the CT values observed in respiratory samples from critically ill patients were lower (18-22) than those observed in mild patients (32-38). Therefore, the lower the CT, the higher the amount of viral RNA in the sample and consequently the higher the viral load of the volunteer.*

From these variables, we standardized the process to quantify the viral load as described below, since **very few studies relate the number of cycles with viral load, or with the efficacy of the experimental drug. This is a little explored problem that may**

bring to light issues of virus behavior in relation to clinical response, already questioned by the FDA.

According to FDA guidance "COVID-19: Development of Biologic Drugs and Products for Treatment or Prevention - Guidance for Industry; 2020," "... virologic endpoints are not appropriate as primary endpoints in a phase III study **because there is no established predictive relationship between the magnitude and timing of viral reductions and the extent of clinical benefit of how a patient feels, functions, or survives."**

THE PROBLEM

Each RT-PCR equipment has its own sensitivity, and different RT-PCR reagent kits have different performances, according to the May 2020 technical note "ANVISA - ACCURACY OF ANVISA REGISTERED DIAGNOSTIC TESTS for COVID-19 for qualitative analyses".

The sensitivity of each RT-PCR equipment and kit used varies in the number of CTs to generate the qualitative result (Positive or negative)[11].

So, to extract RELIABLE **QUANTITATIVE** data, both the equipment and the reagent kit must be standardized to avoid discrepancies. However, this is not enough, so a standard curve should provide the correlation between CTs and *viral RNA copy* number.

Therefore, we will use the QuantStudio5 RT-PCR equipment, *Applied Biosystems,* NS: 272525086, ANVISA Reg.: 10358940069, to issue reports for statistical accounting and to assist in the standard curve analysis, via dedicated software[12]. We will also use the TaqPathTMCOVID-19 CE-IVD RT-PCR[13] kit (ANVISA Reg.: 10358940107), which can provide us with the viral load-related CT values (viral RNA copies) as a function of the standard curve constructed with the positive control (TaqPath COVID-19 *Control*), which is SARS-CoV-2 viral RNA at a known concentration of 1 x ^{104} copies/µL. By means of the standard curve, using serial dilutions of the positive control, we can arrive at the

concentration (in number of copies) of the viral RNA present in each sample from the volunteers and participants.

For example, it is common to determine the concentration of proteins in a sample using a colorimetric method, the Bradford assay. Similarly, by performing serial dilutions of the positive control we can establish a standard curve (equation y = ax + b) using the CTs obtained for each point on the concentration curve.

---

[11] KiCqStartTM One-Step Probe RT-qPCR ReadyMixTM. Catalog Number KCQS07, KCQS08, KCQS09. SIGMA-ALDRICH
2019-nCov CDC Probe and Primer Kit for SARS-CoV-2 CERTIFICATE OF ANALYSIS. Catalognumber: KIT-nCoV-PP1-1000. Bioresearch Technologies.
2019-nCoV TaqMan RT-PCR Kit. Catalognumber:TM67100, TM67120. NORGEN BIOTEK COPORATION.
[12] Real-time PCR handbook, 2012. Basis of Real-time PCR, Life Technologies. Inc. CO32085 0812.
[13] TaqPathTM COVID-19 CE-IVD RT-PCR Kit INSTRUCTIONS FOR USE. Catalog Number A48067 Publication Number MAN0019215.

The positive RT-PCR result occurs at CTs ≤37. In this case, when viral RNA is present, the specific probe used for detection of SARS-CoV-2 is broken by DNA polymerase, emitting fluorescence. The higher the viral RNA copy number, the higher the fluorescence and therefore the CT value appears earlier during the reaction.

The lower the copy number of the viral RNA, the lower the fluorescence and, consequently, the CT value appears later. CTs values >37 are considered negative. By establishing a curve of viral RNA concentration (present in the positive control), we will obtain a curve of CTs, from lower values (higher viral RNA copies) to higher values (lower viral RNA copies).

The trials approved for the treatment of COVID19 so far use RT-PCR negative conversion monitoring as the endpoint, while the IGZ-1 assay methodology, in addition to obtaining a positive or negative result for the presence of SARS-CoV-2, can also calculate the number of viral copies in each sample and in a practical way:

- It would be possible to determine the behavior of the viral load after the use of the experimental drug and the placebo;
- It would be possible to relate the different viral loads of the positive result to the clinical improvement over time, individually and overall, in the two groups;
- It would be possible to observe the scale of growth (exponential or logarithmic)

and the best time for intervention.

In a positive test, there is a significant range of variability in viral RNA copies that may be related to the clinical condition of the participant, and since viral load monitoring will be done every 48h, an algorithm can be reached to explain why positive people have different outcomes.

5.4. MEASURES TO MINIMIZE STUDY BIAS

5.4.1. **Randomization**

Research participants, according to eligibility at the time of screening and agreement of the Principal Investigator, will be randomized to one of the two study arms (1:1) by Research-IGZ 1.0 software, generating via the system, random numbers that relate the groups to the volunteers, from the data management plan in **ANNEX 2.**

5.4.2. **Blinding**

A physical report of the blinding will be generated as a security measure and sent to the pharmacist for dispensing the investigational product and to the center coordinator in a sealed envelope. Details about blinding can be found in the Randomization topic in **ANNEX 2.**

5.4.3. **Breaking the emergency blinding**

EMERGENCY CEGENE BREAKING can be **INDIVIDUAL or TOTAL**, via the system through the administrator password of the Research-IGZ 1.0 software.

Breaking the blinding must be justified by the Principal Investigator, and it is certain that any breaking of blinding will generate Logs in the system, which can be seen in the audit analyses. In case of unblinding, the Principal Investigator and the center coordinator will opt for INDIVIDUAL unblinding. Only in recurring SAEs, suspected of being related to the investigational product, will the FULL blinding be broken.

5.4.4. Rules for breaking the blinding

This study adopts the double-blind method:

Primary disclosure will be performed after completion of the final version of the statistical plan, audit report and database lock. At this stage, the random numbers will be grouped according to the study arm for statistical analysis by group.

Secondary disclosure discloses the drug corresponding to the group code, experimental drug or PLACEBO. Disclosure documents will be signed by the Sponsor and Research Coordinator.

6. TRATAMENT OF PARTICIPANTS

For the purposes of this protocol, AZVUDINE is the investigational drug that will be administered concomitantly with the standard treatment for COVID-19, as per healthcare organizations' guidelines. The investigational drug will be compared to PLACEBO, also administered concomitantly with the standard treatment for COVID-19. When referring to the investigational (AZVUDINE) or control (PLACEBO) product, the term experimental drug or PLACEBO will be used.

6.1. STANDART TREATMENT GIVEN IN THE CARE OF PATIENTS WHIT COVID-19

The Principal Investigator and his/her team should follow the research center's own COVID-19 treatment protocol as described below, providing participants with treatment measures consistent with the individuals' clinical symptoms and recommended by the Ministry of Health (MS/SCTIE, 2020).

For **research participants with moderate symptoms hospitalized**, all isolation care, signaling, information to the other participating teams (nutrition, hygiene, physiotherapy, etc.) should be performed. Intravenous hydration, analgesia and antipyretics (dipyrone/ paracetamol), gastric protection (with proton pump blockers), glycemic control, antimicrobial treatment (azithromycin and/or ceftriaxone, levofloxacin, moxifloxacin, clarithromycin, or ampicillin or tazobactam) must be performed. In addition, respiratory physiotherapy, oxygen therapy, and bronchospasm

management should be performed, following the existing flowcharts. To remove the patient from isolation, RT-PCR should preferably be performed, and if negative, the test should be repeated with an interval greater than 24 hours.

For **research participants with severe symptoms**, attention should also be paid in isolation care. Intubation should follow existing protocol. Early enteral nutrition therapy (ETT) should be entered via gastric route, preferably started within 24 to 36 hours of ICU admission or 12 hours after intubation in hemodynamically stable patients. Venous hydration should be with glucose and lactated Ringer's serum. The presence of circulatory shock is an important cause of death and the approach can be made taking into account the following phases: (1) rescue, (2) optimization, (3) stabilization, and (4) resuscitation, each phase with specific guidelines. Again, removal of the patient from isolation should preferably be based on RT-PCR testing, with the option of COVID-19 serology testing (anti-SARS-CoV-2 IgG and IgM antibodies) included.

The standard protocol of care indicates the use of some drugs such as chloroquine or hydroxychloroquine, oseltamivir, anticoagulation, corticosteroids, with specific instructions for each drug. However, specific recommendations should be made to the team involved in the study, so that no drugs prohibited in this study protocol are administered (see item 6.6).

6.2. EXPERIMENTAL TRATAMENT

6.2.1. Formulation and appearance

AZVUDINE: The lyophilized formulation of AZVUDINE is a preservative-free, white to yellowish, odorless and tasteless solid containing 1mg of AZVUDINE.

AZVUDINE is supplied as a sterile product in a white, round, wide-mouthed, high-density polyethylene vial with a polypropylene cap. In addition to the active ingredient, the lyophilized formulation of AZVUDINE contains the following inactive

ingredients: microcrystalline cellulose, hydrated lactose, polyvinylpyrrolidone K30, croscarmellose sodium, magnesium stearate.

PLACEBO: The manufacturing and packaging process for PLACEBO is the same as for the investigational drug, conducted by the same manufacturer and production line. The freeze-dried PLACEBO formulation provided is identical in physical appearance to the active freeze-dried formulation and contains the same inactive ingredients and will be used in the study for masking purposes.

6.2.2. Packaging and labeling

The investigational drug and PLACEBO will be shipped by the sponsor to the research center in white, round, wide-mouthed, high-density polyethylene vials with the country-specific label and polypropylene caps.

AZVUDINE tablets 1mg / PLACEBO

35 tablets /battle

FOR CLINICAL RESEARCH ONLY

Internal Use / Prescription according to Research Protocol

SEARCH CODE: IGZ-1

Producer: "Beijing Union Pharmaceutical Factory" China.

Address:No. 37, Yongwang Road, Bio-medicine Industry Park, Daxing District, Beijing, China.

ORPC/CRO: Galzu Institute for Research, Teaching, Science and Applied Technology.

Name of the Principal Investigator: Cleber Glória Silva.

Lot number: ________________ Date of manufacture: ___________

Patient randomization number ____________

(01)02003659821451(30)1200

Keep out of reach of children

Maintain at room temperature

Validity: 2 years

Figure 6: Clinical research drug label

Investigational products will be received by the center in separate boxes of AZVUDINE and PLACEBO, with serial number "X to Y" for AZVUDINE and serial number "Z to W" for PLACEBO, for example. There will be a label for participant identification during dispensing, which will be done by double checking.

Each bottle with 35 individual tablets will have a label with the information in Figure 1manufacturer's name and address, lot number, medication identification number, participant information, care statement, instructions for use and dosage (mg), expiration date, storage conditions, and an indication of "For clinical trial use ONLY".

All study medications will be prepared, packaged and labeled in accordance with the standard operating procedures (SOPs) of the sponsor or its representative, Good Manufacturing Practice (GMP) guidelines, International Council on Harmonization (ICH) Good Clinical Practice (GCP) guidelines and applicable national regulations. All labels on the packaging will comply with local regulations.

6.2.3. Storage and Stability

The shelf life of AZVUDINE and PLACEBO is 2 years. The storage of the experimental medicine and PLACEBO should be at room temperature, in a dark place.

The product proved stable in high temperature and humidity tests at 40°C and 60°C and at 92.5% RH in closed storage and, for up to 30 days, no significant changes were observed in the stability tests of the drug to be used in clinical trials in Brazil. Samples stored open at 75% RH on the 30th day showed moisture absorption of more than 5%.

6.2.4. Dose and method of administration

Research participants will be randomized 1:1 to receive the experimental drug or PLACEBO. Each dose of AZVUDINE or PLACEBO will be administered by the research team member qualified and delegated for this role. The administration, date and time,

will be entered into the study eCRF.

AZVUDINE will be administered orally at a dose of 5mg, once daily, while the participant is hospitalized, for up to 14 days of the full course or until two consecutive negative viral load tests are obtained by RT-PCR.

Research participants will be instructed to swallow the medication whole, without breaking, chewing, crushing, or dividing it before ingesting it. The dose should preferably be given at the same time to all participants every day.

The corresponding PLACEBO will be given in equal amounts, at the same time and respecting the same guidelines described above, to the participants who are allocated to the PLACEBO group.

According to the "Clinical study on the postprandial pharmacokinetics of AZVUDINE" (GQ-FNC-105, Henan Genuine Biotechnology Co, Ltd.), conducted in Beijing You'an Hospital affiliated with Capital Medical University, under the responsibility of Wang Meixia, in the period from Dec-2015 to May-2018, which is one of the reference of studies related to the preliminary stages of clinical development of AZVUDINE (FNC), the incidence of adverse effects observed in both regimens of experimental drug administration (fasting X postprandial) were similar to each other. All AEs were grade 1 in intensity, requiring no therapeutic management measures, thus demonstrating that the FNC administration regimen does not compromise the safety and tolerability of the investigational drug in healthy subjects.

The experimental drug should be administered daily at 8 pm to minimize the risk of falling, since in preliminary studies, it was observed that vertigo (incidence ≥5%) appears to be an AE likely related to FNC. However, no restrictions on meals.

The total course should not exceed 14 calendar days, even if a dose has been missed. The **lost** investigational product, if it occurs, should follow the principle of "whether the lost medication schedule exceeds 1/2 medication interval or not." If the missed medication schedule is within the first 12 hours of the interval between two medications, the missed investigational product can be re-administered normally and

the next administration can be performed according to the original schedule. If the time of the missed medication is more than the first 12 hours of the two-medication interval, the missed investigational product should not be re-administered and the next administration will be advanced.

If a participant is no longer under hospital care, the administration of AZVUDINE will be discontinued.

6.3. DOSE CHANGES

There are no clinical safety or pharmacokinetic data available for AZVUDINE in patients with renal and/or hepatic impairment. Given the benefit-risk ratio in COVID-19 patients, these participants are excluded from the study.

If the glomerular filtration rate (eGFR) decreases to <25mL/min, administration of the drug should not be given that day. The investigational product can be resumed the next day if the eGFR returns to ≥30 mL/min. If the participant's renal function worsens to the point of requiring hemodialysis or hemofiltration, administration of the drug should be discontinued.

If ALT and/or AST increase to >5 times above normal, the dose of AZVUDINE should be stopped and not restarted until ALT and AST are ≤5 times the upper limits of normal.

6.3.1. **Overdosage**

AZVUDINE has undergone preclinical studies to identify adverse events related to long-term use of the drug before it is used in patients with HIV (first use of AZVUDINE). In these preclinical tests, long-term exposure to the drug demonstrated that observable adverse events appear when the dose is exceeded by 20-30 times, but because the tests were done for much longer than suggested, AZVUDINE was shown to be safe at doses of 5mg/day, with the maximum dose over 14 days being 70mg in total.

Animal / human equivalent dose	Animal / Administration time	Total dose used in pre-clinical tests over time
**3.72mg**	1 month	116.6mg
**5.60mg**	3 months	504.0mg
**3.36mg**	6.5 months (26 weeks)	655.2mg
**3.72mg**	9.75 months (39 weeks)	1,088.1mg

Table 7: Relationship between dose and administration time

6.4. DRUG MANAGEMENT AND RECOVERY

The Principal Investigator, or someone delegated by him/her, must check the following items each time he/she receives the study drugs (investigational drug and PLACEBO): drug name; quantity; package specification; drug lot number; whether it complies with the quality inspection report; expiration date; whether the conditions during transport comply with the storage conditions; check that the outer packaging of the drug is in good condition; whether the package label is clear; check that the drug labels are well attached; etc.

If storage conditions do not meet the specified requirements (airtight at room temperature) during transport, the batch of trial drugs should be stored under the original storage conditions, segregated from other stored products, and reported to the Principal Investigator, who, together with the sponsor, should judge whether the received batch may be used or not.

After the transfer sheet is completed, it must be properly saved and filed in the folder after the trial.

The Principal Investigator, or someone delegated by him/her, must keep accurate and complete records, including the receipt, accounting, dispensing,

administration, retrieval, return, or loss of the investigational drug. The Principal Investigator must ensure that all experimental drugs are used only by the participants in this study, and that dosage and use must be in accordance with the research protocol.

Unused experimental drugs must be returned to the sponsor or disposed of by incineration, according to SMS Ordinance No. 028, dated October 9, 2014, using the Drug Destruction Form, and must not be transferred to any other research participant.

Regardless of the form of disposal, the documentation generated by the chosen process must be archived along with the documents generated during the research so that they can be audited.

Trial medications should be checked on a regular basis. In case of non-compliance events (wrong code, loss, lost medications, damage, signs of loss of quality, etc.), this should be recorded and reported immediately to the Principal Investigator and the sponsor.

6.5. DRUG INTERACTIONS

Throughout the trial period, the Principal Investigator and his team must record information about the associated diseases and any therapeutic interventions (including drug therapy), surgical procedures etc. relating to the research participants. If possible, this should also include the diagnosis and date of onset of all diseases and the date of symptom relief, as well as the name of the drug involved, the date the drug was taken, and a description of the operation, etc.

6.6. CONCOMITTANT THERAPIES AND PROHIBITED MEDICATIONS

No clinical drug interaction studies have been conducted between AZVUDINE and other experimental anti-SARS-CoV agents. However, in the exploratory studies conducted in China, patients were medicated with experimental anti-SARS-CoV drugs such as arbidol, lopinavir/ritonavir, interferon-[illegible], hydroxychloroquine, osetalmivir, ribavirin, and others, and no AEs occurred and none were attributed to drug

interaction. Clinical trials with HIV-positive patients (GQ-FNC-103 and GC-FNC-104) showed that multiple doses of EFV and TDF significantly increased the degree of FNC exposure in the body. However, the sample size was small and not clinically relevant, so no dose adjustment was needed for FNC combined with EFV or TDF. In addition, another clinical trial (GQ-FNC-201) showed that there was no drug interaction with marketed antivirals for HIV treatment (TDF and EFV), and that the therapeutic effect was comparable to the currently recommended first-line treatment regimen: 3TC + TDF + EFV. All AEs reported in these trials were grade 1 and no additional treatment was required.

The use of some concomitant therapies may interfere with the efficacy, safety, and clarity of the data obtained from the research. There is no evidence that administering AZVUDINE simultaneously with other agents may have no effect or adverse effects, but the information will be added in the protocol.

The Principal Investigator should still review medications prescribed for pre-existing comorbidities and evaluate whether these agents may lead to antagonism or synergy with AZVUDINE or jeopardize the safety of the participant, and should modify safety monitoring at his or her discretion if deemed necessary.

It is recommended that individuals who are taking another antiviral for a concurrent infection (e.g., oseltamivir for an influenza virus, lopinavir/ritonavir for HIV, etc.) or immunosuppressive medications for other medical conditions (tocilizumab for rheumatoid arthritis, hydroxychloroquine for lupus, etc.), or experimental medications for COVID-19 not be included in this study. Individuals on alternative treatment should begin treatment with AZVUDINE after the washout period described for each medication:

MEDICINE	*WASHOUT*
Oseltamivir	>3 days
Lopinavir	7 days
Ritonavir	7 days

Tocilizumab	6 weeks
Hydroxychloroquine	14 days

Table 8: Drug washout period

It is planned to collect information on concomitant medications at visits 1 through 17. Concomitant medications should be reported in the appropriate eCRF. Report all prescription medications taken during the study period.

Final guidance on AZVUDINE and possible drug interactions, if available, will be updated in the Investigator's Brochure and in protocol amendments.

6.7. PARTICIPANT MEMBERSHIP

Trial medication adherence is defined as the ratio of the number of actual medications taken to the number of medications that should have been taken during the dose administration period, multiplied by 100. Investigators should record the specific reasons for participants' inadequate adherence in the original medical records and in the eCRF for each visit. The definition of experimental medication adherence failure is: the proportion is less than 80% or greater than 120%.

$$\text{Adhesion evaluation (\%)} = \frac{\text{Actual amount of drug used}}{\text{intended quantity of the drug used}} \times 100$$

7. EVALUATIONS AND VISITATION SCHEDULE

Participants will preferably be evaluated by the same evaluator at the beginning of the study (screening and randomization: D-1 and D0 respectively).

During follow-up, including the assessment of participants who were not discharged after the end of treatment (D21) and at two visits after discharge: two weeks (D28) and sixty days (D60) after the end of treatment.

Table 39 shows the tests to be used to assess eligibility criteria and to monitor safety and efficacy during the study period.

The coronavirus viral load will be detected every 2 days. The treatment will

last a maximum of 14 days, and can be stopped early if two consecutive negative RT-PCR tests are obtained, or if the participant decides not to continue in the study.

During the course of treatment, if the results of safety and efficacy testing show changes of concern, the investigators must determine whether or not the participant should continue with treatment. The Principal Investigator may adjust the procedures performed during the hospital stay according to the research participant's condition and the results of clinical examinations. The schedule of study procedures and examinations is detailed in Table 9.

Item	Requirements and outcome measures
Demographic Information	Nationality, age, sex, height and weight
Anamnesis	Past/current medical history (history of illness, history of operation), history of anaphylaxis, medication use, alcohol use, drug abuse, participation in clinical trials, surgical treatment, or pregnancy/lactation.
Vital signs	Body temperature, heart rate, respiration, blood pressure
DIGITAL OXIMETRY	-
Blood glucose	Blood glucose level
Detection of viral load by RT-PCR	Sample of airway epithelium
ECG	12-lead or 18-lead ECG
CT of the lung	-
CBC	RBC, Hb, HCT, MCV, MCH, MCHC, WBC, NEUT #, LYMPH #, MONO #, EOS #, BASO #, PLT
Biochemical Examinations	AST, ALP, Cholinesterases, ALT, Alb, TP, TBIL, DBIL, GGT, Creatinine, UREA, UA, K^+, Na^+, Cl^-, Lactate, Ca^+, P.
Taxa de sedimentação de eritrócitos (ESR)	ESR
C-reactive protein (CRP)	-

Cardiac markers and enzymes	Troponin I, BNP, CK, CK-MB
Septicemia	PCT
Blood Lactic Acid	Lactic Acid
Interleukin-6	IL-6
Coagulation test	TP, TTPA, FIB, TT
Immunological markers	IgA, IgG, IgM, complement C3 and C4
Infectious Diseases	Ab Hepatitis A, Ag Hepatitis B, Ab Hepatitis C, Ab HIV, AbTreponema *pallidum*
Respiratory parasites	*Legionella pneumophila*, *Mycoplasma pneumoniae*, *Coxiella burnetii* (Q-Rickettsia), *Chlamydia pneumoniae*, adenoviruses, respiratory syncytial virus, influenza A virus, influenza B virus, Parainfluenza virus.
Triple rapid test for respiratory parasites	Respiratory syncytial virus, adenovirus, *Mycoplasma pneumoniae*
T-lymphocyte subsets	CD4+ and CD8+ T-cell count
Pregnancy Test	Applicable to female participants

Table 9: Parameters for safety and efficacy evaluation

7.1. ASSESSMENT OF EFFECTIVENESS

7.1.1. Primary Efficacy Evaluation (Objective)

- Proportion of participants with clinical status improvement [Period: until D15, approximately 14 days].
    - The criterion for a participant to have an improvement in clinical status is a decrease on the WHO Clinical Improvement Ordinal Scale in at least one category compared to screening (WHO, Jun/2020).

7.1.2. Secondary Efficacy Evaluation (Endpoints)

- Proportion of participants with a clinical outcome of cure during the study; [Period: until D15, D21, and D28, approximately 14-30 days].
    - The clinical endpoint of cure is defined in this protocol as the absence of viral RNA in collected samples and clinical conditions for hospital discharge[14] .
- Time to normalization of body temperature (below 37.6℃ axillary); [Period: until D15, D21 and D28, approximately 14-30 days]
- Tempo de melhora da diarréia, mialgia, fadiga, mal-estar, tosse, dispnéia e cefaléia; [Período: até o D15, D21 e D28, aproximadamente 14-30 dias]
- Changes in liver and kidney function *baselines*, and inflammatory and immunological markers: [Period: until D15, D21 and D28, approximately 14-30 days]
    - Lab tests performed on days D1, D3, D5, D7, D9, D13, D15, D21, D28, and D60.
- Evaluation of the time to negative conversion of the SARS-CoV-2 viral load by RT-PCR between AZVUDINE (FNC) group and control group; [Period: until D15, D21 and D28, approximately 14-30 days].
- Evaluation of the number of cycles for the detection of SARS-CoV-2 viral load by RT-PCR and application of the standard curve for viral load calculation; [Period: until D15, D21 and D28, approximately 14-30 days].

[14] According to the WHO Working Group on Characterization and Clinical Management of COVID-19 Infection (July, 2020), clinical and virological absence of infection (non-detectable RT-PCR) is suggestive of CURE for initially infected participants.

- Analysis of the relationship between the calculated viral load and the clinical progress of participants in the AZVUDINE (FNC) group compared to the control group; [Period: until D15, D21 and D28, approximately 14-30 days].
- Time and proportion of lung imaging improvement during treatment (Pattern of ground-glass opacities, mosaic paving, alveolar consolidation, reticular pattern/septal thickening, opacity with inverted halo, pleural/pericardial effusion,

fibrosis and/or lymphadenomegaly); [Period: until D15, D21 and D28, approximately 14-30 days].

- Time of improvement and proportion of respiratory symptoms and signs during treatment (Lung rales, cough, sputum, sore throat); [Period: until D15, D21, and D28, approximately 14-30 days].
- Time to normalization of O_2 saturation (above 95%); [Period: until D15, D21 and D28, approximately 14-30 days].
- Time to normalization of respiratory rate to levels ≤ 24rpm, in room air; [Period: until D15, D21 and D28, approximately 14-30 days]
- Frequency of supplemental oxygenation or non-invasive ventilation; [Period: until D15, D21 and D28, approximately 14-30 days].
- Frequency of invasive mechanical ventilation or extracorporeal membrane oxygenation (ECMO); [Period: until D15, D21, and D28, approximately 14-30 days].
- Proportion of moderate cases that progressed to severe cases requiring intensive care unit (ICU) care; [Period: until D15, D21, and D28, approximately 14-30 days].
- Length of hospital stay; [Period: until D15, D21 and D28, approximately 14-30 days].
- Occurrence of drug interactions; [Period: until D15, D21 and D28, approximately 14-30 days].
- Frequency and intensity of adverse events; [Period: until D15, D21 and D28, approximately 14-30 days].
- Frequency and intensity of unexpected adverse events; [Period: until D15, D21 and D28, approximately 14-30 days].
- Frequency and intensity of serious adverse events; [Period: until D15, D21 and D28, approximately 14-30 days].
- Taxa de mortalidade por todas as causas durante o estudo; [Período: até o D15, D21 e D28, aproximadamente 14-30 dias]
- Evaluate the tolerability of AZVUDINE (FNC) use at 5mg/day regimen for up to 14 days: [Period: until D15, approximately 14 days].
    - Calculating treatment adherence;

- Total time of use of AZVUDINE (FNC);
- Proportion of participants who withdrew consent;
- Proportion of participants who dropped out of treatment.

7.2. SAFETY ASSESSMENT

Any clinical manifestations, severity, time of occurrence, duration, treatment methods, and prognosis must be recorded in the participant's eCRF. The following safety parameters will be evaluated:

- Frequency and intensity of adverse events;
- Frequency and intensity of unexpected adverse events;
- Frequency and intensity of serious adverse events;
- Adverse events related to the experimental drug;
- Abnormal laboratory test values;
-
    - Routine blood work (WBC count, RBC count, Hemoglobin, Platelet count, Neutrophil count, Lymphocyte count), ESR, IL-6, C-reactive protein, Liver function (AST/TGO, ALT/TGP, GGT, BT, ALP/FA), renal function (GFR, serum creatinine), coagulation function (APTT, TAP/PT, TT, FIB), blood lactate, cardiac enzymes and markers, procalcitonin, T lymphocyte subsets;
- Vital signs (body temperature, heart rate, respiration, blood pressure);
- Pregnancy test for women of childbearing age (blood);
- ECG.

Clinical safety will be assessed through spontaneous participant reports or direct observation by the Principal Investigator or his/her team on a daily basis, or by asking participants about adverse events on a non-induced basis. Participants will be screened with blood tests, imaging tests, respiratory tract tests, and vital signs, as listed in Table 39, as indicated, but not limited to these items.

7.3. VISIT SCHEDULE

The research center is responsible for defining the strategies for recruitment and initial screening of research participants, knowing that the screening process can be suspended at any time if exclusions are identified by the study team.

VISIT 1: INTIAL (D-1)

Patients with moderate to severe symptoms for COVID-19, hospitalized or with indication for hospitalization, aged over 18 years, will be invited to participate in the study. **If the volunteer accepts to be a participant**, he (she) and his (her) companion, if applicable, will be informed of the study procedures, including laboratory tests in the screening period, the criteria for inclusion and exclusion from the study, as well as his (her) rights and duties. Only after signing the informed consent form, the patient will be included in the study and the eligibility criteria can be explored after the anamnesis, laboratory tests, nasopharyngeal and oropharyngeal swab specimen collection for RT-

PCR testing, and imaging tests. **Confirmation of study participant eligibility will be assessed once all screening data are available.**

The following procedures will be performed in visit V1:

- Invitation to volunteer;
- Consent process and signing of the TCLE;
- Verification of inclusion and exclusion criteria;
- Demographic data collection;
- Anamnesis;
- Collection of medical history data;
- Vital signs monitoring;
- Collect blood and respiratory samples (nasal and oropharyngeal swabs) for screening tests;

- Collecting blood for a pregnancy test (women of childbearing age).
- Eligibility assessment and confirmation with the Principal Investigator.

VISIT 2: RANDOMIZATION (D0)

. After confirmation of eligibility for the study by meeting the inclusion and exclusion criteria and performing the screening procedures, eligible individuals will be randomized to the experimental or control group and then transferred to Santa Casa de Misericórdia de Campos for hospitalization in a ward/apartment, and will follow the Treatment Protocol for COVID-19 - 2nd Version (July 02, 2020) of Santa Casa de Misericórdia de Campos.

The following procedures will be performed in visit V2:

- Validation of eligibility by the Principal Investigator;
- Randomization of research participants;
- Admission or transfer of the participant to the apartment or infirmary of Santa Casa de Misericórdia de Campos;
- Dispensing of the experimental drug;
- Accounting for the experimental drug.

VISIT 3-16: PROCEDURE (D1-D14)

The treatment period will start on D1 according to randomization.

The treatment will last up to 14 days, and may be stopped early in case of treatment success (2 consecutive negative RT-PCR tests), participant dropout, or at the Principal Investigator's option, if deemed necessary for the safety of the participant.

For the **Early Termination (ETA)** of treatment, participants should follow the routine from visit 17: End of Treatment (D15), in advance.

The Principal Investigator for the study will be available to answer any questions.

The experimental drug should be administered daily at 8 pm to minimize the risk of falling, since in preliminary studies, it was observed that vertigo (incidence ≥5%) appears to be an AE likely related to FNC. However, no restrictions on meals.

The following procedures will be performed in the V3-16 visits:

- Administration AZVUDINE or PLACEBO daily, at the same time at 8pm, to minimize the risk of falling, since in preliminary studies, it was observed that vertigo (incidence ≥5%) may be an AE likely related to FNC;
- Monitoring drug interactions and AEs on a daily basis;
- Monitoring symptoms, vital signs, oximetry and blood glucose daily;
- Collection of blood samples and respiratory sample (nasal and oropharyngeal swab) for safety and efficacy monitoring tests every 48h);
- CT scan of the lung every 4 days and electrocardiogram on days D1 and D11;

The Principal Investigator may adjust the procedures performed during treatment according to the condition of the research participant and the results of clinical examinations.

VISIT 17: END OF TREATMENT (D15)

At the end of treatment or discontinuation of the investigational drug or PLACEBO, all participants will be required to undergo procedures and tests for safety and efficacy monitoring. The procedures should be performed before discharge from the hospital approximately 24 hours after the administration of the last dose of the investigational drug or PLACEBO, because for the 5 mg dose of AZVUDINE, the mean half- life is 13.8 hours, and it is excreted in the urine within 24 hours.

The treatment will last up to 14 days, and may be stopped early in case of

treatment success (2 consecutive negative RT-PCR tests), participant dropout, or at the Principal Investigator's option, if deemed necessary for the safety of the participant.

The following procedures will be performed at the V17 visit:

- Monitoring drug interactions and AEs;
- Symptom monitoring, vital signs, oxygen and blood glucose measurement;
- Collection of blood samples and respiratory sample (nasal and oropharyngeal
- swab) for safety and efficacy monitoring tests;
- CT scan of the lung and electrocardiogram.
- Collecting blood for a pregnancy test (women of childbearing age).

VISIT 18: FOLLOW-UP (D21)

Participants who, even **after the end of treatment, remain in the hospital must undergo the procedures and tests for safety and efficacy monitoring that are** scheduled to occur approximately **7 days after the administration of the last dose of** the investigational drug or PLACEBO.

The following procedures will be performed at the V18 visit:

- Monitoring drug interactions and AEs;
- Symptom monitoring, vital signs, oxygen and blood glucose measurement;
- Collection of blood samples and respiratory sample (nasal and oropharyngeal
- swab) for safety and efficacy monitoring tests;
- CT scan of the lung and electrocardiogram;

Participants who are not discharged from the hospital after the end of treatment will be required to follow the follow-up schedule on D28 and D60. However, safety procedures and examinations will not be exclusive to these days and research participants who still remain hospitalized will be assisted at all times by the Principal

Investigator and his team.

VISIT 19: FOLLOW-UP (D28)

Two weeks after discharge from the hospital, the volunteers will return to the research center for procedures and tests to monitor safety and efficacy after the end of treatment.

The following procedures will be performed at the V19 visit:

- Symptom monitoring, vital signs, oxygen and blood glucose measurement;
- CT scan of the lung and electrocardiogram.

The Principal Investigator may adjust the procedures performed during the hospital visit according to the condition of the research participant and the results of the clinical examinations.

VISIT 20: FOLLOW-UP (D60)

60 days after discharge from the hospital, the volunteers will return to the research center to undergo procedures and tests to monitor safety and efficacy.

The following procedures will be performed in visit V20:

- Symptom monitoring, vital signs, oxygen and blood glucose measurement.
- Collecting blood for a pregnancy test (women of childbearing age).

The Principal Investigator may order tests according to the participants' previous results.

Schedule of visits and procedures

Week/Day	D-1	D0	D1	D2	D3	D4	D5	D6	D7	D8	D9	D10	D11	D12	D13	D14	D15 after the start of treatment	D21[5] after the start of treatment	D28 after start of treatment	D60 after the start of treatment[3]
Visit	1	2	3	4	5	6	7	8	9	10	11	12	13	14	15	16	17	18	19	20
Interval of the visit	± 4 days	-	-	-	-	-	-	-	-	-	-	-	-	-	-	-	Antes da alta hospitalar	± 3 dias	± 3 dias	± 3 dias
STAGES	TREAT	RANDOMIZATION	BLINDED TREATMENT[1]														END OF TREATMENT[2]	FOLLOW-UP		
TCLE	X																			
DEMOGRAPHIC INFORMATION	X																			
ANAMNESIS	X																			
ELIGIBILITY CRITERIA	X																			
RANDOMIZATION		X																		
DISPENSING OF THE MEDICATION		X																		
MEDICATION ACCOUNTING		X																		
SYMPTOM MONITORING	X		X														X	X	X	X
VITAL SIGNS	X		X														X	X	X	X
FINGER OXYGEN PULSE	X		X														X	X	X	X
BLOOD GLUCOSE			X														X	X	X	X
PREGNANCY EXAM[4]	X																X			X

CT LUNG			X		X				X				X				X	X	X	
ECG			X										X				X	X	X	
RT-PCR	X		X		X		X		X		X		X		X		X	X	X	
BIOCHEMICAL EXAMINATIONS	X		X		X		X		X		X		X		X		X	X	X	
T-LYMPHOCYTES			X		X		X		X		X		X		X		X	X	X	
CBC			X		X		X		X		X		X		X		X	X	X	
FUNCTION OF COAGULATION			X														X	X	X	
ESR/VHS			X						X								X	X	X	
CARDIAC MARKERS AND ENZYMES			X						X								X	X	X	
C-REACTIVE PROTEIN			X						X								X	X	X	
PROCALCITONIN			X						X								X	X	X	
IL-6			X						X								X	X	X	
PHOSPHORUS IN URINE			X														X	X	X	
IMMUNOLOGICAL MARKERS			X															X	X	
INFECTIOUS DISEASES			X																	
RESPIRATORY PARASITES			X																	
TRIPLE TEST FOR RESPIRATORY PARASITES			X															X	X	
AZVUDINE OR PLACEBO ADMINISTRATION										X										

MONITORING DRUG INTERACTIONS			X	X				
AE MONITORING			X	X				

Tabela 10: Cronograma de visitas e procedimentos do estudo.

[1] *Day 1 of administration will be recorded as day 1 (D1) and the treatment days follow until day 14 (D14). The Principal Investigator may adjust the procedures performed during treatment according to the condition of the research participant and the results of clinical examinations (the condition manifests itself more rapidly in some patients and these patients should be observed closely).*

[2] *The examinations and procedures under visit 17 (D15) must be performed by all participants as soon as the treatment is terminated, including participants who have stopped treatment early (AT) and for any other reason.*

[3] *The. procedures and examinations planned to be performed at visit D28 and D60 will be determined by the Principal Investigator according to the participants' previous examinations.*

[4] *For women of childbearing age only.*

[5] *Visit 18 procedures will be performed only on research participants who are still hospitalized.*

7.4. EXTRA VISITS

In addition to the visits provided here, **extra visits may be made to** perform procedures indicated by the Principal Investigator prioritizing the safety of the visiting participant.

7.5. VIEWING WINDOW

To be eligible to participate in the study, among the proposed inclusion criteria is the presence of a positive diagnostic test for SARS-CoV-2 by molecular amplification of the virus in a respiratory sample (nasopharyngeal, oropharyngeal, lower respiratory tract, e.g.: sputum) collected <96 hours before randomization. For this reason, the time between screening and randomization should not exceed 4 days.

To attend the end-of-follow-up visits (**D28 and D60**) participants will have the possibility to **advance or delay by up to three days** from the initially scheduled date.

8. ADVERSE EVENT REPORTING

Adverse Event (AE), according to ANVISA, means "any adverse medical occurrence in a patient or clinical trial participant to whom a pharmaceutical product has been administered and that does not necessarily have a causal relationship to the treatment. As a result, an AE can be any unfavorable and unintended sign, symptom, or disease (including results outside the reference range), associated with the use of a product under investigation, whether related to it or not." The adverse event can be related to the following situations:

- Exacerbation of pre-existing medical conditions and/or the original disease (including symptoms, signs, laboratory abnormalities);
- Any new adverse events or new adverse medical conditions (including symptoms, signs, newly diagnosed diseases);
- Abnormal values of clinically significant laboratory or imaging tests or results that are not caused by concomitant diseases.

8.1. SERIOUS ADVERSE EVENT (EAG)

According to ANVISA's definition, a serious adverse event is "one that results in any drug experience [...], occurring at any dose and resulting in any of the following outcomes."

- Death;
- Imminent threat to life;
- Persistent or significant disability/disability;
- Inpatient hospitalization or prolonged hospitalization;
- Congenital anomaly or birth defect;
- Any suspected transmission of an infectious agent through a drug;
- Other clinically significant events: events that can lead to one of the above, including but not limited to the following events, such as allergic bronchospasm requiring intensive treatment in the emergency room or at home, blood system cachexia or seizures not requiring hospitalization, potential drug-induced liver injury, spread of pathogens (non-pathogenic or pathogenic) via the study drugs, pregnancy, overdose, secondary tumors, etc, are generally considered serious, although they may not lead to death or hospitalization.

The Principal Investigator should provide the EAG severity criteria, based on the study protocol and their own medical judgment, on the eCRF and the results of the evaluation of the expected occurrence of the EAG and the correlation (cause and effect) with the study drug.

If the causality assessment performed by the Principal Investigator is missing or cannot be obtained, the sponsor will make the judgment and provide the final assessment, which will subsequently be confirmed by the Principal Investigator. If the Principal Investigator cannot determine whether or not the AE belongs to an SAE, then it will be considered an SAE until its nature is proven.

When any SAE occurs in the study, whether related to the experimental drug or not, the investigators should provide immediate assistance.

Planned or elective hospital admissions, for administrative reasons (such as annual physical exam) or prolongation of length of stay unrelated to worsening AEs, will not be considered SAEs. **The following situations will not be reported as SAE:**

- Treatments/procedures for a pre-existing condition/illness reported in the screening period;
- Hospital readmission due to the original disease with no new AE and no exacerbation of the original disease (such as verification of laboratory abnormalities that have persisted so far);
- Invasive (such as surgery) and non-invasive diagnostic or therapeutic procedures should not be reported as an EAG. However, it should be reported when the condition that led to that operation meets the definition of AE. For example, acute appendicitis that occurred during the AE monitoring period should be reported as an AE and therefore an appendectomy should be recorded as the treatment method for this AE.
- AEs leading to hospitalization or prolonged hospitalization in clinical trials should be considered as AEs. The following inpatient situations are not considered as AEs: rehabilitation facilities; nursing home; routine emergency room admission; same day surgery (as outpatient/same day/outpatient surgery).

Hospitalization due to symptoms and signs of disease progression during the trial should not be reported as an SAE, while death due to disease progression (including signs and symptoms of progression) should be reported as an EAG. Events that caused death should be reported as an EAG, for example, if cancer led to the participant's death during the trial.

8.2. SUSPECTED UNEXPECTED SERIOUS ADVERSE EVENT (SUSAR)

Unexpected serious adverse reaction whose nature, severity, consequence or frequency is not consistent with the expected risks described by the information in the most current investigator's brochure. All unexpected serious adverse reactions occurring during the clinical trial that are definitely related to or suspected of the investigational product are SUSAR. The assessment of SUSAR should be performed considering the following aspects:

- If the AEs are listed in the investigator's brochure (BI);
- Although the AEs are listed in the BI, whether or not their severity is consistent with what is described in the BI;
- Although the adverse events are listed in the BI, the frequency of occurrence is higher than described in the BI;
- Other situations.

.

8.3. ADVERSE EVENT OF SPECIAL INTEREST

According to the results of the current clinical trials, the adverse events that may be related to the product are:

- Dizziness;
- Headache;
- Decreased phosphorus in the blood;
- Increased alanine aminotransferase;
- Increased aspartate aminotransferase;
- Increased total bilirubin;
- Increased γ-glutamyl transferase;
- Decreased neutrophils;
- Increased glucose in the blood.
-

<u>However, according to the AE incidence data reported in the clinical</u>

development phases of the FNC performed earlier, among the AEs already described, AEs of special interest will be considered:

- Changes in markers of liver function [15]:
    - Elevation of AST/TGO above reference values.
    - Elevation of ALT/GTP above reference values.
- Changes in markers of renal function:

  Decrease in estimated Glomerular Filtration Rate (eGFR) <30mL/min.
- Grade 3 AEs related to dizziness, due to increased risk of falling.

The Principal Investigator should classify the degree of intensity and correlation (cause and effect) between the test drug and the AE of Special Interest, based on the trial protocol and his own medical judgment, and record it in the specific eCRF. The sponsor should be notified within 7 days about the occurrence of AE of Special Interest.

8.4. AES MONITORING

If there is any abnormality or AE, serious or not, unexpected or not, during or after treatment, follow-up should be made until the clear outcome of recovery or stable status. Individuals with AEs that develop after discharge should contact the Principal Investigator or his/her team to determine what action should be taken for that individual to fully recover from the AE.

[15] TGO Adult 12 to 46 U/L; TGP Adult 03 to 50 U/L (Source: Hermes Pardini Institute)

After the end of the trial, if participants have AEs again that are or could be related to the use of the investigational drug, the PI should inform the sponsor in time.

For all EAGs (including those still in the developmental stage after the end of the trial and until the end of the last patient's follow-up), the Principal Investigator should follow the patient until recovery, stabilization, or death to ensure that all

problems are resolved. Until the EAG has been resolved, the Principal Investigator should keep the sponsor informed. Detailed follow-up information should be provided, such as whether or not special treatment is needed after the trial, whether or not hospitalization is required, etc.

8.5. AE INTENSITY

The intensity of AE in this study is classified according to the World Health Organization criteria, adopted by ANVISA:

- **Mild:** present less than 25% of the time, with an intensity that a person can tolerate and that has rarely happened since the start of treatment.
- **Moderate:** present less than 50% of the time, with an intensity that interferes with the individual's day-to-day activities and has happened occasionally since the beginning of treatment.
- **Severe:** present more than 50% of the time, with an intensity that partially alters the individual's daily life and has happened frequently since the beginning of treatment.
- **Complete impairment:** present more than 95% of the time, with an intensity that completely alters the individual's daily life, and has occurred every day since the start of treatment.
- **Not specified:** not enough information to specify the intensity.
- **Not applicable:** it is inappropriate to use a gradation (e.g. menstrual functions).

8.6. CASUALITY BETWEEN EAS AND DRUGS IN THE STUDY

All AEs, whether serious or not, unexpected or not, should be reviewed by the Principal Investigator for correlation analysis with study drugs, in accordance with 11. The following factors should be taken into consideration when analyzing the correlation between AEs and study drugs:

- If there is a reasonable time sequence between the time of action of the

drug and the AEs, and it was possible to observe a plausible relationship between the administration of the drug and the event that occurred;

- Whether AEs can be explained by the original disease, by another intervention, or by environmental factors;
- Whether there was a response, pharmacological or pathological, after discontinuation or dose reduction of the study drug;
- If the same AE reappeared after resumption of the suspected drug, whether re-exposure of the participant to the study drug was possible;
- Whether the clinical or pathological manifestation of the AE is consistent with knowledge of the pharmacological and toxicological classification of the known study drug;
- If the data about the EA is clear, complete and traceable.

CORRELATION	CRITERIA
**Sure/Defined**	• There is a reasonable time sequence between the AE and thedrug;<br>• EA cannot be explained by other factors;<br>• There was a response after dose withdrawal or reduction;<br>• The same AE reappeared after resumption of the drug, if re-exposure was possible;<br>• The AE is consistent with the medicine.
**Probable**	• There is a reasonable time sequence between the AE and thedrug;<br>• Unlikely that the AE is attributable to other factors;<br>• There was a response after dose withdrawal or reduction;<br>• No re-exposure of the drug was possible or there is no clear information.

**Possible**	• There is a reasonable time sequence between the AE and the drug;<br>• AE can also be attributed to other factors;<br>• There is no clear information about the response after dose withdrawal or reduction.
**Unlikely**	• There is no clear sequence of reasonable time between the EA and the drug;<br>• AE can also be attributed to other factors;
**Conditional/Unclassified**	• There is no time sequence between the AE and the drug;<br>• The data is not clear for;<br>• Additional data is under investigation
**Inaccessible/Unclassifiable**	• The narrative of the account suggests an EA;<br>• The information is insufficient or contradictory;<br>• The data cannot be supplemented or verified.

Table 11: Causality relationship between AEs and study drugs.

8.7. AE COLLECTION AND REGISTRATION

Any information relevant to the trial should be recorded from the first administration to the end of follow-up. AEs will be monitored throughout the study and the Principal Investigator, or someone deligated by him, will be responsible for recording all adverse medical events observed during the study, in detail, in the source documents and in each participant's eCRF, during the study period from the first dose to the end of follow-up, regardless of severity and causal relationship to the study drug.

All SAEs must also be recorded in eCRF as soon as they occur, and the information provided in the SAE report form must be consistent with the event-related data that is recorded in eCRF. All AEs, SAEs and special interest AEs must include the following content in detail:

- **Description:** Events should be described in medical terms, rather than the words used to record the participants' reports in the original document. If

applicable, symptoms, signs, laboratory abnormalities, and diagnosis should be included. If the same AE occurs more than once, in the same participant, and the participant recovered from both events, the AE should be recorded twice.

- **Date of occurrence**: the date when the AE first occurred in the participant or the date when the symptoms related to the AE occurred should be recorded. If the AE is the test result of an abnormal laboratory test or test with clinical significance, the date of occurrence is the date the test was performed.
- **Date of result:** the date of resolution of the AE-related symptoms should be recorded.
- **AE intensity and severity:** classified as: (1) mild, 2 (moderate), 3 (severe) or 4 (complete/severe impairment). [16]
- **Causal relationship between AE and study drugs:** as per Table 5.
- **Measures for the study drug:** It should be recorded whether the participant continued using the drug or had to stop using it as a result of the AE or SAE. If the reaction occurred after the end of treatment, this item does not apply.
- **Measures for AE:** It should be recorded what measures were taken to solve the AE, such as drug therapy and/or non-drug therapy, hospitalization, surgery, among others. If no action was taken, this fact should be reported.
- **Outcome:** the disappearance of signs or symptoms; recovery with sequelae; stability; worsening; death; or loss to follow-up must be recorded.
- **Significant AE:** refers to any obvious AE, hematologic, or other laboratory test abnormality that needs targeted clinical interventions (such as drug withdrawal, dose reduction, and/or routine treatment), except for SAE.

If an AE or SAE occurs during the trial, regardless of whether it is related to the study drug or not, the investigators should immediately take appropriate treatment measures to ensure the safety of the participants.

[16] Manual for adverse event reporting and safety monitoring in clinical trials / Brasília. Anvisa, 2016

8.8. EVALUATION OF ABNORMALITIES OF CLINICAL LABORATORY TESTS AND OTHER ABNORMALITIES CONSIDERED AS EA

Laboratory test abnormalities without clinical significance are not recorded as AE or SAE. Laboratory test abnormalities with clinical significance (flagged as "abnormalities with clinical significance", such as clinical blood routine, blood biochemistry, etc.) and other abnormalities (such as ECG, vital signs), that meet the definition of AE or EAG, upon the principal investigator's evaluation and judgment, or that require individuals to receive specialized routine treatment, should be recorded as AE or SAE.

If laboratory abnormalities are part of the syndrome, record the results of the syndrome or diagnosis (e.g., anemia) rather than the results of the laboratory tests (i.e., reduced hemoglobin).

8.9. PREGNANCY

If a female participant or the spouse of a male participant becomes pregnant during the trial, this is recorded and reported to the ethical and regulatory authorities and the sponsor. If the pregnancy is discovered during the treatment period, the use of the trial drug should be discontinued and the pregnancy event should be reported in the eCRF. If the pregnancy is discovered during the follow-up period, it should also be reported in the eCRF. If the pregnancy occurs within 6 months after the end of the trial, the pregnancy event should be reported to the sponsor and ethical and regulatory authorities. In all cases, the mother and baby should be followed up until delivery. Pregnancy complications and selective termination of pregnancy for medical reasons should be reported as AE or SAE, and spontaneous abortion should be reported as SAE.

8.10. NOTIFICATION OF AE

AEs, SAEs, and SUSARs should be recorded in detail in source documents and in the participant's eCRF, in accordance withMedDRA terminology (version 23.0) to specify the event that occurred.

The Principal Investigator must notify the sponsor of the occurrence of AEs in a timely manner. Non-serious AEs should be reported to the ethical and regulatory authorities in the follow-up reports of the investigational medicinal product.

The Principal Investigator must notify the sponsor of the occurrence of SAE or SUSAR within 24 hours of knowledge of it, in a dated and signed document, regardless of the causal relationship to the trial drug. Additional information should be reported as soon as available. The sponsor must report the occurrence of unexpected, possible, probable or definite SAEs to the regulatory and ethical authorities within 7 calendar days[17].

For SAE or SUSAR whose information is temporarily incomplete and uncertain, it should also be reported in a timely manner, and should be reported in the form of a follow-up report to the regulatory authorities after more information is obtained.

9. CONCLUSION AND DISCONTINUATION OF THE STUDY

Information about the completion or discontinuation of the trial research participant and the reason for study discontinuation will be recorded in the appropriate CRF.

9.1. CONCLUSION OF THE PARTICIPANT

At the end of the treatment period of up to 14 days or when the coronavirus viral load detection by RT-PCR is negative on two consecutive occasions, considering a difference of at least 24 hours between the two sample collections, the study medication

[17] https://formsus.datasus.gov.br/site/formulario.php?id_aplicacao=3961

will be discontinued and the participant continues with the standard treatment therapy. Treatment may also be discontinued if the Principal Investigator deems it best for the participant's safety, or if the participant decides not to continue with treatment. In either situation, follow-up will be done on days D28 and D60 after the end of treatment.

The participant will only be discharged from the hospital at the end of treatment after medical evaluation by the Principal Investigator. The participant will be required to return to the hospital for follow-up visits. After the last visit, the subject's participation in the study will be considered terminated.

The need for additional intervention or the occurrence of safety endpoints/endpoints do not constitute trial completion and are not criteria for withdrawal from the trial or the trial medication.

9.2. STUDY CONCLUSION

The study will be considered completed when the last participant completes his or her last visit.

9.3. INTERRUPTION OR DISCONTINUATION OF THE STUDY PARTICIPANT

Participants are free to withdraw consent and/or discontinue participation in the study at any time, without prejudice to their subsequent standard of care. The will of the research participant and the medical care for them must take precedence over any research intent or procedure associated with the study. The research participant's participation in the trial may also be discontinued at any time at the discretion of the investigator or sponsor. Participants can also be discontinued from the trial if the trial is terminated. Thus, the following criteria define research participant discontinuation:

1. Voluntary withdrawal from the participant at any time and without any prejudice to his/her health;
2. Refusal or inability to perform and/or complete study procedures, when applicable;
3. Loss of contact with the participant for follow-up;
4. Death;
5. Closure of the study.

The participant who discontinues the study must complete the safety and efficacy evaluations, despite discontinuation of study medication, scheduled for study termination (V17) listed in the schedule of procedures detailed in Table 10, as close as possible to the participant's last dose of study medication. Data from the visit, including the primary reason for premature discontinuation of the study, will be recorded in the study termination electronic clinical record (eCRF).

If participants withdraw from the study due to subjective motivations, the Principal Investigator should make reasonable efforts to contact the participants, or other contacts provided by them, by phone or email to determine the reasons for the complete withdrawal as accurately as possible. If possible, the Principal Investigator should describe the detailed reasons for withdrawal in the original record and in the eCRF.

9.4. DISCONTINUATION OR INTERRUPTION OF STUDY MEDICATION

If during the course of the study participants develop a more serious condition requiring intubation, considering that the drug is administered orally, the drug will be discontinued.

If during the course of this study participants exhibit any of the following criteria below, the Principal Investigator should judge whether they should discontinue their study medication:

- Confirmed by further examination, TBIL ≥B times the upper limit of normal value, ALT and AST ≥B times the upper limit of normal value;
- If the eGFR decreases to an eGFR <25 mL/min and the participant's renal function worsens to the point of requiring hemodialysis or hemofiltration;
- Any situation that, in the judgment of the Principal Investigator, puts the safety of the participant at risk.

Participants who discontinue study medication early should be encouraged to continue the post-treatment visit to continue safety and efficacy data collection despite discontinuation of study medication. Participants who agree to continue regularly scheduled study visits should complete the study termination assessments (V17) listed in the schedule of procedures detailed in Table 10 as close to the participant's last dose of study medication as possible. The visit data, including the primary reason for discontinuation of study medication, will be recorded in the eCRF. Subsequent study visit data, if applicable, will be recorded in the visit-specific eCRF, and the participant's final study visit will be recorded in the study termination eCRF.

If the reason for participants' discontinuation is an AE, they will continue to receive rigorous medical care until the clear outcome of recovery from AE or steady state. See Section 7 of this protocol for detailed follow-up information. For women who became pregnant during the trial period, the Principal Investigator can refer to item 4.9this protocol for treatment.

Participants discontinuing study medication who are unwilling to continue with regular study visits, but are willing to continue to provide their information for study use will be encouraged to remain in the study. The investigator will contact the sponsor's representative to determine the best approach based on the participant's situation, even if physical and laboratory examinations are not performed, monitoring for drug interactions and adverse events can be considered for such cases.

9.5. LOSS OF FOLLOW-UP

The investigator should make every effort to contact participants who do not return for scheduled visits so that they are not declared as "lost to follow-up".

Participants will be considered "lost to follow-up" only after reasonable documented attempts to contact the participant have failed. Such attempts include, but are not limited to, the following:

a. Contact all of the participant's phone numbers and listed contacts (to be recorded in the source document when the participant enters the study), as applicable.
b. Send e-mail, SMS and or other form of electronic contact to the participant's addresses and contacts, as applicable.
c. Review available medical records/notes on details of hospitalizations, clinic visits, or other procedures that may indicate the participant's condition, as applicable.
d. Check local, regional, and national public records to locate the participant or seek his or her death certificate, as applicable and as permitted by law.

The information and dates of attempted contact should be documented in the participant's records and the participant's final status should be recorded in the appropriate eCRF. Once these steps have been exhausted and documented, the sponsor or its representative should be contacted for further guidance, if applicable.

9.6. EARLY STOPPING OR DISCONTINUING THE STUDY

The trial may be terminated by the sponsor, Principal Investigator, or at the request of local health and ethics authorities.

Among the causes for termination may be the detection of technical problems, the possibility that the security of the participant cannot be guaranteed, or even the possibility of natural disasters, civil wars, public calamities, widespread interruption of power and Internet supply, paralysis of postal services (strikes), or another cause that makes it impossible to conduct the survey.

Other possible reasons for interruption or suspension of the Principal Investigator or research center include, but are not limited to:

- Unsatisfactory participant inclusion rate or problems related to adherence to eligibility criteria;
- Serious violations or repeated deviations from the clinical protocol;
- Submission of inaccurate, incomplete, and/or out-of-period data;
- If any EAG or technical safety issues occur, i.e., the trial can be terminated when adverse event rates in the trial or other studies with the product indicate a possible danger to the participants' health caused by the drug;
- Persistent failure to adhere to the clinical protocol, Good Clinical Practice guidelines, regulations, or the clinical trial agreement;
- Suspension of the research center by the REC;
- Fraud or fraudulent conduct;
- Request from the Principal Investigator (e.g., inability to conduct the study).

In case of discontinuation of the study, for any reason, participants must be notified immediately by the Principal Investigator and will have continuity of care regardless of the study, and will be assured of the right to immediate free care until discharge from the hospital and scheduled return visits.

10. DATA MANAGEMENT

10.1. DATA MANAGEMENT

. Research-IGZ 1.0 software will be used for data management.

The main data management processes are listed below, and others are included in the data management plan (DMP) in detail in **APPENDIX 2.**

The DMP, as the data management guidance document, is written by the data manager (DM) and approved by the sponsor. Data management will be performed according to the time, content, and method defined by the DMP.

10.2. 1.0 RESEARCH-IGZ DATA MANAGEMENT 1.0

- **Electronic case report form (eCRF):** is designed is set up according to the logic check, and is released for use after passing the test and approval by the sponsor.
- **Data entry:** eCRF data comes from the source documents, which are obtained by entering the participants' data by the team responsible for data entry, according to the eCRF filling explanation. The data is collected directly on a tablet with cloud storage, with each user having access to their profile according to their permissions;
- **Source documents:** These are scanned and attached, when necessary, according to the phase of the study, to facilitate data verification;
- **Source data verification:** the monitor through its access permission can remotely monitor system data, access the attached source documents and validate the processes. Thus, it verifies the consistency of the data globally, and can intervene through questioning, on-site visits, or routine verification;
- **Questions and answers about data:** questions come from the Research-IGZ logic check question system, the monitor, the DM etc. the Principal Investigator, or someone delegated by him/her, must answer the questions in a timely manner. If necessary, the DM and monitor can question again until the data is "clean".
- **Signature of the Principal Investigator:** after entering the data and source data, the Principal Investigator must perform verification and confirmation by login and password. At the end, the digital signature will be required. If there are any data revisions after signing, they must be signed again.

- **Reporting:** Research-IGZ 1.0 can provide reports on adverse events and the progress of study participants, being identity-blind but non-blind to the system, and can generate partial data for efficacy evaluation, AE, schedule, and whatever is optimized from the database, without breaking the blinding.
- **Database Locking:** After the Principal Investigator, sponsor, statistical analyst and the DM jointly sign the registration, Research-IGZ 1.0 consolidates the database by allowing queries only.
- **Statistical analysis:** the statistician, through his profile and digital signature, can consult the database validated by the monitor, generating the spreadsheets that will be analyzed during and at the end of the study.
- **eCRF File:** Each participant's eCRF will generate PDF electronic file for preservation.
- **Research-IGZ Closure**: After completing the final report and project documents, a source/object file is generated allowing only queries on the Research-IGZ platform.

The external data transmission contract needs to be signed for DMP to manage the external data.

10.3. MEDICAL CODING

Participants' clinical findings should be reported using MedDRA (version 23.0), the drug combination adopts the WHO ATC for additional coding versus the Anatomic Therapeutic Chemical (ATC) classification.

10.4. DATA COLLECTION

All data collected must be recorded in the source documents and in the eCRF in an ALCOA-C form: Attributable, Legible, Contemporaneous, Original, Accurate and Complete. Data, such as reports of AEs, clinical examination results, and so on, should

be recorded when they occur, or as soon as it is possible to do so, in a detailed manner that reflects reality. Data should be traceable, so that any changes do not obscure the original data.

The study monitor, designated by the sponsor, will be responsible for reviewing the eCRF data against the source data for completeness and accuracy. If there is a discrepancy between the data, an action plan must be developed and implemented by the research center staff.

10.5. PARTICIPANT ID

The Principal Investigator agrees to complete a participant identification and inclusion record to allow easy identification of each participant during and after the study. Research participants will be identified in the eCRF, and in any other reports or communications, only by their identification number (registration number or PID, for randomized participants). The document will be reviewed by the sponsor's contact person for completeness. The record of the participant's ID number and inclusion will be treated as confidential and will be filed by the Principal Investigator in the study file. To ensure the confidentiality of the participant, no copies will be made.

10.6. SOURCE DOCUMENTS

The study source documents are all information, original records of clinical findings, observations, and laboratory and imaging test results necessary for the evaluation proposed in this study. The examinations and data recorded directly in the eCRF will be considered the raw data source of the study.

The research facility will maintain records of participants and imaging examinations appropriate for this study in accordance with ICH E6, Section 4.9 and in accordance with regulatory and institutional requirements for the protection of participant confidentiality.

10.7. DATA COLLECTION FORM

All data collected for the purposes of this study will be entered into an

electronic data collection form (Case Report Form - eCRF) developed specifically for the study. These will be accessed in a restricted manner by professionals delegated by the sponsor and Principal Investigator for the study, and stored in the cloud, with appropriate measures for data backup and system stability.

Data entry into the eCRF must be done by the Principal Investigator or a designated team member, and for this role, at all stages provided for in the study.

Data regarding each participant's examination results, where applicable, will be transcribed into the eCRF by the Principal Investigator and/or delegated team from the source documents as information becomes available.

The Sponsor will be responsible for the data management of this study, including checking the quality of the data. All entries, corrections and changes to the eCRF must be made by professionals who will ensure the confidentiality of the participant as well as the security and confidentiality of the data throughout the study.

After completion, all data will be securely transmitted to the sponsor within the timeframe agreed upon between the sponsor and the center. After all data has been collected and monitored, the eCRF will be considered closed and will be locked for changes.

10.8. PROTOCOL DEVIATIONS

The procedures of this study should be performed as described in the protocol, taking care that protocol deviations do not occur. A protocol deviation is any non-compliance with the principles of GCP and the study protocol. The Principal Investigator must assess each deviation that occurs during the conduct of the study for non-compliance with GCP regulations and national ethical and regulatory standards.

Deviation can occur either on the part of the participant, the Principal Investigator, or the research center staff**. Once deviation is identified, corrective actions must be developed by the site and implemented immediately.** Any deviation and appropriate corrective actions must be recorded, justified, and documented in the partial, if applicable, and final study reports, including supporting documentation.

Deviations can be problems with protocol adherence, including failure to obtain the

informed consent form, failure to meet eligibility criteria, incorrect performance of tests or analyses, failure to administer study medications correctly, and so on.

10.9. AMENDMENTS TO THE PROTOCOL

Amendments to the protocol must be approved and signed in the same manner as the protocol. The Principal Investigator should not implement any protocol amendments without first discussing the changes with the sponsor and obtaining documented approval from ethical and regulatory authorities, except when necessary to eliminate immediate risks to the participant or when the changes involve only logistical or administrative aspects of the trial (e.g., changing personnel, telephone numbers).

10.10. CONFIDENTIALITY AND DATA PROTECTION

It is the responsibility of the Principal Investigator or his/her delegate to collect, store, and disclose the study participants' information in an anonymous manner. All information must be traceable. **All trial documents, including letters of approval from the REC, source documents, SCRs and the like, must be archived by the Principal Investigator for a period of 5 years in accordance with national regulations.**

Only staff members assigned to the study should have access to the data collected, in addition to the sponsor's designees, so that the confidentiality and security of the research participants' data is ensured. The files will be named by including a participant ID. **Only the system and the physical security report will know the relationship between the participant's name and her corresponding number.** Unidentified data will be stored in the cloud and on secured computers.

All personal data collected relating to participants, Principal Investigator, or any person involved in the trial who may be included in the sponsor's databases must be handled in compliance with applicable laws and regulations, including the General Data Protection Regulation.

The data collected should be adequate, relevant, and not excessive in relation to the purposes for which it is collected. Each category of data should be properly

justified and in line with the purpose of the study.

10.11. MONITORING

A representative of the sponsor or his/her designee will visit the investigator periodically for the purpose of monitoring the progress of the trial in accordance with the protocol, GCP and local regulations. Non-adherence to the protocol, GCP and local regulations will be documented and corrective actions will be implemented if necessary. **It is the investigator's responsibility to be present or available for clarification during monitoring visits.**

The sponsor may use a combination of monitoring techniques in this trial: **central monitoring, remote monitoring and in-person monitoring at the research site.** The sponsor will conduct monitoring visits at the research site as frequently as necessary according to the clinical monitoring plan to ensure consistency between eCRFs and source documents. **During these routine visits, all data pertaining to a trial research participant will be made available to the trial monitor.** Discrepancies will be resolved in accordance with GCP principles. The monitor will record the dates of the visits in a research center visit log that will be maintained at the research center. The first post- initiation visit will be conducted as soon as possible after inclusion has begun.

10.12. AUDIT

Representatives of the sponsor may visit the research site at any time during or after completion of the trial to conduct an audit of the trial, in compliance with regulatory guidelines and company policy. Such audits will require access to all trial records, including source documents, for inspection. However, the privacy of the research participants must be respected. **The principal investigator and research site staff are responsible for being present and available for consultation during routinely scheduled research site audit visits conducted by the sponsor or its designees.**

Similar audit procedures may also be conducted by agents of any regulatory

agency, either as part of a national GCP compliance program or to review the results of this trial in support of a regulatory submission. The principal investigator should notify the sponsor immediately if contacted by a regulatory agency regarding an upcoming inspection.

10.13. QUALITY CONTROL AND ASSURANCE

In order to ensure the quality of the trial, the sponsor, ORPC and Principal Investigator should discuss and compose the clinical research plan together before the formal trial and conduct GCP training for the staff participating in the trial to ensure the safety of the participants and the efficacy of the clinical trial data, data monitoring and safety monitoring should be established.

The research institution should manage study drugs, including receipt, storage, distribution, and recycling in accordance with standard operating procedures.

According to the ICH GCP guidelines, the necessary steps should be discussed in the design and implementation phases of the trial to ensure the accuracy, consistency, integrity and credibility of the data collected. All observed results and abnormal findings in the clinical trial should be verified and recorded at the time of their occurrence to ensure data reliability. All types of instruments, equipment, reagents, standards, and so forth used during the clinical trial should be calibrated and have strict quality standards to ensure that they function under normal conditions.

The Principal Investigator, or someone delegated by him, should fill in the information required by the protocol in the original medical record, and the monitor should check whether the eCRF filing is complete and accurate or not, and then direct the research site staff to make corrections and additions if necessary. Drug administration authorities and the sponsor may entrust inspectors to conduct systematic inspection of activities and documents related to the clinical trial to assess whether the trial is conducted in accordance with the requirements of the trial protocol, SOPs, and relevant regulations or not, and whether the experimental data

are recorded timely, truthfully, accurately, and whether they are complete or not. The audit should be performed by personnel who are not directly involved in the clinical trial.

10.14. DATA ARCHIVING AND STORAGE

The Principal Investigator will keep all relevant study information for at least 5 (five) years after the end of the study (CNS/MS Resolution nº. 466/12). Access to this file will be controlled, being the responsibility of the Principal Investigator and the sponsor to properly maintain the conditions that allow the adequate storage of the study information.

Prior to the removal or destruction of the trial documents, the research facility must inform the sponsor in writing of its intentions. Only after obtaining written permission from the sponsor may the Principal Investigator remove or destroy the trial documents.

The study file may be checked by the Principal Investigator and research team, monitor, and regulatory agents to ensure that all necessary documents have been obtained prior to the start of the study and have been kept up-to-date during the study.

11. STATISTICAL ANALYSIS

11.1. SAMPLE SIZE ESTIMATION

This study is a randomized, parallel, double-blind, PLACEBO-controlled clinical trial, with the primary endpoint being the "Proportion of participants with improvement in clinical status [Period: until D15, approximately 14 days]".

This is a randomized, parallel, double-blind, PLACEBO-controlled clinical trial, which after initial study data, the sample size calculation was revised by updating parameters of frequency of clinical improvement and dropout rate. The revised sample size is 180 participants randomly and equally divided into the two arms of the study (90 practitioners in each group).

- Proportion of participants (%) with improvement of clinical status [Period: until D15, approximately 14 days].
- Clinical improvement time (median, in days) taking into account the following **PARAMETERS:**

○ Level of Significance	α = 5%
○ Statistical test power	Not less than 80%;
○ Distribution into groups	1:1 ratio
○ Frequency of clinical improvement	p1 = 66.7% in the test group;<br>p2 = 28.1% in the control group
○ Superior efficacy study in terms of frequency of clinical improvement	15% established in previous studies;
○ Median time to clinical improvement	5 days in the test group;<br>7 days in the control group.
○ Minimum detectable HR risk rate over time for clinical improvement	0,7

We use the results of the open-label, randomized, controlled trial report on the efficacy of AZVUDINE 1 mg tablets in the treatment of patients with COVID-19 (HOPE-1) [18], as reported below:

- The study included male/female patients 18 years and older with a mild course of COVID-19 who received treatment for COVID-19 ("treated") prior to enrollment in the study and, patients who did not receive treatment ("newly diagnosed"). All patients received AZVUDINE at a dose of 5 mg per day for 7 days;

- Analysis of this clinical trial shows that at the start of treatment, 100% of patients tested positive for SARS-CoV-2 RNA, 80% of patients had abnormalities on lung CT, and, 50% of patients had respiratory symptoms and signs.

- According to the WHO COVID-19 Ordinal Scale of Clinical Improvement, 50% of the patients were in category 1 and 50% were in category 2.

Following on in the report, analysis of individual patient data (regarding relief of symptoms and respiratory signs, body temperature, chest CT scan and testing for SARS-CoV-2 RNA), allowed us to estimate the individual time to clinical improvement, defined as a decrease in score on the WHO COVID-19 Ordinal Scale of Clinical Improvement in at least one category compared to screening, according to the table below:

[18] Randomized, open, controlled clinical trial of AZVUDINE tablets in the treatment of COVID-19. Study No. FNC-Hope1.Report. - P.-R. China, 2020

PACIENT NO.	GRUP	PATIENT STATUS IN TRIAGE	ORDINAL SCALE OF CLINICAL IMPROVEMENT OF THE WHO		TIME FROM START OF TREATMENT TO CLINICAL IMPROVEMENT, DAYS
			In screening	Day 7 of treatment (D7)	
001	Test Group	Treaty	1	0	3
002	Test Group	Treaty	2	0	6
003	Test Group	Treaty	1	0	4
004	Test Group	Treaty	1	0	5

005	Test Group	Treaty	2	0	1
020	Test Group	Treaty	1	0	5
010	Test Group	Newly diagnosed	1	0	4
011	Test Group	Newly diagnosed	1	0	5
016	Test Group	Newly diagnosed	1	0	5
019	Test Group	Newly diagnosed	1	0	6
006	Control Group	Treaty	2	0	7
007	Control Group	Treaty	2	0	4
008	Control Group	Treaty	2	2	8
009	Control Group	Treaty	2	0	5
012	Control Group	Treaty	1	1	8
013	Control Group	Treaty	2	1	6
014	Control Group	Newly diagnosed	2	2	14
015	Control Group	Newly diagnosed	2	1	7
017	Control Group	Newly diagnosed	2	1	4
018	Control Group	Newly diagnosed	1	1	14

Based on this report, "improvement in clinical status in at least one category compared to screening," as per the WHO Ordinal Scale of Clinical Improvement, occurred in 100% of the participants in the test group (10 out of 10) and, 60% in the

control group (6 out of 10).

Therefore the overall percentage of improvement, i.e. the proportion of participants who showed clinical improvement, occurred in 16 out of 20 subjects (16/20= 80%) participating in the HOPE-1 study.

- Calculated using the Wilson method, the **95% confidence intervals for the frequency of clinical improvement** for the clinical trial protocol **[66.7%;** 100%**]** in the test group and **[28.1%;** 85.2%**]** in the control group.

- Based on the **worst-case scenario,** the lower bounds of these confidence intervals are used as the expected ratios of clinical improvement to calculate the sample size: **p1 = 66.7% and p2 = 28.1%.**

The number of participants was calculated using R 4.0 software, as detailed below: Trial Size: Two Sample Proportion. NIS (alpha = 0.05, beta = 0.2, p1 = 0.667, p2 = 0.281, k = 1, delta = 0.386, margin = 0.205) = 80.043~ 81 por Grupo.

Number of participants	Sample Size	Drop-out (10%)
162 being:<br>81 Placebo group<br>81 Azvudine group	180<br>90 Placebo group<br>90 Azvudine group	18

"The required sample size for the IGZ-1 protocol is 180 participants, with 90 participants in each group, considering the 10% chance of drop-outs (participants who leave the study for some reason other than its termination)."

11.2. STATISTICAL ANALYSIS POPULATION

- Full Analysis Set (FAS): a set of patients who were randomized and received at least one oral dose of the study drug.
- Per protocol set (PPS): a set of data generated by a subset of clinical trial subjects whose protocol compliance was strict, with no significant

deviation from the trial protocol, to ensure that the data are statistically relevant to answer the primary endpoint. PPS analysis will be used for the primary efficacy indicator.

- Safety data set (SS): a set of participants who have received at least one treatment, and have actual data recorded by the safety index after treatment. The SS analysis will be used for the incidence of adverse reactions.

11.3. METHOD OF STATISTICAL ANALYSIS

All statistical tests will be two-sided tests.

- If the p-value is ≤0.05, it is considered that there is statistical significance in the test (unless otherwise specified).
- The quantitative indices describe the mean value, standard deviation, median, minimum value, maximum value, lower quartile (Q1), upper quartile (Q3) to be calculated, and the rank indices describe the frequency and percentage of cases.
- Comparison of the two groups under general conditions will be analyzed with appropriate methods according to the types of indicators. Student's t-test or Mann-Whitney test will be used to compare the groups for quantitative data. Chi-square test or Fisher's exact test will be used for categorical data.
- The selected cases should be grouped and at the end of the study determine the three analysis data sets (FAS, PPS, SS).

- For the analysis of demographic information and baseline eigenvalues, the mean value, standard deviation, quartiles, minimum value and maximum value should be calculated for numeric variables. For categorical data, frequency and percentage will be calculated.
- For analysis of medication and drug combination adherence one should calculate the percentage of individuals whose medication adherence is at

least 80-100%. An analysis of the classification of the combination of drugs should also be done.

- For analysis of the primary effectiveness indicator:

**Primary Goal**
To evaluate the **efficacy and safety of** AZVUDINE (FNC) versus placebo in patients infected with moderate to severe stage SARS-COV-2;
**Analysis method:**<br><br>For the efficacy analysis a logistic regression model will be used with the dependent variable "**The proportion of participants with clinical improvement [Period: until D15, approximately 14 days]"** and independent variables allocation group, AZVUDINE (FNC) or PLACEBO.<br>For the Safety analysis a logistic regression model will be used with the dependent variable **"occurrence of serious adverse events"** and independent variables allocation group, AZVUDINE (FNC) or PLACEBO.<br>As a support analysis, with exploratory character, both for efficacy and safety, covariate models will be built as well as other variables that present, in the univariate analysis, statistical significance.
**Secondary objective**<br>To evaluate the **clinical outcome** between the AZVUDINE (FNC) group versus placebo in patients infected with moderate to severe stage SARS-COV-2;
**Analysis method:**<br>Univariate analysis of the clinical outcome variables and with the group variable.

- For the secondary efficacy indicator analysis, the coronavirus viral load changes compared to baseline should be calculated for the experimental and control groups separately, as well as the value of the difference between the viral load changes compared to baseline for both groups. The ANCOVA model will be used to estimate the impact of treatment on viral load changes adjusting for other study variables such as disease severity, age, weight, and comorbidities, etc.

  - For analysis of the secondary efficacy indicator one should stratify according to the severity of the disease (severe, severe), following the following statistical methods:

PRIMARY ENDPOINT	ANALYSIS METHOD
• Proportion of participants with clinical status improvement [Period: until D15, approximately 14 days].	Teste t de Student/Teste de Mann-Whitney
○ The criterion for a participant to have an improvement in clinical status is a decrease on the WHO Clinical Improvement Ordinal Scale in at least one category compared to screening (WHO, Jun/2020).	

SECONDARY OUTCOMES	
• Proportion of participants with a clinical outcome of cure during the study; [Period: until D15, D21, and D28, approximately 14-30 days].	Pearson's Chi-square test

○ The clinical endpoint of cure is defined in this protocol as the absence of viral RNA in collected samples and clinical conditions for hospital discharge.	Student's t-test / Mann-Whitney test
• Time to normalization of body temperature (below 37.6c, axillary); [Period: until D15, D21 and D28, approximately 14-30 days].	Student's t-test / Mann-Whitney test
• Time to improvement of diarrhea, myalgia, fatigue, malaise, cough and headache; [Period: until D15, D21 and D28, approximately 14-30 days].	Student's t-test / Mann-Whitney test
• Changes in liver and kidney function *baselines*, and inflammatory and immunological markers: [Period: until D15, D21 and D28, approximately 14-30 days]	Student's t-test / Mann-Whitney test
○ Lab tests performed on days D1, D3, D5, D7, D9, D13, D15, D21, D28, and D60.	
• Evaluation of the time to negative conversion of SARS-CoV-2 viral load by RT-PCR between AZVUDINE (FNC) group and ~~placebo~~ control group; [Period: until D15, D21 and D28, approximately 14-30 days].	Student's t-test / Mann-Whitney test

• Evaluation of the number of cycles for the detection of SARS-CoV-2 viral load byRT-PCR and application of the standard curve for viral load calculation; [Period: until D15, D21 and D28, approximately 14-30 days].	Student's t-test / Mann-Whitney test
• Analysis of the relationship between the calculated viral load and the clinical outcome of participants ~~in the~~ AZVUDINE (FNC) group versus the ~~placebo~~ control group; [Period: until D15, D21 and D28, • approximately 14-30 days].	Generalized linearregression model
• Time and proportion of lung imaging improvement during treatment (Pattern of ground-glass opacities, mosaic paving, alveolar consolidation, reticular pattern/septal thickening, opacity with inverted halo, pleural/pericardial effusion, fibrosis and/or lymphadenomegaly); [Period: until D15, D21 and D28, approximately 14-30 days].	Student's t-test / Mann-Whitney test / Pearson's chi-square test
• Time of improvement and proportion of respiratory symptoms and signs during treatment (Lung rales, cough, sputum, sore throat); [Period: until D15, D21, and D28, approximately 14-30 days].	Student's t-test / Mann-Whitney test / Pearson's chi-square test

• Time to normalization of O_2 saturation (above 95%); [Period: until D15, D21 and D28, approximately 14-30 days].	Student's t-test / Mann-Whitney test
• Time to normalization of respiratory rate to levels ≤ 24rpm, in room air; [Period: until D15, D21 and D28, approximately 14-30 days]	Student's t-test / Mann-Whitney test
• Frequency of supplemental oxygenation or non-invasive ventilation; [Period: until D15, D21 and D28, approximately 14-30 days].	Student's t-test / Mann-Whitney test / Pearson's chi-square test
• Frequency of invasive mechanical ventilation or extracorporeal membrane oxygenation (ECMO); [Period: until D15, D21 and D28, approximately 14-30 days].	Student's t-test / Mann-Whitney test
• Proportion of moderate cases that progressed to severe cases requiring intensive care unit (ICU) care; [Period: until D15, D21, and D28, approximately 14-30 days].	Pearson's Chi-square test
• Length of hospital stay; [Period: until D15, D21 and D28, approximately 14-30 days].	Student's t-test / Mann-Whitney test

• Occurrence of drug interactions; [Period: until D15, D21 and D28, approximately 14-30 days].	Pearson's Chi-square test
• Frequency and intensity of adverse events; [Period: until D15, D21 and D28, approximately 14-30 days].	Student's t-test / Mann-Whitney test / Pearson's chi-square test
• Frequency and intensity of unexpected adverse events; [Period: until D15, D21 and D28, approximately 14-30 days].	Student's t-test / Mann-Whitney test / Pearson's chi-square test
• Frequency and intensity of serious adverse events; [Period: until D15, D21 and D28, approximately 14-30 days].	Student's t-test / Mann-Whitney test / Pearson's chi-square test
• All-cause mortality rate during the study; [Period: until D15, D21 and D28, approximately 14-30 days].	Student's t-test / Mann-Whitney test

• Evaluate the tolerability of AZVUDINE (FNC) use at 5mg/day regimen for up to 14 days: [Period: until D15, approximately 14 days].	Student's t-test / Mann-Whitney test / Pearson's chi-square test
○ Calculating treatment adherence;<br>○ Total time of use of AZVUDINE (FNC);<br>○ Proportion of participants who withdrew consent;<br>○ Proportion of participants who dropped out of treatment.	

For safety analysis, AEs should be coded according to the MedDRA terminology dictionary (version 23.0) and the following criteria should be listed and analyzed:

- Cases of EAs, EAGs, and of EAs leading to abandonment.
- Number and the rate of "normal to abnormal" or "abnormal worsening" of the laboratory and ECG indicator after the experiment.
- Laboratory indicator, ECG, abnormal physical examination cases and clinical interpretation.

11.4. MODEL AND OPERATING CHARACTERISTICS

Following initial study data, the sample size calculation was revised by updating parameters of frequency of clinical improvement and dropout rate. The required sample size for the IGZ-1 protocol is 180 participants, with 90 participants in each group, assuming a 10% dropout rate.

During the study, the Independent Data and Safety Monitoring Committee

(IMSC) will monitor adverse events, clinical, statistical, pharmacological and toxicological issues, and if necessary, experts in relevant areas can also be invited to attend the IMSC meeting.

At the end of the study, the participants' data will be analyzed and submitted to statistical analysis of the results according to the pre-established methodology.

12. ETHICAL AND REGULATORY CONSIDERATIONS AND GOOD CLINICAL PRACTICE

This protocol was prepared and will be conducted in accordance with the standards established by the ICH Guidelines for Good Clinical Practice GCP/ICH - ICH Topic E6 (R2) (2016), Americas Document (2005), Resolution No. 466/12 of the National Health Council and related, as well as the RDC No. 09 of February 2015 (ANVISA) and other applicable regulatory requirements. The data obtained will be used only for purposes determined in this research, keeping the confidentiality of data and results obtained ensured by controlled access of persons responsible for its evaluation and execution.

12.1. REGULATORY CONSIDERATIONS

Prior to initiation of the trial, the Principal Investigator must obtain written approval from the national regulatory authorities regarding the trial protocol and other documents that may be submitted. During the course of the trial, if there are further changes to the trial protocol, written approval from ANVISA must again be obtained.

12.2. INDEPENDENT DATA AND SECURITY MONITORING COMMITTEE (CIMS)

In order for the trial to meet scientific and ethical standards to the greatest extent possible, ensure the safety and well-being of the participants, as well as the scientific validity and integrity of the data, a CIMS has been established for the trial to review safety and clinical efficacy data, conduct emergency review and assessment of safety-related issues. CIMS members will evaluate adverse events, clinical, statistical, pharmacological and toxicological issues, and if necessary, experts in relevant fields can also be invited to attend the CIMS meeting.

The CIMS will be responsible for evaluating the safety and efficacy of the investigational drug. The CIMS may stop the study for safety.

Throughout the process of this trial, the CIMS will continuously monitor the efficacy and safety data, propose to the principal investigator and sponsor the organization of timely data discussion meeting, analyze the efficacy indicator and safety and will provide suggestions to the Principal Investigator and sponsor for the design of the follow-up trial according to the results of the statistical analysis. This committee will also conduct regular meetings or teleconferences according to the CIMS regulations, and the committee's recommendations include terminating the trial for safety, modifying the protocol, or suspending the trial until more supporting information is available, with the sponsor having priority in the final decision.

The CIMS regulation for working procedures can be found at ANNEX3.

12.3. ETHICAL CONSIDERATIONS AND RESEARCH ETHICS COMMITTEE (CEP)

According to the National Health Council Resolution No. 466/2012, the Operational Rule No. 001/2013 and complementary rules, research participants will be ensured full freedom to refuse to participate or to withdraw their consent at any stage of the research, without any penalty, and the maintenance of confidentiality and privacy during all phases of the research.

Prior to initiation of the study, the protocol, informed consent form, and other relevant documents will be submitted to the Research Ethics Committee for approval. The study may not begin until all relevant approvals have been obtained.

The research project will be registered in the Plataforma Brasil for ethical review by the Research Ethics Committee (CEP) of the Faculdade de Medicina de Campos, accredited by CONEP - CNS/MS. The research project will not be initiated until it has been approved by the CEP.

12.4. PROCESS FOR OBTAINING THE INFORMED CONSENT FORM (TCLE)

The Principal Investigator, or person delegated by him/her, will explain to each

participant the nature of the study, objectives, rationale, methods, and possible risks and benefits with their participation. Participants will be informed that they have the freedom to refuse to participate and may withdraw their consent, at any time and for any reason, without any prejudice. The Principal Investigator, or person delegated by him/her, will be available to answer questions and clarify any doubts. The research participant will have enough time to read the informed consent form, ask questions, and talk to friends and family if they feel they need to before deciding to participate in the study. The research participant will be informed that their privacy and the confidentiality of their data will be assured by the Principal Investigator and staff, and they will be assured that the data obtained from the research will be used only for the intended purpose. Any publication arising from this research will be carried out in a grouped form, not allowing the individual identification of the participants.

The Term of Consent (APPENDIX 4) will be presented in two copies of equal value, and must be initialed on all its pages and signed, at its conclusion, by the person invited to participate in the research, or by his/her legal representative, as well as by the Principal Investigator, or by the person(s) delegated by him/her, prior to his/her participation in this study.

If the participant or legally acceptable representative is unable to read or write, an impartial witness must be present for the entire informed consent process (which includes reading and explaining all written information) and must personally date and sign the ICF after the participant or legally acceptable representative gives verbal consent.

The Term of Consent must be revised whenever changes occur in the study procedures or when new information that may affect the potential participant's disposition becomes available. A consent form that is updated after its final approved version must be submitted for approval by the CEP. Participants must provide new consent to the most current version of the consent form.

12.5. REIMBURSEMENT

The research participants will be entitled to reimbursement for their expenses and those of their companions, when necessary, such as: food, transportation, laboratory tests, contraceptive methods, and other necessary costs during the conduction of the study, in compliance with the requirements established in items II.21 and IV.3.g of Resolution No. 466/2012 of the CNS.

12.6. PARTICIPANT ASSISTANCE

The sponsor and the Principal Investigator assure immediate, full and free assistance for complications and damages directly or indirectly related to the participation in the study for as long as necessary. Furthermore, all research participants who suffer any kind of damage because of their participation in this research are entitled to compensation, in compliance with the requirements established in items II.3, IV.7 and V.6 of Res. No. 466/2012.

12.7. CONFIDENTIALITY AND DISSEMINATION OF RESEARCH RESULTS

All staff involved in the trial should keep all information provided by the sponsor and ethical and regulatory authorities strictly confidential. Information should not be disclosed to third parties without written permission.

All data and experimental results of this trial are the joint property of the Principal Investigator and the sponsor. In compliance with the CNS Resolution

No. 466/2012 and Operational Standard CNS No. 001 of 2013 of the National Health Council, the Ministry of Health and other complementary to the CEP/CONEP System the results of the research will be published, whether favorable or not, with due credit to the Principal Investigator and technical staff involved and attached to Plataforma Brasil in the form of a Research Report.

The publication of data in specialized scientific journal or in events of the areas (congresses, symposiums, meetings) must be done in a grouped way to ensure the confidentiality of the data and the privacy of the participants meeting the regulatory requirements with the proper credits to the authors.

The results of the study will be disseminated among the research participants and the institutions where the data were obtained. The rights to all results and any

discovery or innovation arising from the study are reserved solely and exclusively to the sponsor.

This protocol and its results will be recorded on the www.clinicaltrials.gov platform.

12.8. RESPONSIBILITY OF THE PRINCIPAL INVESTIGATOR

The Principal Investigator is responsible for conducting the trial in compliance with the protocol, GCP, and ethical and regulatory requirements, and is responsible for personally supervising all staff involved in the clinical trial. The Principal Investigator can delegate tasks assigned to him/her, but not responsibility. The Principal Investigator must allow and cooperate with monitoring, audits and inspections.

The Principal Investigator must read and understand the IB, including possible risks and AEs of the trial medication, and ensure free appropriate medical care for participants as a result of any clinical trial-related AEs. The Principal Investigator must record and report to the sponsor any AEs that occur during the trial according to national regulations.

In the event of premature termination or suspension of the clinical trial for any reason, the Principal Investigator must immediately inform the trial participants, ensuring proper follow-up, safety, and well-being of the participants.

It is the responsibility of the Principal Investigator to ensure that protocol changes are made only after written approval from the sponsor and ethical and regulatory authorities, except to protect the safety, rights, or welfare of the participants.

It is the responsibility of the Principal Investigator, or the professional delegated by him/her, to register, store, and disclose the information from this study, as well as to ensure the quality and traceability of the data. **All study documents, including the conscientious opinion issued by the REC, source documents, ANVISA**

approval and others, shall be archived by the Principal Investigator for a period of 5 years, following the national regulations (Resolution no. 466/2012 - CNS/MS and RDC no. 9/2015 - ANVISA), or for a longer period, if agreed with the sponsor.

The principal investigator and the sponsor should sign on the protocol signature page to prove that they have both agreed to the contents of the protocol and that they will conduct and implement the trial according to the protocol.

12.9. RESPONSIBLE FOR THE AZVUDINE STUDY

The physician responsible for AZVUDINE in China is Professor Fujie Zhang, who is director and professor of medicine Clinical and Research Center of Infectious Diseases Beijing Ditan Hospital, Capital Medical University.

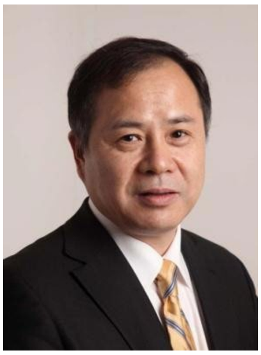

The signature of Dr.Zhang in Investigator's Brochure of AZVUDINE Tablets in China is as follows:

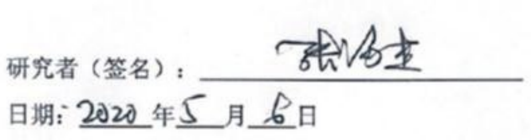
研究者（签名）：
日期：2020 年5 月6 日

The responsible Physician for AZVUDINE in Brazil

is:Paula Gebe Abreu Cabral, CRM: 52.52.956-01

Contact: (22) 98126 6602

The Principal Investigator of the IGZ-1 study is:

Cleber Gloria Silva, CRM: 52 76044-7

Contact (22) 99895 2290

12.10. DEADLINE FOR COMPLETION OF THE STUDY

The estimated time from screening (Day -1 or Day 1) to the end of the study (last follow-up visits D60) for a participant is approximately 60 days. As a total timeframe for the study, approximately 7 (seven) months is estimated, after study approval by the Research Ethics Committee (CEP) and ANVISA, for completion and delivery of the Final Report.

13. REFERENCES

Abdullah, S. F., & Sharquie, I. K. (2020). Sars-cov-2: A piece of bad news. *Medeniyet Medical Journal*, *35*(2), 151–160. https://doi.org/10.5222/MMJ.2020.82584

Caly, L., Druce, J. D., Catton, M. G., Jans, D. A., & Wagstaff, K. M. (2020). The FDA-approved drug ivermectin inhibits the replication of SARS-CoV-2 in vitro. *Antiviral Research*, *178*, 104787. https://doi.org/10.1016/j.antiviral.2020.104787

Carroll, S., & Olsen, D. (2006). Nucleoside Analog Inhibitors of Hepatitis C Virus Replication. *Infectious Disorders - Drug Targets*, *6*(1), 17–29. https://doi.org/10.2174/187152606776056698

Chibber, P., Assim, S., Ahmed, I., & Iqbal, N. (2020). *Advances in the possible treatment of COVID-19: A review*. *January*.

Choy, K.-T., Wong, A. Y.-L., Kaewpreedee, P., Sia, S. F., Chen, D., Hui, K. P. Y., Chu, D. K. W., Chan, M. C. W., Cheung, P. P.-H., Huang, X., Peiris, M., & Yen, H.-L. (2020). Remdesivir, lopinavir, emetine, and homoharringtonine inhibit SARS-CoV-2 replication in vitro. *Antiviral Research*, *178*, 104786. https://doi.org/10.1016/j.antiviral.2020.104786

Domingo, P., Mur, I., Pomar, V., Corominas, H., Casademont, J., & de Benito, N. (2020). The four horsemen of a viral Apocalypse: The pathogenesis of SARS-CoV-2 infection (COVID-19). *EBioMedicine*, *58*, 102887. https://doi.org/https://doi.org/10.1016/j.ebiom.2020.102887

Drożdżal, S., Rosik, J., Lechowicz, K., Machaj, F., Kotfis, K., Ghavami, S., & Łos, M. J. (2020). FDA approved drugs with pharmacotherapeutic potential for SARS-CoV-2 (COVID-19) therapy. *Drug Resistance Updates*, *53*, 100719. https://doi.org/10.1016/j.drup.2020.100719

Ezzikouri, S., Nourlil, J., Benjelloun, S., Kohara, M., & Tsukiyama-Kohara, K. (2020). Coronavirus disease 2019—Historical context, virology, pathogenesis, immunotherapy, and vaccine development. *Human Vaccines and Immunotherapeutics*, *00*(00), 1–9. https://doi.org/10.1080/21645515.2020.1787068

Fehr, A. R., & Perlman, S. (2015). Coronaviruses: An Overview of Their Replication and Pathogenesis. In *Coronaviruses: Methods and Protocols* (pp. 1–23). https://doi.org/10.1007/978-1-4939-2438-7_1

Götz, V., Magar, L., Dornfeld, D., Giese, S., Pohlmann, A., Höper, D., Kong, B.-W., Jans, D. A., Beer, M., Haller, O., & Schwemmle, M. (2016). Influenza A viruses escape from MxA restriction at the expense of efficient nuclear vRNP import. *Scientific Reports*, *6*(1), 23138. https://doi.org/10.1038/srep23138

Jordan, P. C., Stevens, S. K., & Deval, J. (2018). Nucleosides for the treatment of respiratory RNA virus infections. *Antiviral Chemistry and Chemotherapy*, *26*, 204020661876448. https://doi.org/10.1177/2040206618764483

Ministério da Saúde. (2020a). *Coronavírus: o que você precisa saber e como prevenir o contágio*. https://coronavirus.saude.gov.br/

Ministério da Saúde. (2020b). *Coronavírus Brasil*. https://covid.saude.gov.br/

Ministério da Saúde. (2020c). *Orientações para manejo de pacientes com Covid-19*. https://saude.gov.br/images/pdf/2020/June/17/Covid19-Orienta esManejoPacientes.pdf

MS/SCTIE. (2020). *DIRETRIZES PARA DIAGNÓSTICO E TRATAMENTO DA COVID-19*. Ministário Da Saúde - Secretaria de Ciência, Tecnologia, Inovação e Insumos Estratégicos Em SaúdeTecnologia, Inovação e Insumos Estratégicos Em Saúde. https://portalarquivos.saude.gov.br/images/pdf/2020/May/08/Diretriz-Covid19-v4-07-05.20h05m.pdf

Nitulescu, G. M., Paunescu, H., Moschos, S. A., Petrakis, D., Nitulescu, G., Ion, G. N. D., Spandidos, D. A., Nikolouzakis, T. K., Drakoulis, N., & Tsatsakis, A. (2020). Comprehensive analysis of drugs to treat SARS-CoV-2 infection: Mechanistic insights into current COVID-19 therapies (Review). *International Journal of Molecular Medicine*, *46*(2), 467–488. https://doi.org/10.3892/ijmm.2020.4608

Prefeitura de Campos. (2020). *Infoletim Nº 26/2020*. SMS/VIG EM SAÚDE/VIGILÂNCIA

EPIDEMIOLÓGICA. https://campos.rj.gov.br/newdocs/1608982625InformeEpidemologicoCampos26.pdf

Pruijssers, A. J., & Denison, M. R. (2019). Nucleoside analogues for the treatment of coronavirus infections. *Current Opinion in Virology*, *35*, 57–62. https://doi.org/10.1016/j.coviro.2019.04.002

Ren, Z. et. al. A Randomized, Open-Label, Controlled Clinical Trial of AZVUDINE Tablets in the Treatment of Mild and Common COVID-19, a Pilot Study. Adv. Sci. 2020, 7, 2001435. https://doi.org/10.1002/advs.202001435

Romano, M., Ruggiero, A., Squeglia, F., Maga, G., & Berisio, R. (2020). A Structural View of SARS-CoV-2 RNA Replication Machinery: RNA Synthesis, Proofreading and Final Capping. *Cells*, *9*(5), 1267. https://doi.org/10.3390/cells9051267

Sun, L., Peng, Y., Yu, W., Zhang, Y., Liang, L., Song, C., Hou, J., Qiao, Y., Wang, Q., Chen, J., Wu, M., Zhang, D., Li, E., Han, Z., Zhao, Q., Jin, X., Zhang, B., Huang, Z., Chai, J., … Chang, J. (2020). Mechanistic Insight into Antiretroviral Potency of 2′-Deoxy-2′-β-fluoro-4′-azidocytidine (FNC) with a Long-Lasting Effect on HIV-1 Prevention. *Journal of Medicinal Chemistry*, *63*(15), 8554–8566. https://doi.org/10.1021/acs.jmedchem.0c00940

Tay, M. Y. F., Fraser, J. E., Chan, W. K. K., Moreland, N. J., Rathore, A. P., Wang, C., Vasudevan, S. G., & Jans, D. A. (2013). Nuclear localization of dengue virus (DENV) 1–4 non-structural protein 5; protection against all 4 DENV serotypes by the inhibitor Ivermectin. *Antiviral Research*, *99*(3), 301–306. https://doi.org/10.1016/j.antiviral.2013.06.002

Wagstaff, K. M., Sivakumaran, H., Heaton, S. M., Harrich, D., & Jans, D. A. (2012). Ivermectin is a specific inhibitor of importin α/β-mediated nuclear import able to inhibit replication of HIV-1 and dengue virus. *Biochemical Journal*, *443*(3), 851–856. https://doi.org/10.1042/BJ20120150

Wang, M., Cao, R., Zhang, L., Yang, X., Liu, J., Xu, M., Shi, Z., Hu, Z., Zhong, W., & Xiao, G. (2020). Remdesivir and chloroquine effectively inhibit the recently emerged novel coronavirus (2019-nCoV) in vitro. *Cell Research*, *30*(3), 269–271. https://doi.org/10.1038/s41422-020-0282-0

Wang, R.-R., Yang, Q.-H., Luo, R.-H., Peng, Y.-M., Dai, S.-X., Zhang, X.-J., Chen, H., Cui, X.-Q., Liu, Y.-J., Huang, J.-F., Chang, J.-B., & Zheng, Y.-T. (2014). AZVUDINE, A Novel Nucleoside Reverse Transcriptase Inhibitor Showed Good Drug Combination Features and Better Inhibition on Drug-Resistant Strains than Lamivudine In Vitro. *PLoS ONE*, *9*(8), e105617. https://doi.org/10.1371/journal.pone.0105617

WHO. (2020a). *Clinical management of COVID-19*. World Health Organization. https://www.who.int/publications/i/item/clinical-management-of-covid-19

WHO. (2020b). *Coronavirus disease (COVID-19) - Situation Report – 204*. World Health Organization. https://www.who.int/docs/default-source/coronaviruse/situation-reports/20200811-covid-19-sitrep-204.pdf?sfvrsn=1f4383dd_2

WHO. (2020c). *COVID 19 Experimental Treatments*. https://www.who.int/docs/default-source/coronaviruse/covid-classification-of-treatment-types-rev.pdf?sfvrsn=5b90b2f2_1&download=true

WHO. (2020d). *Q&A on coronaviruses (COVID-19)*. https://www.who.int/emergencies/diseases/novel-coronavirus-2019/question-and-answers-hub/q-a-detail/q-a-coronaviruses

WHO. (2020e). *WHO Coronavirus Disease (COVID-19) Dashboard | WHO Coronavirus Disease (COVID-19) Dashboard*. World Health Organization. https://covid19.who.int/

Zhand, S., Saghaeian Jazi, M., Mohammadi, S., Tarighati Rasekhi, R., Rostamian, G., Kalani, M. R., Rostamian, A., George, J., & Douglas, M. W. (2020). COVID-19: The Immune Responses and Clinical Therapy Candidates. *International Journal of Molecular Sciences*, *21*(15). https://doi.org/10.3390/ijms21155559

13.1. ANNEX 1 –Treatment Protocol for COVID-19 - 2nd Version (July 02, 2020)

13.2. ANNEX 2 –Data Management Plan

13.3. ANNEX 3 – CIMS Internal Regulation - V2.0_19.01.2021

13.4. ANNEX 4 – Informed Consent Form (ICF)

13.5. ANNEX 5 - Hope-4 Partial Data Report on the Efficacy and Safety of AZVUDINE
