# Supplementary material for: Phase III, randomized, double-blind, placebo-controlled clinical study: a study on the safety and clinical efficacy of AZVUDINE in moderate COVID-19 patients
Source: Front Med (Lausanne). 2023 Oct 19;10:1215916. doi: 10.3389/fmed.2023.1215916 (PMC10620601; doi:10.3389/fmed.2023.1215916)
Supplement: Supplementary file 2 [file Data_Sheet_2.docx]

**SUPPLEMENTARY INFORMATION**

1. **SUPLEMENTARY METHODS**

**Identification of the dose to be used in the study**

Pre-clinical trials were carried out for much longer than suggested, leading to a total human dose equivalent.

Table: Dose ratio and time of use

| Animal/human equivalent dose | Animal / Time of drug use | Total dose used in preclinical tests over time |
| --- | --- | --- |
| 3.72 mg | **1 month** | 116.6 mg |
| 5.6 mg | **3 months** | 504.0 mg |
| 3.36 mg | **6,5 meses (26 weeks)** | 655,20 mg |
| 3.72 mg | **9,75 meses (39 weeks)** | 1.088,10 mg |

In preclinical trials, exposure to the drug for a prolonged period of time has shown that of the observable adverse events appear when the dose is exceeded by 20-30 times, but as the tests were carried out for a much longer time than suggested, AZVUDINE proved to be safe at the dose used.

For the IGZ-1 study, the proposed treatment period is 14 days (in view of the average incubation time of the coronavirus) and the maximum dose of Azvudine used in the 14 days is 70mg, remembering that the dose may be lower, because the participant may be discharged earlier.

The results of the pre-clinical study of FNC, in dogs, show that NOAEL=0.1mg/kg. Using the calculation formula 60×{[NOAEL]/(human kg/animal kg)}/10}) to calculate the equivalent dosage corresponding to the human body based on the NOAEL dosage of the most sensitive species and then dividing by safety coefficient ( 10) to obtain the initial dosage for the human body, the initial dosage for the human body was 0.36mg.

According to long-term toxicity data from dogs, taking 1/3 dose and maximum tolerance 0.3mg/kg, that is, 0.1mg/kg, to calculate human dosage (weight was calculated as 60kg), the maximum test dose would be 6mg/human/day.

As for the safety profile related to the dose used in humans, the maximum study of the safety dose of AZVUDINE was carried out for 5 mg, and a daily dose of 5 mg has an average life of 13.8 h, being excreted in the urine in up to 24 hours.

The proposed therapeutic regimen for the phase III clinical trial in patients with COVID-19 is based on preliminary studies conducted in China in 41 patients with pneumonia caused by SARS-CoV-2. The dose used was 5 mg/day, and the observed AEs were mild to moderate, with no need for medical intervention. All patients were discharged after two consecutive RT-PCR tests.

Dose explorer phase I clinical trial (GQ-FNC-2014) evaluated the safety of Azvudine in HIV-positive patients for 7 days and demonstrated that Azvudine has clinical safety. **Azvudine regiment dose groups for 2mg, 3mg, 4mg and 5mg showed good safety.** No participants in the Azvudine 3mg and 5mg groups reported any AEs and no serious adverse events were attributed to Azvudine. 8 patients with HIV received a dose of Azvudine 5mg, and no adverse drug reactions were observed. In these patients the plasma concentration of Azvudine was similar to the concentration observed in patients who received a dose of 2 to 4 mg. **Furthermore, the 5 mg dose led to a human plasma concentration (2.42 ng/mL)** which is 3,099 times lower than the concentration at the dose used in the CHL cell chromosome structure aberration test at a concentration of 7.5μg/mL after 4 hour exposure with or without S9 metabolic activation system.

Based on these studies, there was evidence that the 5 mg dose would have a good safety profile commensurate with use in the proposed target population.

1. **SUPPLEMENTARY RESULTS**


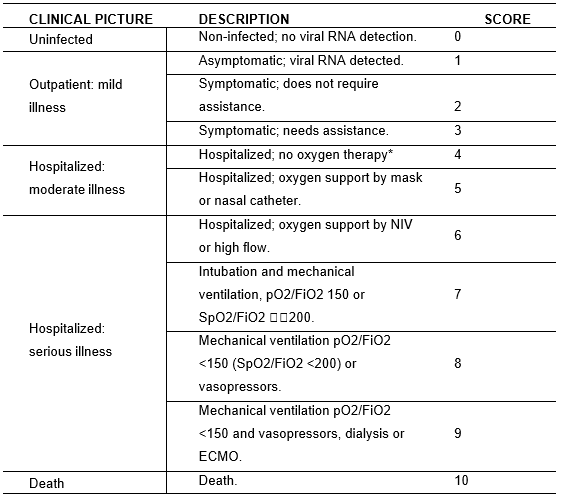


**Supplementary Figure 1:** scores of the study related to clinical picture. *Table 4: WHO Clinical Progression Scale (Jun 2020)*

**Supplementary Figure 2:** Proportion of the initial and final clinical score of all subjects in the FNC group and the placebo group. The p value represents the significant difference between groups by the Wilcoxon test (Left bar: FNC; Right bar: PLACEBO).

**Supplementary Table 1**: **Time for improvement of symptoms.**

|  | | TREATMENTS | |  |
| --- | --- | --- | --- | --- |
| Overall | N | FNC, N = 91^1^ | Placebo, N = 88^1^ | p-value^2^ |
| COUGH (Qty. days) | 179 | 3.2 ± 3.6 (2.0) | 3.5 ± 3.4 (3.0) | 0.305 |
| MYALGIA (Qty. days) | 179 | 1.59 ± 3.00 (0.00) | 1.83 ± 2.79 (0.00) | 0.259 |
| SMELL LOSS (Qty. days) | 179 | 1.6 ± 3.4 (0.0) | 1.8 ± 3.1 (0.0) | 0.347 |
| TASTE LOSS (Qty. days) | 179 | 1.6 ± 3.3 (0.0) | 1.7 ± 3.1 (0.0) | 0.479 |
| DIARRHEA (Qty. days) | 179 | 0.16 ± 0.70 (0.00) | 0.13 ± 0.50 (0.00) | 0.981 |
| DIZZINESS (Qty. days) | 179 | 0.19 ± 1.09 (0.00) | 0.20 ± 0.86 (0.00) | 0.531 |
| FEVER (Qty. days) | 179 | 0.13 ± 0.50 (0.00) | 0.38 ± 0.68 (0.00) | **<0.001** |
| CHILL (Qty. days) | 179 | 0.21 ± 0.64 (0.00) | 0.76 ± 1.95 (0.00) | **0.008** |
| SORE THROAT (Qty. days) | 179 | 0.74 ± 1.65 (0.00) | 1.13 ± 2.34 (0.00) | 0.195 |
| CORYZA (Qty. days) | 179 | 0.35 ± 0.91 (0.00) | 0.48 ± 1.09 (0.00) | 0.392 |
| DYSPNEA (Qty. days) | 179 | 0.86 ± 1.80 (0.00) | 0.95 ± 2.07 (0.00) | 0.671 |
| TACHYPNEA (Qty. days) | 179 | 0.32 ± 1.31 (0.00) | 0.31 ± 0.78 (0.00) | 0.262 |
| NAUSEA (Qty. days) | 179 | 0.03 ± 0.18 (0.00) | 0.10 ± 0.40 (0.00) | 0.174 |
| VOMIT (Qty. days) | 179 | 0.0440 ± 0.2061 (0.0000) | 0.0568 ± 0.3165 (0.0000) | 0.764 |
| ABDOMINAL PAIN (Qty. days) | 179 | 0.1319 ± 0.6533 (0.0000) | 0.0455 ± 0.2586 (0.0000) | 0.488 |
| INABILITY TO MARCH (Qty. days) | 179 | 0.0549 ± 0.2734 (0.0000) | 0.1023 ± 0.6072 (0.0000) | 0.770 |
| ^1^Mean ± SD (Median) | | | | |
| ^2^Wilcoxon rank sum test | | | | |

**Supplementary Table 2: Time to improve lung image enhancement.**

|  | TREATMENTS | | | | | |  | | | |  |
| --- | --- | --- | --- | --- | --- | --- | --- | --- | --- | --- | --- |
| Overall | | N | FNC, N = 91^1^ | | | Placebo, N = 88^1^ | | p-value^2^ | | |  |
| CT Scan (D1) | 152 | | | 30 ± 24 (30) | 34 ± 25 (30) | | | | 0.530 |  |  |
| CT Scan (D3) | 161 | | | 31 ± 24 (30) | 34 ± 24 (30) | | | | 0.483 |  |  |
| CT Scan (D7) | 114 | | | 33 ± 24 (30) | 35 ± 24 (32) | | | | 0.602 |  |  |
| CT Scan (D11) | 64 | | | 30 ± 24 (25) | 35 ± 22 (30) | | | | 0.297 |  |  |
| CT Scan (D15) | 163 | | | 22 ± 22 (20) | 20 ± 23 (5) | | | | 0.512 |  |  |
| CT Scan (D28) | 133 | | | 6 ± 11 (1) | 6 ± 11 (1) | | | | 0.935 |  |  |
| ^1^Mean ± SD (Median); n (%) | | | | | | | | | | |  |
| ^2^Wilcoxon rank sum test; Pearson's Chi-squared test; Fisher's exact test; Wilcoxon rank sum exact test | | | | | | | | | | | |


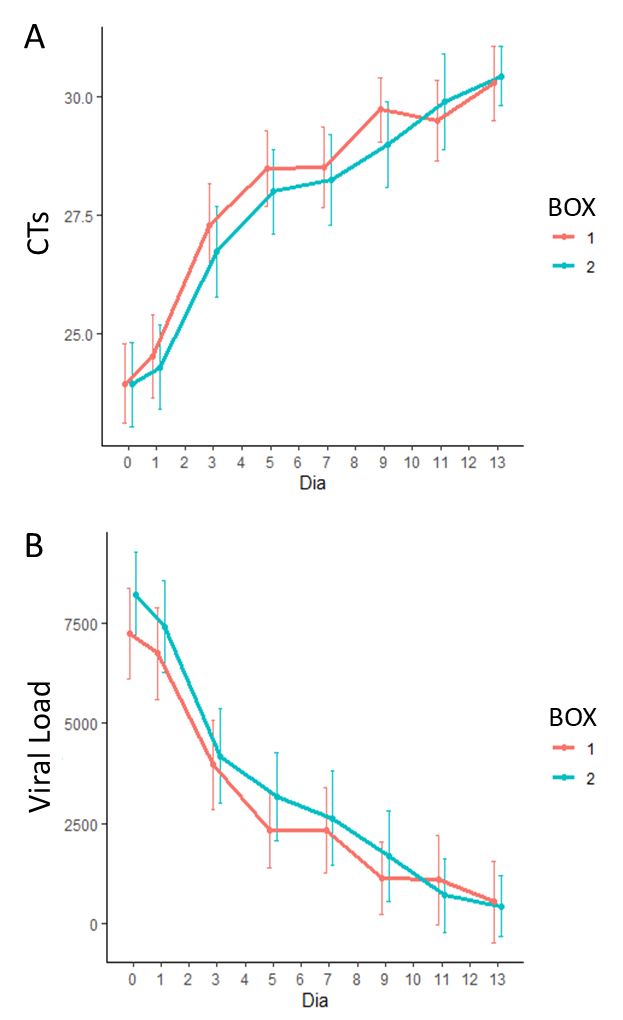

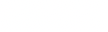

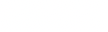


RT-PCR

**Supplementary Figure 3:** CTs (A) and viral load (B) analysis measured by RT-PCR of all participants in the FNC group and the placebo group. Data are median (SD). (Red line: FNC; Blue line: PLACEBO).

**Supplementary Table 3: Comorbidities**

|  | | | TREATMENTS | | | |  |
| --- | --- | --- | --- | --- | --- | --- | --- |
| Overall | N | Total | | FNC, N = 91^1^ | Placebo, N = 88^1^ | p-value^2^ | |
| Arterial hypertension | 180 | 76 (42%) | | 37 (41%) | 39 (44%) | 0.799 | |
| Obesity | 180 | 45 (25%) | | 21 (23%) | 24 (27%) | 0.704 | |
| Hepatic steatosis | 180 | 0 (0%) | | 0 (0%) | 0 (0%) |  | |
| Type 1 diabetes | 180 | 4 (2.2%) | | 3 (3.3%) | 1 (1.1%) | 0.629 | |
| Type 2 diabetes | 180 | 30 (17%) | | 12 (13%) | 18 (20%) | 0.360 | |
| Smoker | 180 | 3 (1.7%) | | 1 (1.1%) | 2 (2.3%) | 0.623 | |
| Alcoholic | 180 | 31 (17%) | | 11 (12%) | 19 (22%) | **0.033** | |
| Asthma | 180 | 5 (2.8%) | | 3 (3.3%) | 2 (2.3%) | >0.999 | |
| Bronchitis | 180 | 10 (5.6%) | | 3 (3.3%) | 6 (6.8%) | **0.026** | |
| Cardiac arrhythmia | 180 | 7 (3.9%) | | 2 (2.2%) | 5 (5.7%) | 0.301 | |
| Angina | 180 | 1 (0.6%) | | 0 (0%) | 1 (1.1%) | 0.494 | |
| Kidney stone | 180 | 0 (0%) | | 0 (0%) | 0 (0%) |  | |
| Gallstone | 180 | 1 (0.6%) | | 1 (1.1%) | 0 (0%) | >0.999 | |
| Bariatric | 180 | 0 (0%) | | 0 (0%) | 0 (0%) |  | |
| Stroke | 180 | 0 (0%) | | 0 (0%) | 0 (0%) |  | |
| Vaccine | 180 | 0 (0%) | | 0 (0%) | 0 (0%) |  | |
| Cardiac revascularization | 180 | 0 (0%) | | 0 (0%) | 0 (0%) |  | |
| Stent | 180 | 1 (0.6%) | | 1 (1.1%) | 0 (0%) | >0.999 | |
| Osteoporosis | 180 | 2 (1.1%) | | 1 (1.1%) | 1 (1.1%) | >0.999 | |
| ^1^n (%) | | | | | | | |
| ^2^Teste exato de Fisher | | | | | | | |

**Supplementary Table 4: Demonstration of values for other variables during the days of study.**

|  | | TREATMENTS | |  |
| --- | --- | --- | --- | --- |
| Overall | N | FNC, N = 911 | Placebo, N = 88^1^ | p-value^2^ |
| COUGH (Qty. days) | 179 | 3.2 ± 3.6 (2.0) | 3.5 ± 3.4 (3.0) | 0.305 |
| MYALGIA (Qty. days) | 179 | 1.59 ± 3.00 (0.00) | 1.83 ± 2.79 (0.00) | 0.259 |
| SMELL LOSS (Qty. days) | 179 | 1.6 ± 3.4 (0.0) | 1.8 ± 3.1 (0.0) | 0.347 |
| TASTE LOSS (Qty. days) | 179 | 1.6 ± 3.3 (0.0) | 1.7 ± 3.1 (0.0) | 0.479 |
| DIARRHEA (Qty. days) | 179 | 0.16 ± 0.70 (0.00) | 0.13 ± 0.50 (0.00) | 0.981 |
| DIZZINESS (Qty. days) | 179 | 0.19 ± 1.09 (0.00) | 0.20 ± 0.86 (0.00) | 0.531 |
| FEVER (Qty. days) | 179 | 0.13 ± 0.50 (0.00) | 0.38 ± 0.68 (0.00) | **<0.001** |
| CHILL (Qty. days) | 179 | 0.21 ± 0.64 (0.00) | 0.76 ± 1.95 (0.00) | **0.008** |
| SORE THROAT (Qty. days) | 179 | 0.74 ± 1.65 (0.00) | 1.13 ± 2.34 (0.00) | 0.195 |
| CORYZA (Qty. days) | 179 | 0.35 ± 0.91 (0.00) | 0.48 ± 1.09 (0.00) | 0.392 |
| DYSPNEA (Qty. days) | 179 | 0.86 ± 1.80 (0.00) | 0.95 ± 2.07 (0.00) | 0.671 |
| TACHYPNEA (Qty. days) | 179 | 0.32 ± 1.31 (0.00) | 0.31 ± 0.78 (0.00) | 0.262 |
| NAUSEA (Qty. days) | 179 | 0.03 ± 0.18 (0.00) | 0.10 ± 0.40 (0.00) | 0.174 |
| VOMIT (Qty. days) | 179 | 0.0440 ± 0.2061 (0.0000) | 0.0568 ± 0.3165 (0.0000) | 0.764 |
| ABDOMINAL PAIN (Qty. days) | 179 | 0.1319 ± 0.6533 (0.0000) | 0.0455 ± 0.2586 (0.0000) | 0.488 |
| INABILITY TO MARCH (Qty. days) | 179 | 0.0549 ± 0.2734 (0.0000) | 0.1023 ± 0.6072 (0.0000) | 0.770 |
| ^1^Mean ± SD (Median) | | | | |
| ^2^Wilcoxon rank sum test | | | | |

**Supplementary Table 5: Analysis of urinary phosphorus excretion in 24 hours.**

|  | TREATMENTS | | | |  | | |
| --- | --- | --- | --- | --- | --- | --- | --- |
| Overall | | N | FNC, N = 91^1^ | Placebo, N = 88^1^ | | p-value^2^ | |
| URINE PHOSPHORUS 24h (D1) | | 149 | 423 ± 586 (28) | 360 ± 534 (0) | | 0.361 | |
| URINE PHOSPHORUS 24h (D15) | | 55 | 43.79 ± 230.15 (3.20) | 3.43 ± 0.51 (3.50) | | 0.263 | |
| ^1^Mean ± SD (Median); n (%) | | | | | | |  |
| ^2^Wilcoxon rank sum test; Wilcoxon rank sum exact test | | | | | | | |

**Supplementary Table 6: Demonstration of serum cholinesterase values during the study days.**

|  | TREATMENTS | | | |  | | |
| --- | --- | --- | --- | --- | --- | --- | --- |
| Overall | | N | FNC, N = 91^1^ | Placebo, N = 88^1^ | | p-value^2^ | |
| Cholinesterase (D1) | | 100 | 6,059 ± 6,672 (0) | 3,816 ± 6,007 (0) | | 0.071 | |
| Cholinesterase (D15) | | 166 | 14,145 ± 15,225 (12,748) | 12,502 ± 2,459 (12,744) | | 0.710 | |
| Cholinesterase (D28) | | 55 | 10,672 ± 6,156 (13,827) | 9,496 ± 6,492 (12,045) | | 0.469 | |
| ^1^Mean ± SD (Median); n (%) | | | | | | |  |
| ^2^Wilcoxon rank sum test; Wilcoxon rank sum exact test | | | | | | | |

**Supplementary Table 7: Demonstration of inflammatory marker values during the study days.**

|  | TREATMENTS | | | |  | | |
| --- | --- | --- | --- | --- | --- | --- | --- |
| Overall | | N | FNC, N = 91^1^ | Placebo, N = 88^1^ | | p-value^2^ | |
| INTERLEUKIN-6 (D1) | | 168 | 18 ± 31 (8) | 25 ± 35 (10) | | 0.168 | |
| INTERLEUKIN-6 (D7) | | 106 | 18 ± 42 (4) | 9 ± 12 (6) | | 0.709 | |
| INTERLEUKIN-6 (D15) | | 146 | 9 ± 13 (4) | 8 ± 10 (4) | | 0.718 | |
| INTERLEUKIN-6 (D28) | | 128 | 3.97 ± 4.68 (2.60) | 3.37 ± 2.40 (2.60) | | 0.747 | |
| CPK-MB (D1) | | 178 | 1.25 ± 1.68 (0.64) | 0.99 ± 1.01 (0.70) | | 0.814 | |
| CPK-MB (D7) | | 109 | 0.60 ± 0.48 (0.45) | 0.69 ± 0.81 (0.48) | | 0.891 | |
| CPK-MB (D15) | | 165 | 0.73 ± 0.70 (0.48) | 0.60 ± 0.65 (0.29) | | 0.097 | |
| CPK-MB (D28) | | 108 | 1.64 ± 4.49 (0.66) | 1.11 ± 2.56 (0.56) | | 0.978 | |
| TROPONIN I (D1) | | 179 | 0.18 ± 0.22 (0.10) | 0.14 ± 0.10 (0.10) | | 0.192 | |
| TROPONIN I (D7) | | 107 | 0.17 ± 0.18 (0.10) | 0.18 ± 0.28 (0.10) | | 0.594 | |
| TROPONIN I (D15) | | 155 | 0.18 ± 0.18 (0.10) | 0.13 ± 0.09 (0.10) | | 0.086 | |
| TROPONIN I (D28) | | 106 | 0.47 ± 1.21 (0.10) | 0.20 ± 0.32 (0.10) | | 0.413 | |
| Serum lactate (D1) | | 172 | 14.5 ± 5.5 (13.9) | 13.9 ± 5.1 (13.3) | | 0.471 | |
| Serum lactate (D3) | | 174 | 18 ± 7 (17) | 18 ± 8 (16) | | 0.824 | |
| Serum lactate (D5) | | 140 | 21 ± 9 (21) | 19 ± 7 (18) | | 0.182 | |
| Serum lactate (D7) | | 109 | 19 ± 8 (18) | 17 ± 6 (15) | | 0.300 | |
| Serum lactate (D9) | | 79 | 17 ± 8 (16) | 16 ± 6 (15) | | 0.976 | |
| Serum lactate (D11) | | 63 | 13.7 ± 5.6 (12.5) | 14.4 ± 4.6 (15.5) | | 0.231 | |
| Serum lactate (D13) | | 36 | 16 ± 7 (14) | 15 ± 8 (13) | | 0.669 | |
| Serum lactate (D15) | | 164 | 17 ± 6 (16) | 17 ± 10 (15) | | 0.692 | |
| Activated partial thromboplastin time (D1) | | 179 | 34 ± 8 (32) | 33 ± 6 (32) | | 0.795 | |
| Activated partial thromboplastin time (D15) | | 170 | 62 ± 310 (29) | 28 ± 10 (30) | | 0.371 | |
| Activated partial thromboplastin time (D28) | | 130 | 32.7 ± 9.7 (31.6) | 38.8 ± 35.8 (33.8) | | **0.023** | |
| Prothrombin time (D1) | | 179 | 11.92 ± 2.48 (11.50) | 11.60 ± 1.71 (11.60) | | 0.603 | |
| Prothrombin time (D15) | | 170 | 10.4 ± 3.9 (11.5) | 10.5 ± 3.5 (11.2) | | 0.499 | |
| Prothrombin time (D28) | | 130 | 14.49 ± 17.67 (11.30) | 11.70 ± 1.33 (11.50) | | 0.540 | |
| Thrombin time (D1) | | 179 | 11.8 ± 8.2 (16.0) | 12.6 ± 8.0 (17.0) | | 0.327 | |
| Thrombin time (D15) | | 170 | 14 ± 9 (18) | 13 ± 8 (17) | | 0.293 | |
| Thrombin time (D28) | | 130 | 0.0000 ± 0.0000 (0.0000) | 0.0000 ± 0.0000 (0.0000) | |  | |
| Fibrinogen (D1) | | 179 | 473 ± 268 (522) | 456 ± 260 (499) | | 0.509 | |
| Fibrinogen (D15) | | 170 | 175 ± 256 (0) | 200 ± 258 (0) | | 0.433 | |
| Fibrinogen (D28) | | 130 | 0.0000 ± 0.0000 (0.0000) | 0.0000 ± 0.0000 (0.0000) | |  | |
| ESR (D1) | | 174 | 60 ± 27 (58) | 53 ± 27 (46) | | **0.047** | |
| ESR (D7) | | 109 | 46 ± 28 (38) | 45 ± 25 (42) | | 0.964 | |
| ESR (D15) | | 158 | 48 ± 25 (42) | 52 ± 21 (52) | | 0.235 | |
| ESR (D28) | | 106 | 19 ± 15 (20) | 19 ± 11 (18) | | 0.753 | |
| PLATELETS (D1) | | 179 | 288,857 ± 96,231 (279,000) | 267,760 ± 87,731 (265,500) | | 0.154 | |
| PLATELETS (D3) | | 178 | 342,712 ± 111,911 (330,000) | 323,898 ± 110,767 (309,000) | | 0.169 | |
| PLATELETS (D5) | | 165 | 395,475 ± 111,615 (390,000) | 375,840 ± 122,612 (359,000) | | 0.173 | |
| PLATELETS (D7) | | 133 | 404,002 ± 116,375 (382,500) | 375,254 ± 115,688 (364,000) | | 0.148 | |
| PLATELETS (D9) | | 98 | 370,806 ± 128,207 (332,000) | 359,204 ± 114,579 (362,000) | | 0.856 | |
| PLATELETS (D11) | | 79 | 342,444 ± 120,177 (334,500) | 318,293 ± 126,402 (309,000) | | 0.400 | |
| PLATELETS (D13) | | 51 | 339,571 ± 133,038 (296,000) | 280,413 ± 88,940 (287,000) | | 0.190 | |
| PLATELETS (D15) | | 169 | 364,739 ± 109,795 (357,500) | 328,223 ± 108,760 (310,000) | | **0.023** | |
| PLATELETS (D28 | | 130 | 280,422 ± 85,312 (284,000) | 272,695 ± 80,559 (260,150) | | 0.434 | |
| ^1^Mean ± SD (Median); n (%) | | | | | | |  |
| ^2^Wilcoxon rank sum test; Wilcoxon rank sum exact test | | | | | | | |

**Supplementary Table 8: Demonstration of white series values and immunological markers during the study days**

|  | TREATMENTS | | | |  | |
| --- | --- | --- | --- | --- | --- | --- |
| Overall | | N | FNC, N = 91^1^ | Placebo, N = 88^1^ | | p-value^2^ |
| LEUKOCYTES (D1) | | 179 | 9,015 ± 8,651 (7,700) | 9,381 ± 3,748 (8,500) | | **0.045** |
| LEUKOCYTES (D3) | | 178 | 10,459 ± 3,805 (10,220) | 11,109 ± 4,602 (10,600) | | 0.448 |
| LEUKOCYTES (D5) | | 165 | 11,833 ± 3,973 (11,400) | 12,135 ± 3,584 (12,100) | | 0.425 |
| LEUKOCYTES (D7) | | 133 | 10,172 ± 3,131 (10,000) | 11,370 ± 3,626 (10,600) | | 0.087 |
| LEUKOCYTES (D9) | | 98 | 9,096 ± 2,866 (9,400) | 9,804 ± 3,453 (8,700) | | 0.614 |
| LEUKOCYTES (D11) | | 79 | 8,181 ± 2,849 (8,000) | 9,446 ± 4,215 (8,400) | | 0.251 |
| LEUKOCYTES (D13) | | 51 | 7,367 ± 1,983 (6,900) | 8,090 ± 3,595 (7,600) | | 0.796 |
| LEUKOCYTES (D15) | | 169 | 9,461 ± 3,781 (8,600) | 9,330 ± 3,587 (8,400) | | 0.907 |
| LEUKOCYTES (D28 | | 130 | 8,173 ± 11,782 (6,300) | 6,305 ± 2,361 (6,150) | | 0.589 |
| PROCALCITONIN (D1) | | 168 | 0.12 ± 0.11 (0.10) | 0.11 ± 0.09 (0.09) | | 0.666 |
| PROCALCITONIN (D7) | | 102 | 0.10 ± 0.11 (0.07) | 0.06 ± 0.05 (0.05) | | **0.028** |
| PROCALCITONIN (D15) | | 145 | 0.10 ± 0.08 (0.10) | 0.09 ± 0.08 (0.07) | | 0.249 |
| PROCALCITONIN (D28) | | 125 | 0.06 ± 0.06 (0.03) | 0.07 ± 0.07 (0.05) | | 0.661 |
| ^1^Mean ± SD (Median) | | | | | | |
| ^2^Wilcoxon rank sum test; Wilcoxon rank sum exact test | | | | | | |

**Supplementary Table 9: Demonstration of immunological marker values during the study days.**

|  | TREATMENTS | | | |  | |
| --- | --- | --- | --- | --- | --- | --- |
| Overall | | N | FNC, N = 91^1^ | Placebo, N = 88^1^ | | p-value^2^ |
| LYMPHOCYTES [20-45%](D1) | | 179 | 1,120 ± 608 (990) | 1,101 ± 521 (1,004) | | 0.826 |
| LYMPHOCYTES [20-45%](D3) | | 178 | 1,200 ± 514 (1,133) | 1,202 ± 596 (1,120) | | 0.690 |
| LYMPHOCYTES [20-45%](D5) | | 165 | 1,302 ± 625 (1,224) | 1,426 ± 676 (1,331) | | 0.273 |
| LYMPHOCYTES [20-45%](D7) | | 133 | 1,933 ± 999 (1,813) | 2,016 ± 1,018 (1,845) | | 0.617 |
| LYMPHOCYTES [20-45%](D9) | | 98 | 1,657 ± 850 (1,536) | 1,681 ± 625 (1,494) | | 0.597 |
| LYMPHOCYTES [20-45%](D11) | | 79 | 1,392 ± 581 (1,374) | 1,579 ± 533 (1,512) | | 0.179 |
| LYMPHOCYTES [20-45%](D13) | | 51 | 1,342 ± 560 (1,302) | 1,697 ± 593 (1,589) | | **0.031** |
| LYMPHOCYTES [20-45%](D15) | | 169 | 1,573 ± 640 (1,414) | 1,647 ± 753 (1,548) | | 0.580 |
| LYMPHOCYTES [20-45%](D28 | | 130 | 2,061 ± 646 (2,031) | 1,953 ± 623 (1,933) | | 0.225 |
| CD3 (D1) | | 172 | 780 ± 463 (696) | 732 ± 395 (668) | | 0.635 |
| CD3 (D3) | | 175 | 820 ± 364 (741) | 779 ± 446 (652) | | 0.127 |
| CD3 (D5) | | 140 | 849 ± 424 (788) | 925 ± 445 (889) | | 0.310 |
| CD3 (D7) | | 109 | 1,341 ± 782 (1,118) | 1,415 ± 751 (1,348) | | 0.396 |
| CD3 (D9) | | 79 | 1,233 ± 704 (1,214) | 1,272 ± 462 (1,202) | | 0.425 |
| CD3 (D11) | | 63 | 1,021 ± 479 (942) | 1,226 ± 457 (1,149) | | 0.073 |
| CD3 (D13) | | 0 | NA ± NA (NA) | NA ± NA (NA) | |  |
| CD3 (D15) | | 166 | 1,175 ± 508 (1,082) | 1,218 ± 628 (1,132) | | 0.791 |
| CD3 (D28) | | 128 | 1,530 ± 550 (1,605) | 1,416 ± 559 (1,411) | | 0.103 |
| CD4 (D1) | | 172 | 499 ± 321 (426) | 455 ± 283 (362) | | 0.431 |
| CD4 (D3) | | 175 | 509 ± 231 (476) | 484 ± 320 (371) | | 0.059 |
| CD4 (D5) | | 140 | 521 ± 275 (478) | 578 ± 324 (515) | | 0.345 |
| CD4 (D7) | | 109 | 913 ± 554 (838) | 950 ± 564 (862) | | 0.649 |
| CD4 (D9) | | 79 | 809 ± 455 (652) | 833 ± 330 (708) | | 0.452 |
| CD4 (D11) | | 63 | 683 ± 296 (653) | 788 ± 337 (701) | | 0.268 |
| CD4 (D13) | | 0 | NA ± NA (NA) | NA ± NA (NA) | |  |
| CD4 (D15) | | 166 | 757 ± 37 (685) | 771 ± 440 (721) | | 0.950 |
| CD4 (D28) | | 128 | 899 ± 353 (925) | 800 ± 322 (822) | | **0.018** |
| CD8 (D1) | | 172 | 256 ± 175 (220) | 253 ± 152 (205) | | 0.725 |
| CD8 (D3) | | 175 | 292 ± 178 (246) | 275 ± 161 (253) | | 0.695 |
| CD8 (D5) | | 140 | 308 ± 184 (283) | 330 ± 181 (319) | | 0.368 |
| CD8 (D7) | | 109 | 406 ± 264 (337) | 445 ± 234 (402) | | 0.148 |
| CD8 (D9) | | 79 | 404 ± 256 (387) | 417 ± 190 (352) | | 0.438 |
| CD8 (D11) | | 63 | 327 ± 215 (278) | 412 ± 166 (410) | | **0.036** |
| CD8 (D13) | | 0 | NA ± NA (NA) | NA ± NA (NA) | |  |
| CD8 (D15) | | 166 | 386 ± 191 (349) | 415 ± 266 (343) | | 0.771 |
| CD8 (D28) | | 128 | 564 ± 252 (543) | 553 ± 298 (544) | | 0.485 |
| ^1^Mean ± SD (Median) | | | | | | |
| ^2^Wilcoxon rank sum test; Wilcoxon rank sum exact test | | | | | | |

**Supplementary Table 10: Demonstration of respiratory symptoms values during the study days**

|  | TREATMENTS | | | |  | |
| --- | --- | --- | --- | --- | --- | --- |
| Overall | | N | FNC, N = 91^1^ | Placebo, N = 88^1^ | | p-value^2^ |
| COUGH (Qty. days) | | 179 | 3.2 ± 3.6 (2.0) | 3.5 ± 3.4 (3.0) | | 0.305 |
| SMELL LOSS (Qty. days) | | 179 | 1.6 ± 3.4 (0.0) | 1.8 ± 3.1 (0.0) | | 0.347 |
| SORE THROAT (Qty. days) | | 179 | 0.74 ± 1.65 (0.00) | 1.13 ± 2.34 (0.00) | | 0.195 |
| CORYZA (Qty. days) | | 179 | 0.35 ± 0.91 (0.00) | 0.48 ± 1.09 (0.00) | | 0.392 |
| DYSPNEA (Qty. days) | | 179 | 0.86 ± 1.80 (0.00) | 0.95 ± 2.07 (0.00) | | 0.671 |
| TACHYPNEA (Qty. days) | | 179 | 0.32 ± 1.31 (0.00) | 0.31 ± 0.78 (0.00) | | 0.262 |
| ^1^Mean ± SD (Median) | | | | | | |
| ^2^Wilcoxon rank sum test | | | | | | |

**Supplementary Table 11: Demonstration of O2 saturation values during the study days**.

|  | TREATMENTS | | | |  | |
| --- | --- | --- | --- | --- | --- | --- |
| Overall | | N | FNC, N = 91^1^ | Placebo, N = 88^1^ | | p-value^2^ |
| O2 saturation (D1) | | 178 | 94.77 ± 2.33 (95.00) | 94.23 ± 8.84 (95.00) | | 0.255 |
| O2 saturation (D2) | | 179 | 95.35 ± 1.87 (95.00) | 95.27 ± 2.35 (95.00) | | 0.952 |
| O2 saturation (D3) | | 176 | 95.72 ± 1.64 (96.00) | 95.51 ± 2.03 (96.00) | | 0.652 |
| O2 saturation (D4) | | 173 | 96.19 ± 1.86 (96.00) | 95.80 ± 1.89 (96.00) | | 0.122 |
| O2 saturation (D5) | | 171 | 96.22 ± 1.94 (96.50) | 96.20 ± 1.84 (96.00) | | 0.757 |
| O2 saturation (D6) | | 145 | 96.31 ± 1.74 (97.00) | 96.38 ± 2.21 (97.00) | | 0.416 |
| O2 saturation (D7) | | 133 | 96.51 ± 1.87 (97.00) | 96.60 ± 1.97 (97.00) | | 0.567 |
| O2 saturation (D8) | | 108 | 96.00 ± 2.73 (97.00) | 96.52 ± 1.90 (97.00) | | 0.349 |
| O2 saturation (D9) | | 104 | 96.50 ± 1.88 (97.00) | 96.73 ± 1.57 (97.00) | | 0.582 |
| O2 saturation (D10) | | 81 | 96.46 ± 2.10 (97.00) | 96.66 ± 2.71 (97.00) | | 0.286 |
| O2 saturation (D11) | | 76 | 96.32 ± 2.20 (97.00) | 96.60 ± 1.87 (97.00) | | 0.608 |
| O2 saturation (D12) | | 60 | 96.20 ± 2.31 (97.00) | 96.20 ± 2.35 (97.00) | | 0.957 |
| O2 saturation (D13) | | 57 | 96.41 ± 2.30 (97.50) | 96.37 ± 3.77 (97.00) | | 0.634 |
| ^1^Mean ± SD (Median); n (%) | | | | | | |
| ^2^Wilcoxon rank sum test; Pearson's Chi-squared test; Fisher's exact test; Wilcoxon rank sum exact test | | | | | | |

**Supplementary Table 12: Demonstration of O2 saturation values during the study days**

|  | TREATMENTS | | | |  | |
| --- | --- | --- | --- | --- | --- | --- |
| Overall | | N | FNC, N = 91^1^ | Placebo, N = 88^1^ | | p-value^2^ |
| Respiratory frequency (D1) | | 178 | 20.56 ± 2.09 (20.00) | 21.12 ± 3.37 (20.00) | | 0.723 |
| Respiratory frequency (D2) | | 179 | 19.73 ± 2.21 (20.00) | 20.26 ± 3.18 (20.00) | | 0.746 |
| Respiratory frequency (D3) | | 177 | 19.46 ± 2.54 (20.00) | 19.45 ± 2.17 (20.00) | | 0.934 |
| Respiratory frequency (D4) | | 173 | 19.63 ± 2.61 (20.00) | 19.33 ± 2.36 (20.00) | | 0.521 |
| Respiratory frequency (D5) | | 171 | 19.18 ± 2.19 (20.00) | 19.31 ± 2.11 (20.00) | | 0.881 |
| Respiratory frequency (D6) | | 145 | 19.26 ± 2.09 (20.00) | 19.04 ± 2.96 (20.00) | | 0.267 |
| Respiratory frequency (D7) | | 134 | 19.36 ± 2.45 (20.00) | 19.07 ± 2.74 (20.00) | | 0.571 |
| Respiratory frequency (D8) | | 108 | 19.35 ± 2.36 (20.00) | 18.80 ± 2.59 (19.50) | | 0.161 |
| Respiratory frequency (D9) | | 104 | 19.21 ± 2.70 (20.00) | 18.94 ± 2.24 (20.00) | | 0.977 |
| Respiratory frequency (D10) | | 81 | 19.16 ± 3.55 (20.00) | 18.48 ± 2.20 (20.00) | | 0.569 |
| Respiratory frequency (D11) | | 76 | 19.24 ± 2.99 (20.00) | 18.69 ± 2.28 (20.00) | | 0.695 |
| Respiratory frequency (D12) | | 60 | 18.40 ± 2.31 (20.00) | 18.46 ± 2.47 (20.00) | | 0.924 |
| Respiratory frequency (D13) | | 57 | 18.59 ± 2.81 (20.00) | 18.34 ± 2.58 (20.00) | | 0.791 |
| Respiratory frequency (D14) | | 38 | 18.76 ± 2.33 (20.00) | 19.05 ± 2.85 (20.00) | | 0.962 |
| ^1^Mean ± SD (Median); n (%) | | | | | | |
| ^2^Wilcoxon rank sum test; Pearson's Chi-squared test; Fisher's exact test; Wilcoxon rank sum exact test | | | | | | |

**Supplementary Table 13: Demonstration of the distribution of participants in the type of ventilatory support during the study days.**

|  | TREATMENTS | | | |  | |
| --- | --- | --- | --- | --- | --- | --- |
| Overall | | N | FNC, N = 91^1^ | Placebo, N = 88^1^ | | p-value^2^ |
| O2 saturation type (D1) | | 178 |  |  | | **0.018** |
| AMBIENT AIR | |  | 52 (58%) | 44 (50%) | |  |
| NASAL CATHETER | |  | 36 (40%) | 32 (36%) | |  |
| RESERVOIR MASK | |  | 2 (2.2%) | 12 (14%) | |  |
| O2 saturation type (D2) | | 179 |  |  | | 0.146 |
| AMBIENT AIR | |  | 65 (71%) | 53 (60%) | |  |
| NASAL CATHETER | |  | 20 (22%) | 22 (25%) | |  |
| RESERVOIR MASK | |  | 6 (6.6%) | 13 (15%) | |  |
| O2 saturation type (D3) | | 176 |  |  | | 0.745 |
| AMBIENT AIR | |  | 70 (78%) | 63 (73%) | |  |
| NASAL CATHETER | |  | 13 (14%) | 16 (19%) | |  |
| RESERVOIR MASK | |  | 7 (7.8%) | 7 (8.1%) | |  |
| O2 saturation type (D4) | | 173 |  |  | | 0.355 |
| AMBIENT AIR | |  | 76 (85%) | 68 (81%) | |  |
| NASAL CATHETER | |  | 7 (7.9%) | 12 (14%) | |  |
| RESERVOIR MASK | |  | 6 (6.7%) | 4 (4.8%) | |  |
| O2 saturation type (D5) | | 171 |  |  | | 0.649 |
| AMBIENT AIR | |  | 80 (91%) | 72 (87%) | |  |
| NASAL CATHETER | |  | 6 (6.8%) | 7 (8.4%) | |  |
| RESERVOIR MASK | |  | 2 (2.3%) | 4 (4.8%) | |  |
| O2 saturation type (D6) | | 145 |  |  | | 0.477 |
| AMBIENT AIR | |  | 66 (89%) | 61 (86%) | |  |
| NASAL CATHETER | |  | 6 (8.1%) | 5 (7.0%) | |  |
| RESERVOIR MASK | |  | 2 (2.7%) | 5 (7.0%) | |  |
| O2 saturation type (D7) | | 133 |  |  | | 0.787 |
| AMBIENT AIR | |  | 61 (94%) | 61 (90%) | |  |
| NASAL CATHETER | |  | 3 (4.6%) | 4 (5.9%) | |  |
| RESERVOIR MASK | |  | 1 (1.5%) | 3 (4.4%) | |  |
| O2 saturation type (D8) | | 108 |  |  | | 0.896 |
| AMBIENT AIR | |  | 49 (91%) | 47 (87%) | |  |
| NASAL CATHETER | |  | 3 (5.6%) | 3 (5.6%) | |  |
| RESERVOIR MASK | |  | 2 (3.7%) | 4 (7.4%) | |  |
| O2 saturation type (D9) | | 104 |  |  | | 0.715 |
| AMBIENT AIR | |  | 48 (92%) | 47 (90%) | |  |
| NASAL CATHETER | |  | 3 (5.8%) | 5 (9.6%) | |  |
| RESERVOIR MASK | |  | 1 (1.9%) | 0 (0%) | |  |
| O2 saturation type (D10) | | 81 |  |  | | >0.999 |
| AMBIENT AIR | |  | 34 (92%) | 40 (91%) | |  |
| NASAL CATHETER | |  | 2 (5.4%) | 3 (6.8%) | |  |
| RESERVOIR MASK | |  | 1 (2.7%) | 1 (2.3%) | |  |
| O2 saturation type (D11) | | 76 |  |  | | 0.811 |
| AMBIENT AIR | |  | 32 (94%) | 38 (90%) | |  |
| NASAL CATHETER | |  | 1 (2.9%) | 3 (7.1%) | |  |
| RESERVOIR MASK | |  | 1 (2.9%) | 1 (2.4%) | |  |
| O2 saturation type (D12) | | 60 |  |  | | >0.999 |
| AMBIENT AIR | |  | 24 (96%) | 33 (94%) | |  |
| NASAL CATHETER | |  | 1 (4.0%) | 2 (5.7%) | |  |
| O2 saturation type (D13) | | 57 |  |  | | >0.999 |
| AMBIENT AIR | |  | 21 (95%) | 32 (91%) | |  |
| NASAL CATHETER | |  | 1 (4.5%) | 2 (5.7%) | |  |
| RESERVOIR MASK | |  | 0 (0%) | 1 (2.9%) | |  |
| O2 saturation type (D14) | | 38 |  |  | | >0.999 |
| AMBIENT AIR | |  | 17 (100%) | 20 (95%) | |  |
| RESERVOIR MASK | |  | 0 (0%) | 1 (4.8%) | |  |
| ^1^Mean ± SD (Median); n (%) | | | | | | |
| ^2^Wilcoxon rank sum test; Pearson's Chi-squared test; Fisher's exact test; Wilcoxon rank sum exact test | | | | | | |

**Supplementary Table 14: Demonstration of O2 consumption during ventilatory support on the study days.**

|  | TREATMENTS | | | |  | |
| --- | --- | --- | --- | --- | --- | --- |
| Overall | | N | FNC, N = 91^1^ | Placebo, N = 88^1^ | | p-value^2^ |
| O2 (L/min) (D1) | | 82 | 4.18 ± 2.36 (4.00) | 5.07 ± 3.39 (4.00) | | 0.374 |
| O2 (L/min) (D2) | | 61 | 5.1 ± 3.1 (5.0) | 5.7 ± 3.8 (5.0) | | 0.825 |
| O2 (L/min) (D3) | | 43 | 5.3 ± 3.7 (4.0) | 6.2 ± 4.6 (5.0) | | 0.749 |
| O2 (L/min) (D4) | | 29 | 5.2 ± 4.7 (3.0) | 5.6 ± 3.7 (5.0) | | 0.398 |
| O2 (L/min) (D5) | | 19 | 5.62 ± 3.89 (4.00) | 5.45 ± 2.50 (4.00) | | 0.830 |
| O2 (L/min) (D6) | | 18 | 5.12 ± 4.19 (3.50) | 4.90 ± 2.64 (4.50) | | 0.893 |
| O2 (L/min) (D7) | | 11 | 6.0 ± 6.1 (3.5) | 5.7 ± 2.7 (5.0) | | 0.633 |
| O2 (L/min) (D8) | | 12 | 5.60 ± 5.94 (2.00) | 5.43 ± 0.79 (6.00) | | 0.619 |
| O2 (L/min) (D9) | | 9 | 5.75 ± 6.24 (3.00) | 4.60 ± 1.52 (5.00) | | 0.535 |
| O2 (L/min) (D10) | | 7 | 7.3 ± 6.8 (5.0) | 3.8 ± 3.4 (3.0) | | 0.471 |
| O2 (L/min) (D11) | | 6 | 10.0 ± 7.1 (10.0) | 4.0 ± 2.8 (3.0) | | 0.240 |
| O2 (L/min) (D12) | | 3 | 3.00 ± NA (3.00) | 4.00 ± 1.41 (4.00) | | >0.999 |
| O2 (L/min) (D13) | | 4 | 3.00 ± NA (3.00) | 3.67 ± 2.52 (4.00) | | >0.999 |
| ^1^Mean ± SD (Median); n (%) | | | | | | |
| ^2^Wilcoxon rank sum test; Pearson's Chi-squared test; Fisher's exact test; Wilcoxon rank sum exact test | | | | | | |

**Supplementary Table 15: Demonstration of the use of mechanical ventilation during the study days.**

|  | TREATMENTS | | | |  | |
| --- | --- | --- | --- | --- | --- | --- |
| Overall | | N | FNC, N = 91^1^ | Placebo, N = 88^1^ | | p-value^2^ |
| Mechanical ventilation (D1) Y/N | | 178 | 0 (0%) | 0 (0%) | |  |
| Mechanical ventilation (D2) Y/N | | 179 | 0 (0%) | 0 (0%) | |  |
| Mechanical ventilation (D3) Y/N | | 177 | 1 (1.1%) | 0 (0%) | | >0.999 |
| Mechanical ventilation (D4) Y/N | | 173 | 0 (0%) | 0 (0%) | |  |
| Mechanical ventilation (D5) Y/N | | 171 | 0 (0%) | 0 (0%) | |  |
| Mechanical ventilation (D6) Y/N | | 145 | 0 (0%) | 0 (0%) | |  |
| Mechanical ventilation (D7) Y/N | | 134 | 1 (1.5%) | 0 (0%) | | 0.493 |
| Mechanical ventilation (D8) Y/N | | 108 | 0 (0%) | 0 (0%) | |  |
| Mechanical ventilation (D9) Y/N | | 104 | 0 (0%) | 0 (0%) | |  |
| Mechanical ventilation (D10) Y/N | | 81 | 0 (0%) | 0 (0%) | |  |
| Mechanical ventilation (D11) Y/N | | 76 | 0 (0%) | 0 (0%) | |  |
| Mechanical ventilation (D12) Y/N | | 60 | 0 (0%) | 0 (0%) | |  |
| Mechanical ventilation (D13) Y/N | | 57 | 0 (0%) | 0 (0%) | |  |
| Mechanical ventilation (D14) Y/N | | 38 | 0 (0%) | 0 (0%) | |  |
| ^1^Mean ± SD (Median); n (%) | | | | | | |
| ^2^Wilcoxon rank sum test; Pearson's Chi-squared test; Fisher's exact test; Wilcoxon | | | | | | |

**C-reactive protein analysis**

**Table:** Effects of time and treatments in relation to the variation of C-reactive protein results.

| Source of variation | Sum of Squares | DF | Mean Square | F | p |
| --- | --- | --- | --- | --- | --- |
| Time | 3396.5232 | 1 | 3396.5232 | 145.59562 | <0.001 |
| Treatment x Time | 0.0235 | 1 | 0.0235 | 0.00101 | 0.975 |
| Residual | 3569.2562 | 153 | 23.3285 |  |  |

**Table:** Effects of treatments in relation to the variation of C-reactive protein results.

| Source of variation | Sum of Squares | DF | Mean Square | F | p |
| --- | --- | --- | --- | --- | --- |
| Treatments | 42.7 | 1 | 42.7 | 1.38 | <0.242 |
| Residual | 4739.6 | 153 | 31.0 |  |  |

**Figure:** Median ± SD values of C-Reactive Protein in patients treated with Azudine and Placebo, between D1 and D15.

**REFERENCES**

^1^Chow S. C., Shao, J., Wang, H., Lokhnygina, Y. Eds. Sample Size Calculations in Clinical Research: Third Edition. 2017, Chapman and Hall/CRC.) utilizando a fórmula 4.2.2 (test for non-inferiority /superiority

2 Ren Z., Luo H., Yu Z., Song J., Liang L., Wang L., et al. A Randomized, Open-label, Controlled Clinical Trial of AZVUDINE Tablets in the Treatment of Mild and Common COVID-19, A Pilot Study. Adv Sci (Weinh). 2020 Jul 14,7(19):2001435. doi: 10.1002/advs.202001435. Epub ahead of print. PMID: 32837847, PMCID: PMC7404576.

^3^ Guieu J. D. and Hellon R. F., 1980. The Chill Sensation in Fever. Pflugers Arch. 384, 103- i04 (1980)

^4^ Kluger M J, Kozak W, Conn C A, Leon L R, Soszynski D., 1998. Ann N Y Acad Sci;856:224-33. doi: 10.1111/j.1749-6632.1998.tb08329.x.
