# Supplementary material for: Phase III, randomized, double-blind, placebo-controlled clinical study: a study on the safety and clinical efficacy of AZVUDINE in moderate COVID-19 patients
Source: Front Med (Lausanne). 2023 Oct 19;10:1215916. doi: 10.3389/fmed.2023.1215916 (PMC10620601; doi:10.3389/fmed.2023.1215916)
Supplement: Supplementary file 3 [file Data_Sheet_3.pdf]

SUPPLEMENTARY INFORMATION

1. SUPLEMENTARY METHODS

Identification of the dose to be used in the study

Pre-clinical trials were carried out for much longer than suggested, leading to a total human dose equivalent.

Table: Dose ratio and time of use

Animal/human equivalent dose	Animal / Time of drug use	Total dose used in preclinical tests over time
3.72 mg	**1 month**	116.6 mg
5.6 mg	**3 months**	504.0 mg
3.36 mg	**6,5 meses (26 weeks)**	655,20 mg
3.72 mg	**9,75 meses (39 weeks)**	1.088,10 mg

In preclinical trials, exposure to the drug for a prolonged period of time has shown that of the observable adverse events appear when the dose is exceeded by 20-30 times, but as the tests were carried out for a much longer time than suggested, AZVUDINE proved to be safe at the dose used.

For the IGZ-1 study, the proposed treatment period is 14 days (in view of the average incubation time of the coronavirus) and the maximum dose of Azvudine used in the 14 days is 70mg, remembering that the dose may be lower, because the participant may be discharged earlier.

The results of the pre-clinical study of FNC, in dogs, show that NOAEL=0.1mg/kg. Using the calculation formula 60×{[NOAEL]/(human kg/animal kg)}/10}) to calculate the equivalent dosage corresponding to the human body based on the NOAEL dosage of the most sensitive species and then dividing by safety coefficient (10) to obtain the initial dosage for the human body, the initial dosage for the human body was 0.36mg.

According to long-term toxicity data from dogs, taking 1/3 dose and maximum tolerance 0.3mg/kg, that is, 0.1mg/kg, to calculate human dosage (weight was calculated as 60kg), the maximum test dose would be 6mg/human/day.

As for the safety profile related to the dose used in humans, the maximum study of the safety dose of AZVUDINE was carried out for 5 mg, and a daily dose of 5 mg has an average life of 13.8 h, being excreted in the urine in up to 24 hours.

The proposed therapeutic regimen for the phase III clinical trial in patients with COVID-19 is based on preliminary studies conducted in China in 41 patients with pneumonia caused by SARS-CoV-2. The dose used was 5 mg/day, and the observed AEs were mild to moderate, with no need for medical intervention. All patients were discharged after two consecutive RT-PCR tests.

Dose explorer phase I clinical trial (GQ-FNC-2014) evaluated the safety of Azvudine in HIV-positive patients for 7 days and demonstrated that Azvudine has clinical safety. **Azvudine regiment dose groups for 2mg, 3mg, 4mg and 5mg showed good safety.** No participants in the Azvudine 3mg and 5mg groups reported any AEs and no serious adverse events were attributed to Azvudine. 8 patients with HIV received a dose of Azvudine 5mg, and no adverse drug reactions were observed. In these patients the plasma concentration of Azvudine was similar to the concentration observed in patients who received a dose of 2 to 4 mg. **Furthermore, the 5 mg dose led to a human plasma concentration (2.42 ng/mL)** which is 3,099 times lower than the concentration at the dose used in the CHL cell chromosome structure aberration test at a concentration of 7.5µg/mL after 4 hour exposure with or without S9 metabolic activation system.

Based on these studies, there was evidence that the 5 mg dose would have a good safety profile commensurate with use in the proposed target population.

2. SUPPLEMENTARY RESULTS

CLINICAL PICTURE	DESCRIPTION	SCORE
Uninfected	Non-infected; no viral RNA detection.	0
Outpatient: mild illness	Asymptomatic; viral RNA detected.	1
	Symptomatic; does not require assistance.	2
	Symptomatic; needs assistance.	3
Hospitalized: moderate illness	Hospitalized; no oxygen therapy*	4
	Hospitalized; oxygen support by mask or nasal catheter.	5
Hospitalized: serious illness	Hospitalized; oxygen support by NIV or high flow.	6
	Intubation and mechanical ventilation, pO2/FiO2 150 or SpO2/FiO2 □□200.	7
	Mechanical ventilation pO2/FiO2 <150 (SpO2/FiO2 <200) or vasopressors.	8
	Mechanical ventilation pO2/FiO2 <150 and vasopressors, dialysis or ECMO.	9
Death	Death.	10

Supplementary Figure 1: scores of the study related to clinical picture. *Table 4: WHO Clinical Progression Scale (Jun 2020)*

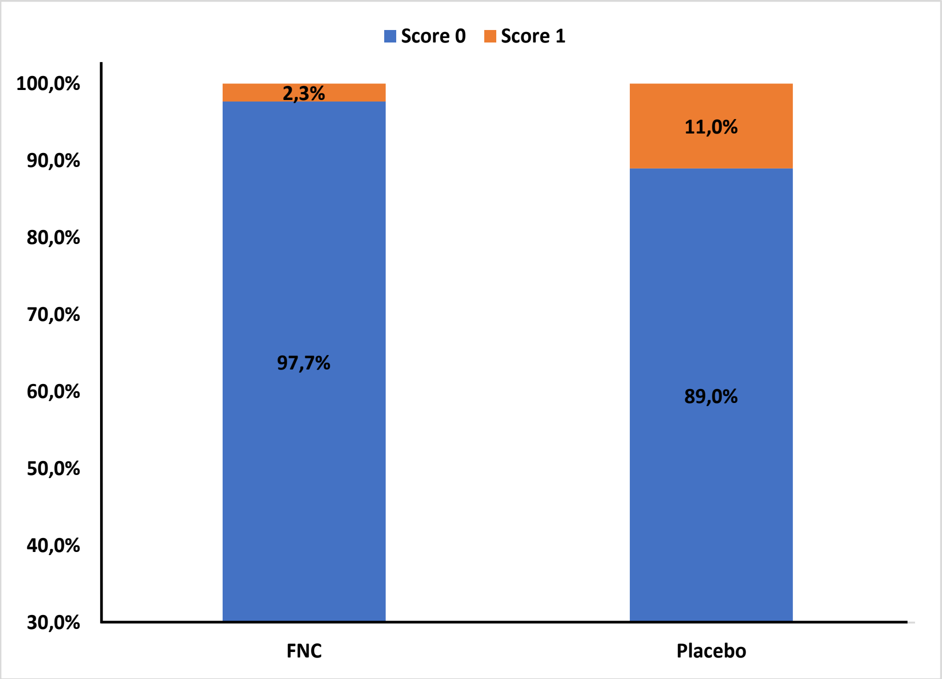

Supplementary Figure 2: Proportion of the initial and final clinical score of all subjects in the FNC group and the placebo group. The p value represents the significant difference between groups by the Wilcoxon test (Left bar: FNC; Right bar: PLACEBO).

Supplementary Table 1: **Time for improvement of symptoms.**

		TREATMENTS		
Overall	N	FNC, N = 91[1]	Placebo, N = 88[1]	p-value[2]
COUGH (Qty. days)	179	3.2 ± 3.6 (2.0)	3.5 ± 3.4 (3.0)	0.305
MYALGIA (Qty. days)	179	1.59 ± 3.00 (0.00)	1.83 ± 2.79 (0.00)	0.259
SMELL LOSS (Qty. days)	179	1.6 ± 3.4 (0.0)	1.8 ± 3.1 (0.0)	0.347
TASTE LOSS (Qty. days)	179	1.6 ± 3.3 (0.0)	1.7 ± 3.1 (0.0)	0.479
DIARRHEA (Qty. days)	179	0.16 ± 0.70 (0.00)	0.13 ± 0.50 (0.00)	0.981
DIZZINESS (Qty. days)	179	0.19 ± 1.09 (0.00)	0.20 ± 0.86 (0.00)	0.531
FEVER (Qty. days)	179	0.13 ± 0.50 (0.00)	0.38 ± 0.68 (0.00)	**<0.001**
CHILL (Qty. days)	179	0.21 ± 0.64 (0.00)	0.76 ± 1.95 (0.00)	**0.008**
SORE THROAT (Qty. days)	179	0.74 ± 1.65 (0.00)	1.13 ± 2.34 (0.00)	0.195
CORYZA (Qty. days)	179	0.35 ± 0.91 (0.00)	0.48 ± 1.09 (0.00)	0.392
DYSPNEA (Qty. days)	179	0.86 ± 1.80 (0.00)	0.95 ± 2.07 (0.00)	0.671
TACHYPNEA (Qty. days)	179	0.32 ± 1.31 (0.00)	0.31 ± 0.78 (0.00)	0.262
NAUSEA (Qty. days)	179	0.03 ± 0.18 (0.00)	0.10 ± 0.40 (0.00)	0.174
VOMIT (Qty. days)	179	0.0440 ± 0.2061 (0.0000)	0.0568 ± 0.3165 (0.0000)	0.764
ABDOMINAL PAIN (Qty. days)	179	0.1319 ± 0.6533 (0.0000)	0.0455 ± 0.2586 (0.0000)	0.488
INABILITY TO MARCH (Qty. days)	179	0.0549 ± 0.2734 (0.0000)	0.1023 ± 0.6072 (0.0000)	0.770

[1]Mean ± SD (Median)

[2]Wilcoxon rank sum test

Supplementary Table 2: Time to improve lung image enhancement.

		TREATMENTS		
Overall	N	FNC, N = 91[1]	Placebo, N = 88[1]	p-value[2]
CT Scan (D1)	152	30 ± 24 (30)	34 ± 25 (30)	0.530
CT Scan (D3)	161	31 ± 24 (30)	34 ± 24 (30)	0.483
CT Scan (D7)	114	33 ± 24 (30)	35 ± 24 (32)	0.602
CT Scan (D11)	64	30 ± 24 (25)	35 ± 22 (30)	0.297
CT Scan (D15)	163	22 ± 22 (20)	20 ± 23 (5)	0.512
CT Scan (D28)	133	6 ± 11 (1)	6 ± 11 (1)	0.935

[1]Mean ± SD (Median); n (%)

[2]Wilcoxon rank sum test; Pearson's Chi-squared test; Fisher's exact test; Wilcoxon rank sum exact test

RT-PCR

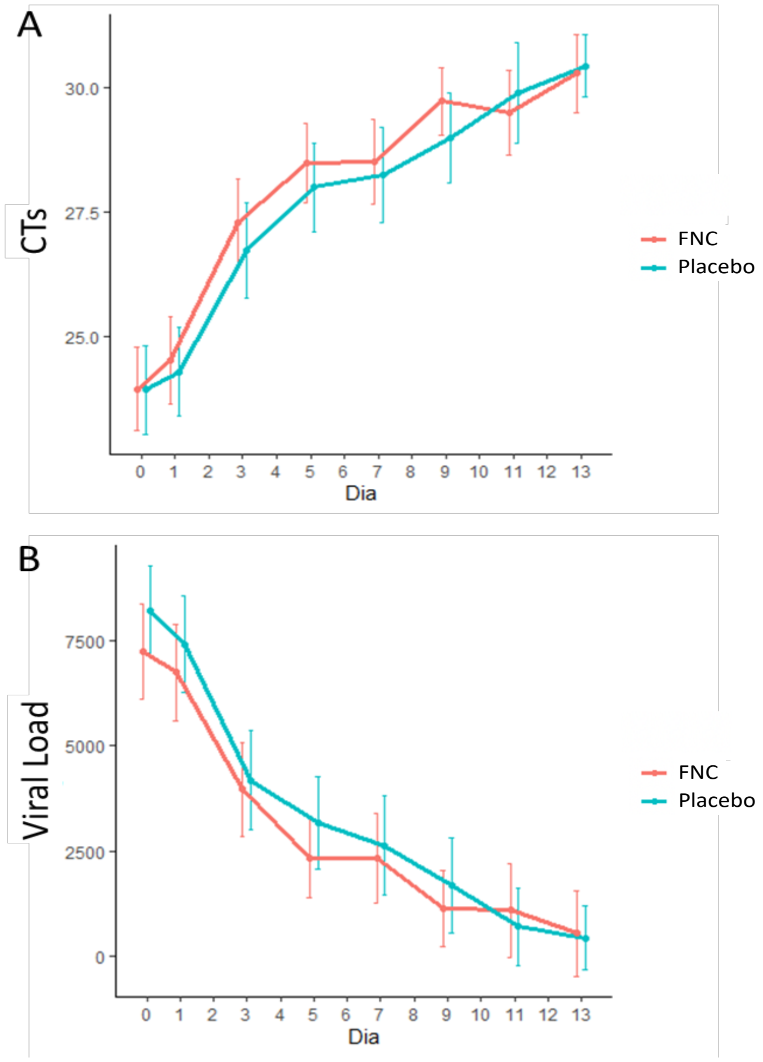

Supplementary Figure 3: CTs (A) and viral load (B) analysis measured by RT-PCR of all participants in the FNC group and the placebo group. Data are median (SD). (Red line: FNC; Blue line: PLACEBO).

Supplementary Table 3: Comorbidities

			TREATMENTS		
Overall	N	Total	FNC, N = 91[1]	Placebo, N = 88[1]	p-value[2]
Arterial hypertension	180	76 (42%)	37 (41%)	39 (44%)	0.799
Obesity	180	45 (25%)	21 (23%)	24 (27%)	0.704
Hepatic steatosis	180	0 (0%)	0 (0%)	0 (0%)	
Type 1 diabetes	180	4 (2.2%)	3 (3.3%)	1 (1.1%)	0.629
Type 2 diabetes	180	30 (17%)	12 (13%)	18 (20%)	0.360
Smoker	180	3 (1.7%)	1 (1.1%)	2 (2.3%)	0.623
Alcoholic	180	31 (17%)	11 (12%)	19 (22%)	**0.033**
Asthma	180	5 (2.8%)	3 (3.3%)	2 (2.3%)	>0.999
Bronchitis	180	10 (5.6%)	3 (3.3%)	6 (6.8%)	**0.026**
Cardiac arrhythmia	180	7 (3.9%)	2 (2.2%)	5 (5.7%)	0.301
Angina	180	1 (0.6%)	0 (0%)	1 (1.1%)	0.494
Kidney stone	180	0 (0%)	0 (0%)	0 (0%)	
Gallstone	180	1 (0.6%)	1 (1.1%)	0 (0%)	>0.999
Bariatric	180	0 (0%)	0 (0%)	0 (0%)	
Stroke	180	0 (0%)	0 (0%)	0 (0%)	
Vaccine	180	0 (0%)	0 (0%)	0 (0%)	
Cardiac revascularization	180	0 (0%)	0 (0%)	0 (0%)	
Stent	180	1 (0.6%)	1 (1.1%)	0 (0%)	>0.999
Osteoporosis	180	2 (1.1%)	1 (1.1%)	1 (1.1%)	>0.999

[1]n (%)
[2]Teste exato de Fisher

Supplementary Table 4: Demonstration of values for other variables during the days of study.

		TREATMENTS		
Overall	N	FNC, N = 91[1]	Placebo, N = 88[1]	p-value[2]
COUGH (Qty. days)	179	3.2 ± 3.6 (2.0)	3.5 ± 3.4 (3.0)	0.305
MYALGIA (Qty. days)	179	1.59 ± 3.00 (0.00)	1.83 ± 2.79 (0.00)	0.259
SMELL LOSS (Qty. days)	179	1.6 ± 3.4 (0.0)	1.8 ± 3.1 (0.0)	0.347
TASTE LOSS (Qty. days)	179	1.6 ± 3.3 (0.0)	1.7 ± 3.1 (0.0)	0.479
DIARRHEA (Qty. days)	179	0.16 ± 0.70 (0.00)	0.13 ± 0.50 (0.00)	0.981
DIZZINESS (Qty. days)	179	0.19 ± 1.09 (0.00)	0.20 ± 0.86 (0.00)	0.531
FEVER (Qty. days)	179	0.13 ± 0.50 (0.00)	0.38 ± 0.68 (0.00)	**<0.001**
CHILL (Qty. days)	179	0.21 ± 0.64 (0.00)	0.76 ± 1.95 (0.00)	**0.008**
SORE THROAT (Qty. days)	179	0.74 ± 1.65 (0.00)	1.13 ± 2.34 (0.00)	0.195
CORYZA (Qty. days)	179	0.35 ± 0.91 (0.00)	0.48 ± 1.09 (0.00)	0.392
DYSPNEA (Qty. days)	179	0.86 ± 1.80 (0.00)	0.95 ± 2.07 (0.00)	0.671
TACHYPNEA (Qty. days)	179	0.32 ± 1.31 (0.00)	0.31 ± 0.78 (0.00)	0.262
NAUSEA (Qty. days)	179	0.03 ± 0.18 (0.00)	0.10 ± 0.40 (0.00)	0.174
VOMIT (Qty. days)	179	0.0440 ± 0.2061 (0.0000)	0.0568 ± 0.3165 (0.0000)	0.764
ABDOMINAL PAIN (Qty. days)	179	0.1319 ± 0.6533 (0.0000)	0.0455 ± 0.2586 (0.0000)	0.488
INABILITY TO MARCH (Qty. days)	179	0.0549 ± 0.2734 (0.0000)	0.1023 ± 0.6072 (0.0000)	0.770

[1]Mean ± SD (Median)

[2]Wilcoxon rank sum test

Supplementary Table 5: Analysis of urinary phosphorus excretion in 24 hours.

		TREATMENTS		
Overall	N	FNC, N = 91[1]	Placebo, N = 88[1]	p-value[2]
URINE PHOSPHORUS 24h (D1)	149	423 ± 586 (28)	360 ± 534 (0)	0.361
URINE PHOSPHORUS 24h (D15)	55	43.79 ± 230.15 (3.20)	3.43 ± 0.51 (3.50)	0.263

[1]Mean ± SD (Median); n (%)

[2]Wilcoxon rank sum test; Wilcoxon rank sum exact test

Supplementary Table 6: Demonstration of serum cholinesterase values during the study days.

		TREATMENTS		
Overall	N	FNC, N = 91[1]	Placebo, N = 88[1]	p-value[2]
Cholinesterase (D1)	100	6,059 ± 6,672 (0)	3,816 ± 6,007 (0)	0.071
Cholinesterase (D15)	166	14,145 ± 15,225 (12,748)	12,502 ± 2,459 (12,744)	0.710
Cholinesterase (D28)	55	10,672 ± 6,156 (13,827)	9,496 ± 6,492 (12,045)	0.469

[1]Mean ± SD (Median); n (%)

[2]Wilcoxon rank sum test; Wilcoxon rank sum exact test

Supplementary Table 7: Demonstration of inflammatory marker values during the study days.

TREATMENTS				
Overall	N	FNC, N = 91[1]	Placebo, N = 88[1]	p-value[2]
INTERLEUKIN-6 (D1)	168	18 ± 31 (8)	25 ± 35 (10)	0.168
INTERLEUKIN-6 (D7)	106	18 ± 42 (4)	9 ± 12 (6)	0.709
INTERLEUKIN-6 (D15)	146	9 ± 13 (4)	8 ± 10 (4)	0.718
INTERLEUKIN-6 (D28)	128	3.97 ± 4.68 (2.60)	3.37 ± 2.40 (2.60)	0.747
CPK-MB (D1)	178	1.25 ± 1.68 (0.64)	0.99 ± 1.01 (0.70)	0.814
CPK-MB (D7)	109	0.60 ± 0.48 (0.45)	0.69 ± 0.81 (0.48)	0.891
CPK-MB (D15)	165	0.73 ± 0.70 (0.48)	0.60 ± 0.65 (0.29)	0.097
CPK-MB (D28)	108	1.64 ± 4.49 (0.66)	1.11 ± 2.56 (0.56)	0.978
TROPONIN I (D1)	179	0.18 ± 0.22 (0.10)	0.14 ± 0.10 (0.10)	0.192
TROPONIN I (D7)	107	0.17 ± 0.18 (0.10)	0.18 ± 0.28 (0.10)	0.594
TROPONIN I (D15)	155	0.18 ± 0.18 (0.10)	0.13 ± 0.09 (0.10)	0.086
TROPONIN I (D28)	106	0.47 ± 1.21 (0.10)	0.20 ± 0.32 (0.10)	0.413
Serum lactate (D1)	172	14.5 ± 5.5 (13.9)	13.9 ± 5.1 (13.3)	0.471
Serum lactate (D3)	174	18 ± 7 (17)	18 ± 8 (16)	0.824
Serum lactate (D5)	140	21 ± 9 (21)	19 ± 7 (18)	0.182
Serum lactate (D7)	109	19 ± 8 (18)	17 ± 6 (15)	0.300
Serum lactate (D9)	79	17 ± 8 (16)	16 ± 6 (15)	0.976
Serum lactate (D11)	63	13.7 ± 5.6 (12.5)	14.4 ± 4.6 (15.5)	0.231
Serum lactate (D13)	36	16 ± 7 (14)	15 ± 8 (13)	0.669
Serum lactate (D15)	164	17 ± 6 (16)	17 ± 10 (15)	0.692

TREATMENTS				
Overall	N	FNC, N = 91[1]	Placebo, N = 88[1]	p-value[2]
Activated partial thromboplastin time (D1)	179	34 ± 8 (32)	33 ± 6 (32)	0.795
Activated partial thromboplastin time (D15)	170	62 ± 310 (29)	28 ± 10 (30)	0.371
Activated partial thromboplastin time (D28)	130	32.7 ± 9.7 (31.6)	38.8 ± 35.8 (33.8)	**0.023**
Prothrombin time (D1)	179	11.92 ± 2.48 (11.50)	11.60 ± 1.71 (11.60)	0.603
Prothrombin time (D15)	170	10.4 ± 3.9 (11.5)	10.5 ± 3.5 (11.2)	0.499
Prothrombin time (D28)	130	14.49 ± 17.67 (11.30)	11.70 ± 1.33 (11.50)	0.540
Thrombin time (D1)	179	11.8 ± 8.2 (16.0)	12.6 ± 8.0 (17.0)	0.327
Thrombin time (D15)	170	14 ± 9 (18)	13 ± 8 (17)	0.293
Thrombin time (D28)	130	0.0000 ± 0.0000 (0.0000)	0.0000 ± 0.0000 (0.0000)	
Fibrinogen (D1)	179	473 ± 268 (522)	456 ± 260 (499)	0.509
Fibrinogen (D15)	170	175 ± 256 (0)	200 ± 258 (0)	0.433
Fibrinogen (D28)	130	0.0000 ± 0.0000 (0.0000)	0.0000 ± 0.0000 (0.0000)	
ESR (D1)	174	60 ± 27 (58)	53 ± 27 (46)	**0.047**
ESR (D7)	109	46 ± 28 (38)	45 ± 25 (42)	0.964
ESR (D15)	158	48 ± 25 (42)	52 ± 21 (52)	0.235
ESR (D28)	106	19 ± 15 (20)	19 ± 11 (18)	0.753
PLATELETS (D1)	179	288,857 ± 96,231 (279,000)	267,760 ± 87,731 (265,500)	0.154
PLATELETS (D3)	178	342,712 ± 111,911 (330,000)	323,898 ± 110,767 (309,000)	0.169
PLATELETS (D5)	165	395,475 ± 111,615 (390,000)	375,840 ± 122,612 (359,000)	0.173

TREATMENTS				
Overall	N	FNC, N = 91[1]	Placebo, N = 88[1]	p-value[2]
PLATELETS (D7)	133	404,002 ± 116,375 (382,500)	375,254 ± 115,688 (364,000)	0.148
PLATELETS (D9)	98	370,806 ± 128,207 (332,000)	359,204 ± 114,579 (362,000)	0.856
PLATELETS (D11)	79	342,444 ± 120,177 (334,500)	318,293 ± 126,402 (309,000)	0.400
PLATELETS (D13)	51	339,571 ± 133,038 (296,000)	280,413 ± 88,940 (287,000)	0.190
PLATELETS (D15)	169	364,739 ± 109,795 (357,500)	328,223 ± 108,760 (310,000)	**0.023**
PLATELETS (D28	130	280,422 ± 85,312 (284,000)	272,695 ± 80,559 (260,150)	0.434

[1]Mean ± SD (Median); n (%)

[2]Wilcoxon rank sum test; Wilcoxon rank sum exact test

Supplementary Table 8: Demonstration of white series values and immunological markers during the study days

		TREATMENTS		
Overall	N	FNC, N = 91[1]	Placebo, N = 88[1]	p-value[2]
LEUKOCYTES (D1)	179	9,015 ± 8,651 (7,700)	9,381 ± 3,748 (8,500)	**0.045**
LEUKOCYTES (D3)	178	10,459 ± 3,805 (10,220)	11,109 ± 4,602 (10,600)	0.448
LEUKOCYTES (D5)	165	11,833 ± 3,973 (11,400)	12,135 ± 3,584 (12,100)	0.425
LEUKOCYTES (D7)	133	10,172 ± 3,131 (10,000)	11,370 ± 3,626 (10,600)	0.087
LEUKOCYTES (D9)	98	9,096 ± 2,866 (9,400)	9,804 ± 3,453 (8,700)	0.614
LEUKOCYTES (D11)	79	8,181 ± 2,849 (8,000)	9,446 ± 4,215 (8,400)	0.251
LEUKOCYTES (D13)	51	7,367 ± 1,983 (6,900)	8,090 ± 3,595 (7,600)	0.796
LEUKOCYTES (D15)	169	9,461 ± 3,781 (8,600)	9,330 ± 3,587 (8,400)	0.907
LEUKOCYTES (D28	130	8,173 ± 11,782 (6,300)	6,305 ± 2,361 (6,150)	0.589
PROCALCITONIN (D1)	168	0.12 ± 0.11 (0.10)	0.11 ± 0.09 (0.09)	0.666
PROCALCITONIN (D7)	102	0.10 ± 0.11 (0.07)	0.06 ± 0.05 (0.05)	**0.028**
PROCALCITONIN (D15)	145	0.10 ± 0.08 (0.10)	0.09 ± 0.08 (0.07)	0.249
PROCALCITONIN (D28)	125	0.06 ± 0.06 (0.03)	0.07 ± 0.07 (0.05)	0.661

[1]Mean ± SD (Median)

[2]Wilcoxon rank sum test; Wilcoxon rank sum exact test

Supplementary Table 9: Demonstration of immunological marker values during the study days.

TREATMENTS				
Overall	N	FNC, N = 91[1]	Placebo, N = 88[1]	p-value[2]
LYMPHOCYTES [20-45%](D1)	179	1,120 ± 608 (990)	1,101 ± 521 (1,004)	0.826
LYMPHOCYTES [20-45%](D3)	178	1,200 ± 514 (1,133)	1,202 ± 596 (1,120)	0.690
LYMPHOCYTES [20-45%](D5)	165	1,302 ± 625 (1,224)	1,426 ± 676 (1,331)	0.273
LYMPHOCYTES [20-45%](D7)	133	1,933 ± 999 (1,813)	2,016 ± 1,018 (1,845)	0.617
LYMPHOCYTES [20-45%](D9)	98	1,657 ± 850 (1,536)	1,681 ± 625 (1,494)	0.597
LYMPHOCYTES [20-45%](D11)	79	1,392 ± 581 (1,374)	1,579 ± 533 (1,512)	0.179
LYMPHOCYTES [20-45%](D13)	51	1,342 ± 560 (1,302)	1,697 ± 593 (1,589)	**0.031**
LYMPHOCYTES [20-45%](D15)	169	1,573 ± 640 (1,414)	1,647 ± 753 (1,548)	0.580
LYMPHOCYTES [20-45%](D28	130	2,061 ± 646 (2,031)	1,953 ± 623 (1,933)	0.225
CD3 (D1)	172	780 ± 463 (696)	732 ± 395 (668)	0.635
CD3 (D3)	175	820 ± 364 (741)	779 ± 446 (652)	0.127
CD3 (D5)	140	849 ± 424 (788)	925 ± 445 (889)	0.310
CD3 (D7)	109	1,341 ± 782 (1,118)	1,415 ± 751 (1,348)	0.396
CD3 (D9)	79	1,233 ± 704 (1,214)	1,272 ± 462 (1,202)	0.425
CD3 (D11)	63	1,021 ± 479 (942)	1,226 ± 457 (1,149)	0.073
CD3 (D13)	0	NA ± NA (NA)	NA ± NA (NA)	
CD3 (D15)	166	1,175 ± 508 (1,082)	1,218 ± 628 (1,132)	0.791
CD3 (D28)	128	1,530 ± 550 (1,605)	1,416 ± 559 (1,411)	0.103
CD4 (D1)	172	499 ± 321 (426)	455 ± 283 (362)	0.431

TREATMENTS				
Overall	N	FNC, N = 91[1]	Placebo, N = 88[1]	p-value[2]
CD4 (D3)	175	509 ± 231 (476)	484 ± 320 (371)	0.059
CD4 (D5)	140	521 ± 275 (478)	578 ± 324 (515)	0.345
CD4 (D7)	109	913 ± 554 (838)	950 ± 564 (862)	0.649
CD4 (D9)	79	809 ± 455 (652)	833 ± 330 (708)	0.452
CD4 (D11)	63	683 ± 296 (653)	788 ± 337 (701)	0.268
CD4 (D13)	0	NA ± NA (NA)	NA ± NA (NA)	
CD4 (D15)	166	757 ± 37 (685)	771 ± 440 (721)	0.950
CD4 (D28)	128	899 ± 353 (925)	800 ± 322 (822)	**0.018**
CD8 (D1)	172	256 ± 175 (220)	253 ± 152 (205)	0.725
CD8 (D3)	175	292 ± 178 (246)	275 ± 161 (253)	0.695
CD8 (D5)	140	308 ± 184 (283)	330 ± 181 (319)	0.368
CD8 (D7)	109	406 ± 264 (337)	445 ± 234 (402)	0.148
CD8 (D9)	79	404 ± 256 (387)	417 ± 190 (352)	0.438
CD8 (D11)	63	327 ± 215 (278)	412 ± 166 (410)	**0.036**
CD8 (D13)	0	NA ± NA (NA)	NA ± NA (NA)	
CD8 (D15)	166	386 ± 191 (349)	415 ± 266 (343)	0.771
CD8 (D28)	128	564 ± 252 (543)	553 ± 298 (544)	0.485

[1]Mean ± SD (Median)

[2]Wilcoxon rank sum test; Wilcoxon rank sum exact test

Supplementary Table 10: Demonstration of respiratory symptoms values during the study days

		TREATMENTS		
Overall	N	FNC, N = 91[1]	Placebo, N = 88[1]	p-value[2]
COUGH (Qty. days)	179	3.2 ± 3.6 (2.0)	3.5 ± 3.4 (3.0)	0.305
SMELL LOSS (Qty. days)	179	1.6 ± 3.4 (0.0)	1.8 ± 3.1 (0.0)	0.347
SORE THROAT (Qty. days)	179	0.74 ± 1.65 (0.00)	1.13 ± 2.34 (0.00)	0.195
CORYZA (Qty. days)	179	0.35 ± 0.91 (0.00)	0.48 ± 1.09 (0.00)	0.392
DYSPNEA (Qty. days)	179	0.86 ± 1.80 (0.00)	0.95 ± 2.07 (0.00)	0.671
TACHYPNEA (Qty. days)	179	0.32 ± 1.31 (0.00)	0.31 ± 0.78 (0.00)	0.262

[1]Mean ± SD (Median)

[2]Wilcoxon rank sum test

Supplementary Table 11: Demonstration of O2 saturation values during the study days.

		TREATMENTS		
Overall	N	FNC, N = 91[1]	Placebo, N = 88[1]	p-value[2]
O2 saturation (D1)	178	94.77 ± 2.33 (95.00)	94.23 ± 8.84 (95.00)	0.255
O2 saturation (D2)	179	95.35 ± 1.87 (95.00)	95.27 ± 2.35 (95.00)	0.952
O2 saturation (D3)	176	95.72 ± 1.64 (96.00)	95.51 ± 2.03 (96.00)	0.652
O2 saturation (D4)	173	96.19 ± 1.86 (96.00)	95.80 ± 1.89 (96.00)	0.122
O2 saturation (D5)	171	96.22 ± 1.94 (96.50)	96.20 ± 1.84 (96.00)	0.757
O2 saturation (D6)	145	96.31 ± 1.74 (97.00)	96.38 ± 2.21 (97.00)	0.416
O2 saturation (D7)	133	96.51 ± 1.87 (97.00)	96.60 ± 1.97 (97.00)	0.567
O2 saturation (D8)	108	96.00 ± 2.73 (97.00)	96.52 ± 1.90 (97.00)	0.349
O2 saturation (D9)	104	96.50 ± 1.88 (97.00)	96.73 ± 1.57 (97.00)	0.582
O2 saturation (D10)	81	96.46 ± 2.10 (97.00)	96.66 ± 2.71 (97.00)	0.286
O2 saturation (D11)	76	96.32 ± 2.20 (97.00)	96.60 ± 1.87 (97.00)	0.608
O2 saturation (D12)	60	96.20 ± 2.31 (97.00)	96.20 ± 2.35 (97.00)	0.957
O2 saturation (D13)	57	96.41 ± 2.30 (97.50)	96.37 ± 3.77 (97.00)	0.634

[1]Mean ± SD (Median); n (%)

[2]Wilcoxon rank sum test; Pearson's Chi-squared test; Fisher's exact test; Wilcoxon rank sum exact test

Supplementary Table 12: Demonstration of O2 saturation values during the study days

TREATMENTS				
Overall	N	FNC, N = 91[1]	Placebo, N = 88[1]	p-value[2]
Respiratory frequency (D1)	178	20.56 ± 2.09 (20.00)	21.12 ± 3.37 (20.00)	0.723
Respiratory frequency (D2)	179	19.73 ± 2.21 (20.00)	20.26 ± 3.18 (20.00)	0.746
Respiratory frequency (D3)	177	19.46 ± 2.54 (20.00)	19.45 ± 2.17 (20.00)	0.934
Respiratory frequency (D4)	173	19.63 ± 2.61 (20.00)	19.33 ± 2.36 (20.00)	0.521
Respiratory frequency (D5)	171	19.18 ± 2.19 (20.00)	19.31 ± 2.11 (20.00)	0.881
Respiratory frequency (D6)	145	19.26 ± 2.09 (20.00)	19.04 ± 2.96 (20.00)	0.267
Respiratory frequency (D7)	134	19.36 ± 2.45 (20.00)	19.07 ± 2.74 (20.00)	0.571
Respiratory frequency (D8)	108	19.35 ± 2.36 (20.00)	18.80 ± 2.59 (19.50)	0.161
Respiratory frequency (D9)	104	19.21 ± 2.70 (20.00)	18.94 ± 2.24 (20.00)	0.977
Respiratory frequency (D10)	81	19.16 ± 3.55 (20.00)	18.48 ± 2.20 (20.00)	0.569
Respiratory frequency (D11)	76	19.24 ± 2.99 (20.00)	18.69 ± 2.28 (20.00)	0.695
Respiratory frequency (D12)	60	18.40 ± 2.31 (20.00)	18.46 ± 2.47 (20.00)	0.924
Respiratory frequency (D13)	57	18.59 ± 2.81 (20.00)	18.34 ± 2.58 (20.00)	0.791
Respiratory frequency (D14)	38	18.76 ± 2.33 (20.00)	19.05 ± 2.85 (20.00)	0.962

[1]Mean ± SD (Median); n (%)

[2]Wilcoxon rank sum test; Pearson's Chi-squared test; Fisher's exact test; Wilcoxon rank sum exact test

Supplementary Table 13: Demonstration of the distribution of participants in the type of ventilatory support during the study days.

	TREATMENTS			
Overall	N	FNC, N = 91[1]	Placebo, N = 88[1]	p-value[2]
O2 saturation type (D1)	178			**0.018**
AMBIENT AIR		52 (58%)	44 (50%)	
NASAL CATHETER		36 (40%)	32 (36%)	
RESERVOIR MASK		2 (2.2%)	12 (14%)	
O2 saturation type (D2)	179			0.146
AMBIENT AIR		65 (71%)	53 (60%)	
NASAL CATHETER		20 (22%)	22 (25%)	
RESERVOIR MASK		6 (6.6%)	13 (15%)	
O2 saturation type (D3)	176			0.745
AMBIENT AIR		70 (78%)	63 (73%)	
NASAL CATHETER		13 (14%)	16 (19%)	
RESERVOIR MASK		7 (7.8%)	7 (8.1%)	
O2 saturation type (D4)	173			0.355
AMBIENT AIR		76 (85%)	68 (81%)	
NASAL CATHETER		7 (7.9%)	12 (14%)	
RESERVOIR MASK		6 (6.7%)	4 (4.8%)	
O2 saturation type (D5)	171			0.649
AMBIENT AIR		80 (91%)	72 (87%)	
NASAL CATHETER		6 (6.8%)	7 (8.4%)	
RESERVOIR MASK		2 (2.3%)	4 (4.8%)	
O2 saturation type (D6)	145			0.477

TREATMENTS				
Overall	N	FNC, N = 91[1]	Placebo, N = 88[1]	p-value[2]
AMBIENT AIR		66 (89%)	61 (86%)	
NASAL CATHETER		6 (8.1%)	5 (7.0%)	
RESERVOIR MASK		2 (2.7%)	5 (7.0%)	
O2 saturation type (D7)	133			0.787
AMBIENT AIR		61 (94%)	61 (90%)	
NASAL CATHETER		3 (4.6%)	4 (5.9%)	
RESERVOIR MASK		1 (1.5%)	3 (4.4%)	
O2 saturation type (D8)	108			0.896
AMBIENT AIR		49 (91%)	47 (87%)	
NASAL CATHETER		3 (5.6%)	3 (5.6%)	
RESERVOIR MASK		2 (3.7%)	4 (7.4%)	
O2 saturation type (D9)	104			0.715
AMBIENT AIR		48 (92%)	47 (90%)	
NASAL CATHETER		3 (5.8%)	5 (9.6%)	
RESERVOIR MASK		1 (1.9%)	0 (0%)	
O2 saturation type (D10)	81			>0.999
AMBIENT AIR		34 (92%)	40 (91%)	
NASAL CATHETER		2 (5.4%)	3 (6.8%)	
RESERVOIR MASK		1 (2.7%)	1 (2.3%)	
O2 saturation type (D11)	76			0.811
AMBIENT AIR		32 (94%)	38 (90%)	

		TREATMENTS		
Overall	N	FNC, N = 91[1]	Placebo, N = 88[1]	p-value[2]
NASAL CATHETER		1 (2.9%)	3 (7.1%)	
RESERVOIR MASK		1 (2.9%)	1 (2.4%)	
O2 saturation type (D12)	60			>0.999
AMBIENT AIR		24 (96%)	33 (94%)	
NASAL CATHETER		1 (4.0%)	2 (5.7%)	
O2 saturation type (D13)	57			>0.999
AMBIENT AIR		21 (95%)	32 (91%)	
NASAL CATHETER		1 (4.5%)	2 (5.7%)	
RESERVOIR MASK		0 (0%)	1 (2.9%)	
O2 saturation type (D14)	38			>0.999
AMBIENT AIR		17 (100%)	20 (95%)	
RESERVOIR MASK		0 (0%)	1 (4.8%)	

[1]Mean ± SD (Median); n (%)

[2]Wilcoxon rank sum test; Pearson's Chi-squared test; Fisher's exact test; Wilcoxon rank sum exact test

Supplementary Table 14: Demonstration of O2 consumption during ventilatory support on the study days.

		TREATMENTS		
Overall	N	FNC, N = 91[1]	Placebo, N = 88[1]	p-value[2]
O2 (L/min) (D1)	82	4.18 ± 2.36 (4.00)	5.07 ± 3.39 (4.00)	0.374
O2 (L/min) (D2)	61	5.1 ± 3.1 (5.0)	5.7 ± 3.8 (5.0)	0.825
O2 (L/min) (D3)	43	5.3 ± 3.7 (4.0)	6.2 ± 4.6 (5.0)	0.749
O2 (L/min) (D4)	29	5.2 ± 4.7 (3.0)	5.6 ± 3.7 (5.0)	0.398
O2 (L/min) (D5)	19	5.62 ± 3.89 (4.00)	5.45 ± 2.50 (4.00)	0.830
O2 (L/min) (D6)	18	5.12 ± 4.19 (3.50)	4.90 ± 2.64 (4.50)	0.893
O2 (L/min) (D7)	11	6.0 ± 6.1 (3.5)	5.7 ± 2.7 (5.0)	0.633
O2 (L/min) (D8)	12	5.60 ± 5.94 (2.00)	5.43 ± 0.79 (6.00)	0.619
O2 (L/min) (D9)	9	5.75 ± 6.24 (3.00)	4.60 ± 1.52 (5.00)	0.535
O2 (L/min) (D10)	7	7.3 ± 6.8 (5.0)	3.8 ± 3.4 (3.0)	0.471
O2 (L/min) (D11)	6	10.0 ± 7.1 (10.0)	4.0 ± 2.8 (3.0)	0.240
O2 (L/min) (D12)	3	3.00 ± NA (3.00)	4.00 ± 1.41 (4.00)	>0.999
O2 (L/min) (D13)	4	3.00 ± NA (3.00)	3.67 ± 2.52 (4.00)	>0.999

[1]Mean ± SD (Median); n (%)

[2]Wilcoxon rank sum test; Pearson's Chi-squared test; Fisher's exact test; Wilcoxon rank sum exact test

Supplementary Table 15: Demonstration of the use of mechanical ventilation during the study days.

TREATMENTS				
Overall	N	FNC, N = 91[1]	Placebo, N = 88[1]	p-value[2]
Mechanical ventilation (D1) Y/N	178	0 (0%)	0 (0%)	
Mechanical ventilation (D2) Y/N	179	0 (0%)	0 (0%)	
Mechanical ventilation (D3) Y/N	177	1 (1.1%)	0 (0%)	>0.999
Mechanical ventilation (D4) Y/N	173	0 (0%)	0 (0%)	
Mechanical ventilation (D5) Y/N	171	0 (0%)	0 (0%)	
Mechanical ventilation (D6) Y/N	145	0 (0%)	0 (0%)	
Mechanical ventilation (D7) Y/N	134	1 (1.5%)	0 (0%)	0.493
Mechanical ventilation (D8) Y/N	108	0 (0%)	0 (0%)	
Mechanical ventilation (D9) Y/N	104	0 (0%)	0 (0%)	
Mechanical ventilation (D10) Y/N	81	0 (0%)	0 (0%)	
Mechanical ventilation (D11) Y/N	76	0 (0%)	0 (0%)	
Mechanical ventilation (D12) Y/N	60	0 (0%)	0 (0%)	
Mechanical ventilation (D13) Y/N	57	0 (0%)	0 (0%)	
Mechanical ventilation (D14) Y/N	38	0 (0%)	0 (0%)	

		TREATMENTS		
Overall	N	FNC, N = 91[1]	Placebo, N = 88[1]	p-value[2]

[1]Mean ± SD (Median); n (%)

[2]Wilcoxon rank sum test; Pearson's Chi-squared test; Fisher's exact test; Wilcoxon

C-reactive protein analysis

Table: Effects of time and treatments in relation to the variation of C-reactive protein results.

Source of variation	Sum of Squares	DF	Mean Square	F	p
Time	3396.5232	1	3396.5232	145.59562	<0.001
Treatment x Time	0.0235	1	0.0235	0.00101	0.975
Residual	3569.2562	153	23.3285		

Table: Effects of treatments in relation to the variation of C-reactive protein results.

Source of variation	Sum of Squares	DF	Mean Square	F	p
Treatments	42.7	1	42.7	1.38	<0.242
Residual	4739.6	153	31.0		

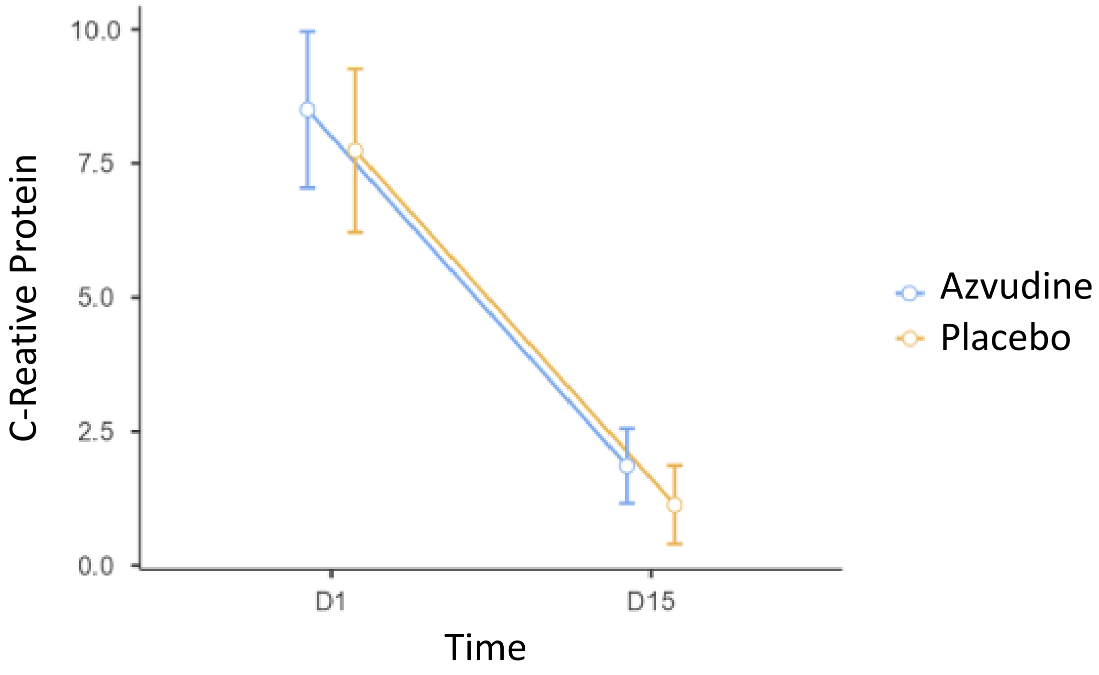

Figure: Median ± SD values of C-Reactive Protein in patients treated with Azudine and Placebo, between D1 and D15.

REFERENCES

[1]Chow S. C., Shao, J., Wang, H., Lokhnygina, Y. Eds. Sample Size Calculations in Clinical Research: Third Edition. 2017, Chapman and Hall/CRC.) utilizando a fórmula 4.2.2 (test for non-inferiority /superiority

[2] Ren Z., Luo H., Yu Z., Song J., Liang L., Wang L., et al. A Randomized, Open-label, Controlled Clinical Trial of AZVUDINE Tablets in the Treatment of Mild and Common COVID-19, A Pilot Study. Adv Sci (Weinh). 2020 Jul 14,7(19):2001435. doi: 10.1002/advs.202001435. Epub ahead of print. PMID: 32837847, PMCID: PMC7404576.

[3] Guieu J. D. and Hellon R. F., 1980. The Chill Sensation in Fever. Pflugers Arch. 384, 103- i04 (1980)

[4] Kluger M J, Kozak W, Conn C A, Leon L R, Soszynski D., 1998. Ann N Y Acad Sci;856:224-33. doi: 10.1111/j.1749-6632.1998.tb08329.x.
